# COVID-19 lockdowns reveal the resilience of Adriatic Sea fisheries to forced fishing effort reduction

**DOI:** 10.1038/s41598-022-05142-w

**Published:** 2022-01-20

**Authors:** Gianpaolo Coro, Anna Nora Tassetti, Enrico Nicola Armelloni, Jacopo Pulcinella, Carmen Ferrà, Mario Sprovieri, Fabio Trincardi, Giuseppe Scarcella

**Affiliations:** 1 Institute of Information Science and Technologies (ISTI), National Research Council of Italy (CNR), 56124 Pisa, Italy; 2 Institute for Biological Resources and Marine Biotechnology (IRBIM), National Research Council of Italy (CNR), 60125 Ancona, Italy; 3 University of Bologna, Department of Biological, Geological and Environmental Sciences, 40126 Bologna, Italy; 4 Institute of Anthropic Impacts and Sustainability in the Marine Environment (IAS), National Research Council of Italy (CNR), 91021 Torretta Granitola, Italy; 5 Department of Earth System Science and Environmental Technologies, National Research Council of Italy (CNR), 00185 Rome, Italy

**Keywords:** Computational biology and bioinformatics, Data mining, Statistical methods

## Abstract

The COVID-19 pandemic provides a major opportunity to study fishing effort dynamics and to assess the response of the industry to standard and remedial actions. Knowing a fishing fleet’s capacity to compensate for effort reduction (i.e., its resilience) allows differentiating governmental regulations by fleet, i.e., imposing stronger restrictions on the more resilient and weaker restrictions on the less resilient. In the present research, the response of the main fishing fleets of the Adriatic Sea to fishing hour reduction from 2015 to 2020 was measured. Fleet activity per gear type was inferred from monthly Automatic Identification System data. Pattern recognition techniques were applied to study the fishing effort trends and barycentres by gear. The beneficial effects of the lockdowns on Adriatic endangered, threatened and protected (ETP) species were also estimated. Finally, fleet effort series were examined through a stock assessment model to demonstrate that every Adriatic fishing fleet generally behaves like a stock subject to significant stress, which was particularly highlighted by the pandemic. Our findings lend support to the notion that the Adriatic fleets can be compared to predators with medium-high resilience and a generally strong impact on ETP species.

## Introduction

Large-scale collectors of vessel data (i.e., international agencies and industries) have estimated that the 2020 March–April lockdowns, imposed by several governments, reduced fishing activities by about 10% in the world and by about 50% in Europe^1^. In fact, on the one hand lockdowns introduced practical difficulties on board of vessels to applying social distances and, on the other hand, they caused a collapse of demand for seafood due to the closure of fish markets and restaurants and the reduction of direct sales, which in turn contributed to decrease fishing activities^2^. Both large and small-scale fisheries were affected, in different ways depending on the area^3,4^. Initially, the global reduction of fishing activity was due to the asynchronicity of the lockdown start time and fishing seasonality. Now, about two years into the pandemic, the time when this period began is becoming less and less relevant.

Understanding the response of fisheries to fishing hour reduction during the pandemic requires interpreting the information that vessels transmit via satellite and terrestrial devices, e.g., Automatic Identification Systems (AISs). To do this, fishing locations should be either extracted from the AIS records directly (when this information is available) or estimated from the sequences of coordinates embedded in these data (as in the case of the present experiment). Statistical and machine learning models are commonly used for these estimation tasks^5–7^ (Section 1.1 of Supplementary Information reports an overview). Although their operational error makes them better suited to large-scale and big data analysis^8,9^, they allow identifying fishing activity patterns over time.

During the COVID-19 pandemic, fisheries suffered a powerful stress due to the consequences of the sanitary restrictions. Quantifying this stress can allow gaining a greater understanding of the response of fisheries to regulations. We present a study of fisheries’ response to the reduction of fishing hours from 2015 to 2020, which was the result of a combination of factors, including the pandemic. A major hypothesis, which was verified by our approach, is that a fishing fleet can be modelled as a predator species exposed to external *stress factors* that reduce its abundance. Such factors include (Table 1-General factors) (i) low stock availability due to depletion; (ii) biological seasonality of the stock; (iii) regulatory seasonality due to monthly fishing hour limitations (like the summer fishing ban in the Adriatic Sea); (iv) vessel decommissioning, which reduces fleet capacity; (v) the response of the fish market to restrictions and the diminished fish supply; (vi) fishers’ reaction and low compliance with unpopular regulations. In this scenario, the COVID-19 dependent factors act as additional stress factors (Table 1-COVID-19 dependent factors) that may be viewed as equivalent to the effect that fishing pressure exerts on large predators. This hypothesis was tested using a stock assessment model (AMSY^10^). A spatial analysis was also performed, to identify the response of some fleets to the high stress level experienced due to the 2020 lockdowns and the sanitary restrictions. The fleets selected for this analysis were the large-scale fleets of the Adriatic Sea, the area with the highest regional bottom trawling footprint in the world^11^. In the Adriatic Sea, most of the fishing activity is carried out by four fleets targeting small pelagic and demersal fish species—pelagic pair trawlers, purse seiners, bottom otter trawlers and beam (or *Rapido*^12^) trawlers—which currently account for more than 70% of revenues^13^ (Section 1.2 of Supplementary Information reports an overview). Demersal target stocks usually have low-varying distributions over the years, whereas small pelagic stocks can largely vary their distributions^14^. Generally, all Adriatic stocks are constantly subject to fishing activity throughout the year.

**Table 1:** List of general and COVID-19 dependent factors that negatively affect the fisheries in the Adriatic Sea.

**General factors**	
Low stock availability due to depletion	
Biological seasonality of the stock	
Regulatory seasonality due to monthly fishing hour limitations	
Vessel decommissioning	
Response of the fish market to restrictions and diminished fish supply	
Fishers’ reaction and low compliance with unpopular regulations	
**COVID-19 dependent factors**	
Fish market closure	
Restaurant closure	
Reduction of intermediate and wholesaler activities	
Reduction of purchase for domestic use	
Consumption of frozen food instead of fresh food	
Sanitary restrictions on board	
Unsold catch	
Lowering of prices	
Liquidity requirements to manage money loss	
Forced stop of fishing activities	
Obligations of shifts for active vessels	
Fisheries’ self-fishing-activity limitation to reduce the offer	

Recent studies have highlighted the potential benefits brought by the temporary reduction of the impact of intense human activity on endangered, threatened and protected (ETP) species during the 2020 lockdowns^15–20^. The reduced impact has been estimated to increase species richness, abundance, breeding success, habitat quality, and reduce accidental capturing/killing. The rationale behind these positive expectations is that if a species was very concentrated in an intense human activity area, it was positively impacted by the 2020 lockdowns because its natural habitat was previously subject to mostly negative anthropogenic alterations^20,21^. In this paper, we examined whether the reduced fishing activity in 2020 also reduced the potential impact of fishing activities on ETP species.

Our analysis measured specific fleet traits that depend on fishing method (i.e., the gear used) and market capacity. These traits emerged from the similarity of the fleet effort dynamics to stock rebuilding dynamics. They summarise fleet responses to the *stress factors*, including the COVID-19 restrictions. The main traits analysed in each fleet were the speed of effort recovery (intrinsic growth rate) and the maximum number of fishing hours exceeding which regulations need to be introduced (carrying capacity).

The scenario emerging from our analysis is that the main Adriatic Sea fleets behave like apex predators towards their target stocks and that they show from medium to high resilience to effort reduction. Understanding and applying these findings has the potential to improve fishery and environmental management, minimise economic damage, maximise stock recovery and protect biodiversity. Notably, national supervisory bodies currently pursue stock rebuilding by mostly acting on the monthly fishing hours irrespective of fleet fishing method. Since different fleets suffer differently from impacts and have different recovery times, imposing stronger restrictions (e.g., longer closures or greater fishing hour reductions) on the more resilient and weaker restrictions on the less resilient would improve fishery management.

Altogether, our paper aims at answering the following specific research questions:

1. RQ1*: Did the lockdowns influence fishing activities in the Adriatic Sea and did these mirror broader patterns in the Italian seas?*
2. RQ2*: Which gear types were most affected by the 2020 lockdowns in the Adriatic Sea?*
3. RQ3*: Did the lockdowns change the potential impact of fisheries on ETP species?*
4. RQ4*: Can an Adriatic Sea fleet’s response to effort reduction be modelled as that of an apex-predator stock with measurable resilience and carrying capacity?*

## Methods

This section describes methodological steps. Primary data sources location, volume, and properties are detailed in Section 2 of the Supplementary Information (SI). Algorithmic details are reported in Section 3 of the SI.

## Vessel tracking data classification and analysis

We extracted coarse-scale patterns (0.5$^{\circ }$ spatial resolution) of fishing activity and activity change in all the Italian seas. Then, the analysis was specialised to the Adriatic Sea (at 0.1$^{\circ }$ spatial resolution), to establish whether a finer examination confirmed the coarse-scale observations. The larger-scale pattern analysis of the Italian seas was performed using aggregated fishing effort data from the Google’s Global Fishing Watch^6,22,23^ (GFW). The complete census of Automatic Identification System (AIS) transmission data from the Astra Paging collector^24^ was used to conduct a detailed analysis of fishing activity in the Adriatic basin. The AIS data contain high reporting frequency information (every 5 minutes per vessel) on vessel position, speed over the ground, course and rate of turn. We used the data of the EU Fleet Register^25^ (EUFR) to make a consistency check and a quantitative evaluation of AIS data quality (e.g., on the reported fleets’ size). Algorithmic details are given in Section 3.1-SI. The fishing gear types used by the AIS-equipped vessels were automatically inferred using a gear classification workflow based on speed and trajectory point distribution and cluster analysis (detailed in Section 3.2-SI). This workflow identified the gear that a vessel used in a trip among five types: bottom otter trawl (OTB), pelagic pair trawl (PTM), beam trawl (TBB), purse seine (PS) and other gear (OTHER). The classification performance was estimated against the gears reported in the vessel’s EUFR licence.

We used the GWF dataset to perform a large-scale analysis of fishing patterns at 0.5$^{\circ }$ spatial resolution in the Italian seas. In particular, we aggregated the GWF data in half-degree cells—for 1 March-30 April, for 2019, and for 2020—to identify coarse-scale fishing pattern changes during the 2020 lockdowns. We analysed the two datasets and classified fishing cells as low, medium and high-effort cells assuming a log-normal distribution of effort over the cells. Algorithmic details and pseudo-code are given in Section 3.3-SI. To obtain a more detailed classification of the fishing effort in the Adriatic Sea, we conducted the analysis at 0.1$^{\circ }$ resolution on the AIS dataset, to establish whether the changes detected in the GFW data could also be found in this dataset (i.e., to answer research question *RQ1*). We further explored the fishing activity patterns in the Adriatic Sea by calculating the barycentres of fishing activity of each gear over time using the AIS dataset (barycentre-calculation details are reported in Section 3.4-SI). The sequence of fishing effort barycentres over months of each individual fleet is a spatial time series that allows studying fishing vessel movements in the various seasons. In our experiment, this sequence also allowed assessing how the pandemic altered fishing activities in 2020 compared with the previous years. Conducting this analysis per gear type highlighted which were the most affected fleets by the 2020 lockdowns (i.e., this analysis answered research question *RQ2*).

**Figure 1:**
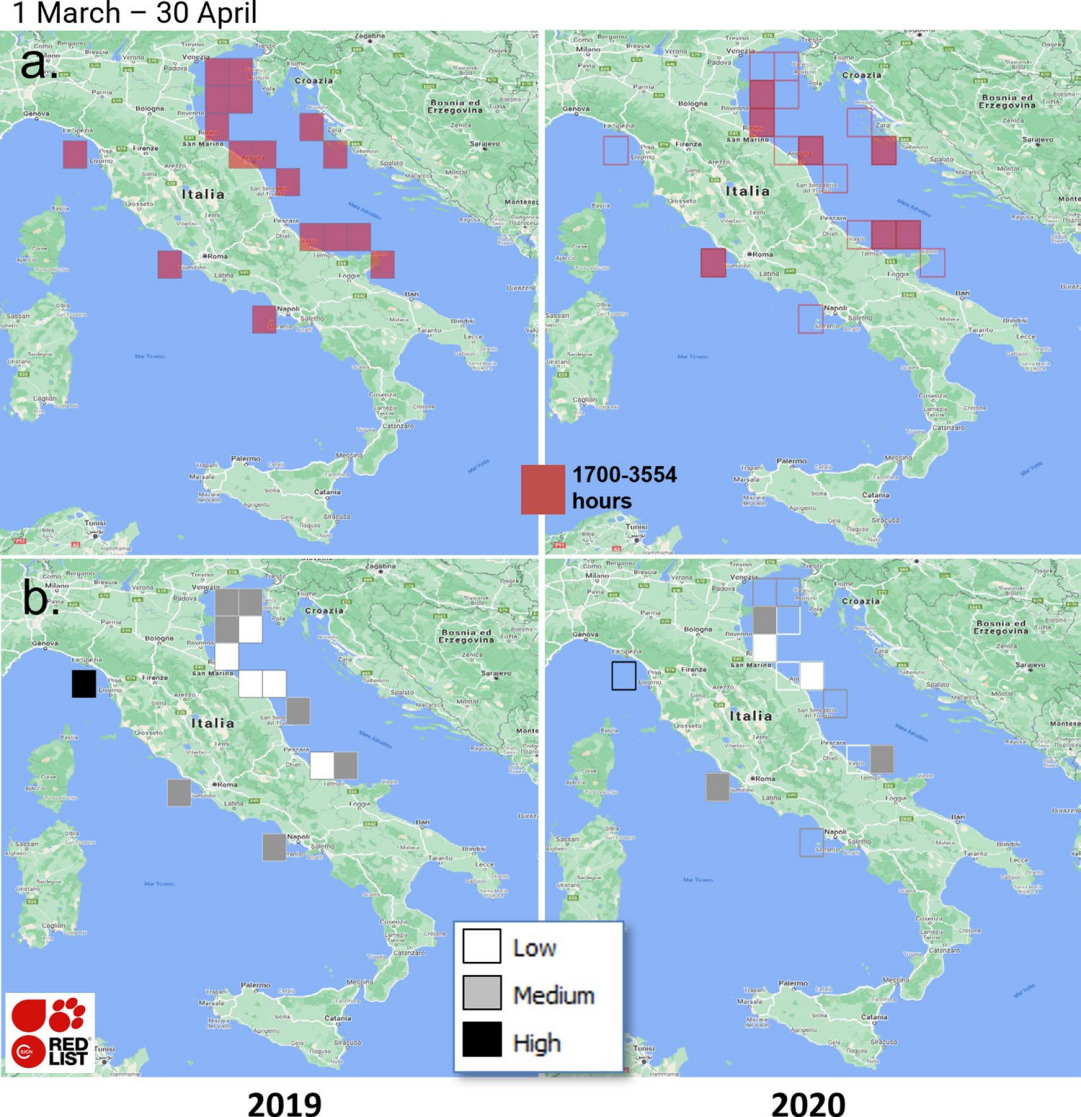
(**a**) Log-normal selection of 0.5$^{\circ }$ high-effort fishing locations, in the Italian seas, from Global Fishing Watch data from 1 March to 30 April. (**b**) Number (high, medium, low) of endangered, threatened and protected (ETP) species recorded at these locations. To enhance the comparison, the outlines of the 2019 cells (left) are reported in the 2020 maps (right). Maps were created with QGIS v.3.20.2 (www.qgis.org) using Google Maps-Satellite as the background map (version of 8 November 2021) (https://maps.google.com/).

Intersecting fishing activity locations with International Union for the Conservation of Nature^26^ (IUCN) information on the ETP species living at those sites allowed establishing the overlap between fishing activities and the species at risk, thus highlighting how their potential impact on such species changed during the pandemic (i.e., it allowed answering research question *RQ3*). IUCN data are authoritative and reliable to assess whether an observed species is an ETP species^27,28^ and have been already used in pattern analyses, e.g., to assess potential habitat change due to climate change^29^. The IUCN assessments are carried out by experts and specialists who have sufficient knowledge and data of the species, and are members of authoritative scientific commissions^30^. The United Nations General Assembly has granted observer status to IUCN and thus we considered it a reliable data source. The number of ETP species inhabiting high-effort cells was used to identify the locations where the potential impact was most severe. Several ETP species living in small areas in the Adriatic Sea are subject to intense fishing effort and high traffic. In particular, the total fishing hours can be as many as $\sim$ 20,000 h per month, mostly concentrated near the coasts (see also the “Results” section), with maximum recorded hours in one month of $\sim$ 7500 for beam trawlers, $\sim$ 1200 for pelagic pair trawlers, $\sim$ 12,000 for bottom otter trawlers and $\sim$ 900 for pure seiners. In one month, fishing hours can reach around 20 h per $km^2$ and $\sim$ 3500 h in 55 km squared areas^1,20^ (Fig. 1). Thus, this scenario has potentially adverse effects on species lives and habitats and justifies our analysis. The fishing cells were categorised as low-, medium-, and high-impact assuming a log-normal distribution of the number of ETP species per cell. Algorithmic details and pseudo-code are given in Section 3.5-SI. We conducted also this analysis in the Italian seas at 0.5$^{\circ }$ resolution using the GFW dataset, and in the Adriatic Sea at 0.1$^{\circ }$ resolution using the AIS dataset.

## Fleet assessment

The term *stock assessment* refers to the information scientists produce for fisheries management organisations, to inform periodical regulations, ensure fish availability on the market, and prevent stock depletion and economic difficulties. Stock assessment models estimate, among other key parameters, the maximum intrinsic rate of population size growth (*r*) and the unexploited population size that the environment can naturally sustain (carrying capacity, *k*). These two parameters allow estimating the maximum sustainable yield (*MSY*), i.e., the maximum amount of a stock’s biomass that can be taken without compromising the stocks’ ability to reconstitute it. A time series of catch statistics is a sequence of yields from the available stock biomass. Current data-limited stock assessment models can estimate the available annual stock biomass from the annual catch time series. They commonly rely on stock production models that correlate catch with biomass through *r* and *k* (Section 3.6-SI). The idea behind data-limited stock assessment models is that, if the catch is known and *r* and *k* can be estimated with acceptable approximation, it is possible to calculate the biomass evolution in time. The data-limited Abundance Maximum Sustainable Yield (AMSY) stock-assessment model^10^ uses an abundance index $A_t$ to reconstruct $Cq_t$, i.e., the catch time series $C_t$ multiplied by *catchability* *q*, a coefficient that reflects fishery efficiency (i.e., how much biomass becomes catch). AMSY uses the Schaefer stock dynamic equation to relate $A_t$ with $Cq_t$ (Section 3.6-SI). AMSY also estimates *MSY*, *r* and *kq* (the carrying capacity multiplied by catchability). If $A_t$ is a monthly time series (i.e., *t* is a monthly index), then *r* is its monthly growth and *kq* is the stock abundance in the absence of fishing pressure.

We used AMSY to study the Adriatic fleets’ response to regulations over time, particularly during 2020, and answer research question *RQ4*. We assumed each *fleet* (i.e., a set of vessels using the same gear) to be a stock, whose total monthly fishing hours were a measure of abundance $A_t$. Accordingly, the equivalent of the fishing pressure acting on the stock—whose effect is to reduce the population—is a combination of the *stress factors* that contribute to the reduction of the fleet’s fishing effort. If $A_t$ is the monthly fishing effort, then *r* represents the recovery speed of the fleet’s fishing effort, i.e., its response (including fishers’ reaction) to the combination of external factors that reduced its monthly fishing effort. Consequently, *kq* is the maximum number of hours the fleet can spend fishing before near saturation of its resources, i.e., the target stocks and the market. Beyond this number the fishery is no longer sustainable and regulations need to be introduced. According to this view, the catch $Cq_t$ is the reduction of fishing hours and *MSY* the maximum sustainable reduction of fishing hours that would not compromise the sustainability of the fishery with respect to its economic and natural resources (hereafter, *MSR*). The time series $Cmsr_t=\frac{C_t}{MSR}$ indicates the months $\{t\}$ when the fleet was over-stressed ($Cmsr_t>1$), under-stressed ($Cmsr_t<1$) or sustainably stressed ($Cmsr_t=1$). The ideal scenario would be to have regulations that keep fishing hours well under *kq* and do not exceed the *MSR*.

In summary, we estimated *r* and *kq* for the Adriatic fleets that employ OTB, PTM, TBB, and PS gears, and unfolded the differences between their growth rates and sustainable hours to produce novel information for fishery management. In this context, the reduction of fishing hours due to the pandemic was crucial to highlight data differences corresponding to the responses of the different fleets in 2020.

## Results

This section describes the results of our analyses. Further numerical details that support this analysis are reported in Section 4 of the SI.

**Figure 2:**
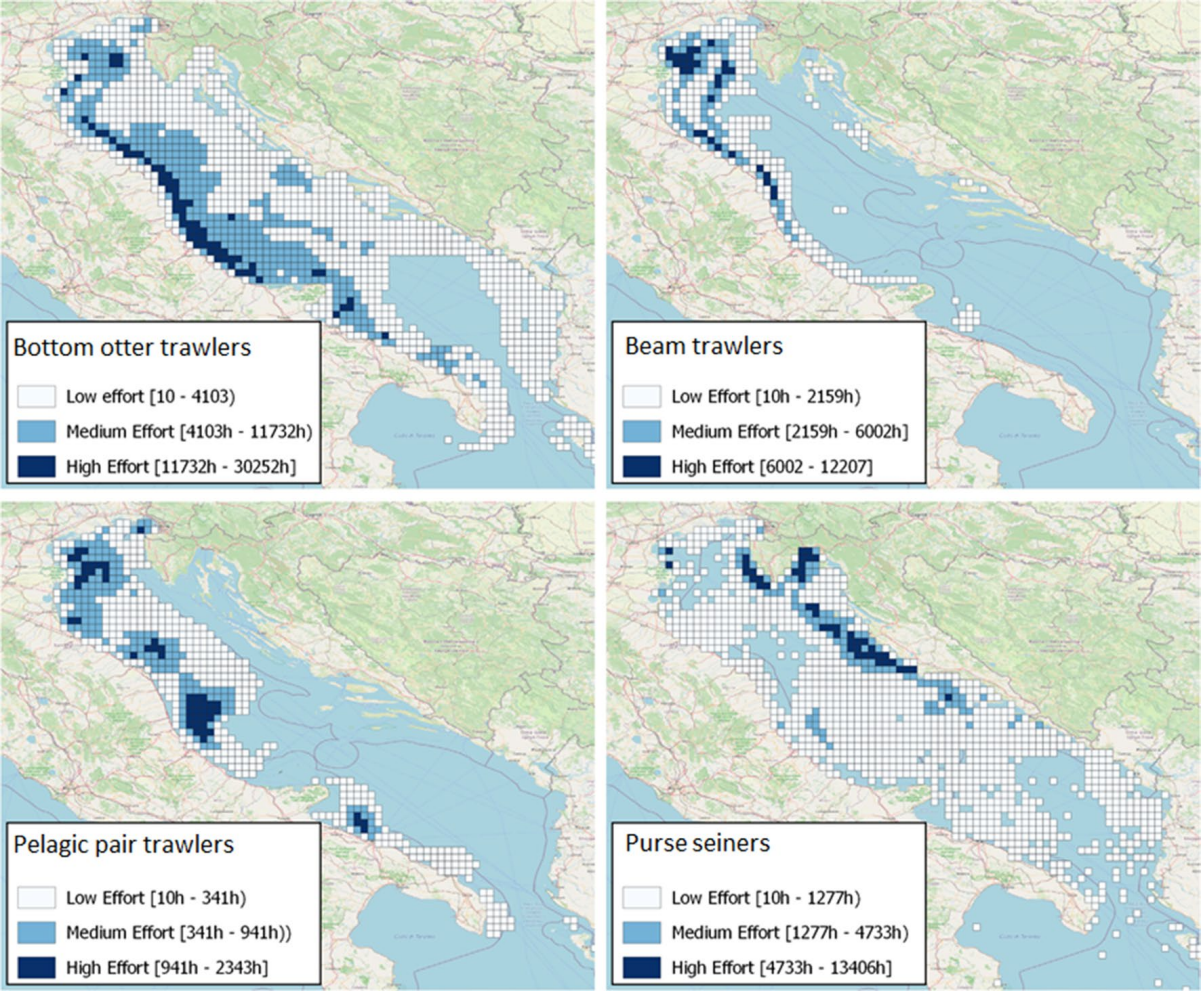
Spatial distributions, in the Adriatic Sea, of cumulative 2015–2020 fishing hours by estimated fishing gear at a 0.1$^{\circ }$ resolution. Maps were created with QGIS v.3.20.2 (www.qgis.org) using the OSM-Standard map of the QuickMapService plugin as the background map.

The GFW data of March–April 2020 and 2019 indicate that, in the Italian seas, fishing during the lockdowns involved a wider area and only $\sim$ 4% ($\sim$ 25 k) hours less than in 2019. In particular, the GFW data report $\sim$ 674 k fishing hours distributed over 316 cells at 0.5$^{\circ }$ resolution ($\sim$ 50 km) (the busiest cells are shown in Fig. 1a-left). From 1 March to 30 April 2020, GFW data report $\sim$ 649 k hours over 335 cells (the busiest cells are shown Fig. 1a-right). As regards the Adriatic Sea, the gear classification process indicates that each gear type exhibited a characteristic footprint (Fig. 2): the OTB exterted the most extensive and intense activity, with the effort peaking along the western Adriatic coast except in the area around the Po river mouth; the TBB showed a similar pattern, albeit with fewer hours, and was closely concentrated in the north-eastern Adriatic; the PTM was employed exclusively off the Italian coasts with four main activity areas, i.e., in the northeast, at the Po river mouth and off the coast of Abruzzo and northern Apulia; finally, the PS was distributed over the entire Adriatic, the activity being more intense off the eastern coasts. An *excellent* kappa agreement^31^ between AIS-estimated and EUFR-licensed gears over the five gear categories demonstrated the reliability of our classification algorithm. Additionally, EUFR data were well represented by the AIS data and vice versa, reflecting a reasonably good quality and consistency of the AIS data used in our analyses. Evaluation details are given in Sections 4.1-SI and 4.2-SI.

**Figure 3:**
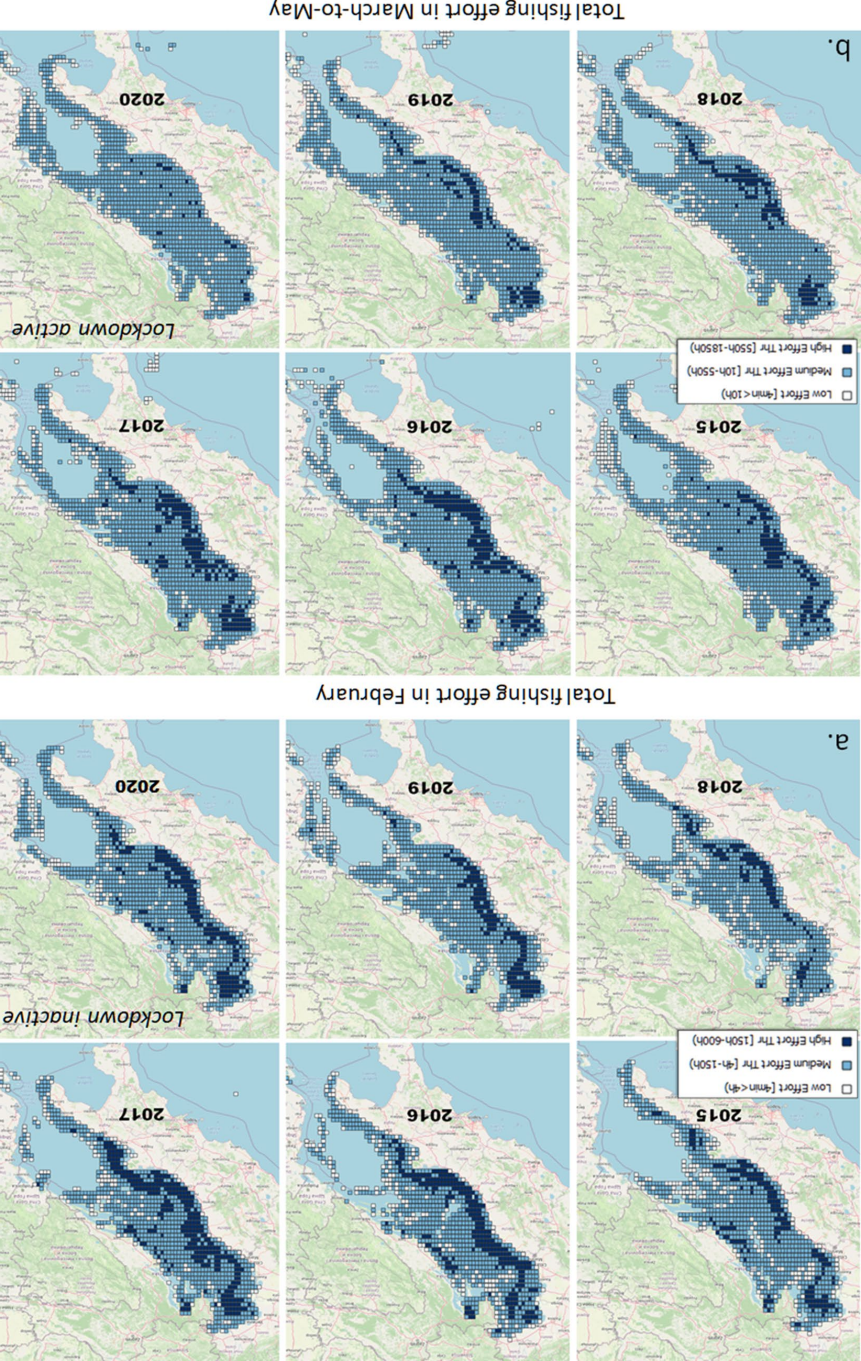
Comparison of the distributions of total fishing hours in the Adriatic Sea (0.1$^{\circ }$ resolution) in 2015–2020 in (**a**) February and (**b**) March-to-May (aggregated). The classification of the 2015 distribution is used for all years for consistency. Maps were created with QGIS v.3.20.2 (www.qgis.org) using the OSM-Standard map of the QuickMapService plugin as the background map.

As a result of the fishing cell processing of the GFW data, although we detected effort reduction in all Italian seas, in March–April 2020 some locations were still characterised by a high fishing effort with thousands of hours (Fig. 1 and Section 4.3.1-SI). This scenario was likely an effect of the pandemic. This pattern was confirmed by a higher resolution analysis of the AIS data for the Adriatic. Up to February 2020, fishing activity was equal to or higher than the previous years with a localised increase in the south-western Adriatic (Fig. 3 and Section 4.3.2-SI). The high-effort cells were much fewer in 2020, reflecting the lockdown effect in the March–May comparisons. The high-effort cells were consistent with those detected by the analysis of GFW data, but the higher resolution of the analysis indicates that these cells are actually more scattered. In a further analysis, we compared the summed distributions of annual and monthly fishing hours in the whole Adriatic and in the high-effort cells. The comparison highlighted an increasingly intense fishing activity up to February 2020, the effort being comparable to the level recorded in 2019 in the high-effort locations (Section 4.3.2-SI). The lockdowns reduced fishing activity proportionally both in the high-effort locations and in the whole Adriatic. The total number of fishing hours was overall lower in 2020 than in 2019 (− 4.7%). The monthly analysis identified a sharp reduction of fishing hours after the start of the March 2020 lockdowns (− 37%) and a general recovery in May 2020, although not to the levels of 2019. The monthly patterns also changed over the years due to regulations and decommissioning (Table 2 and Section 4.1-SI). In 2020, the patterns in the high-effort locations reflected those of the whole Adriatic, however the fisheries recovered faster in the most intensely exploited areas than in the whole Adriatic Sea. In the second half of 2020, the total number of fishing hours returned to the average levels of 2019, indicating that the restrictions affected fishing activities only in the lockdown period.

**Table 2:** Annual statistics of EU vessels registered to Adriatic ports according to the EU Fleet Register (EUFR): Total number of registered vessels; Newly constructed vessels; Vessels that moved their registration to an Adriatic port; Vessels that moved their registration outside the Adriatic; and Decommissioned vessels.

Year	EUFR vessel counts				
	Total	New	Entered	Exited	Decomm.
2015	1047	1	7	3	37
2016	1031	0	13	10	18
2017	1018	2	18	5	24
2018	932	1	4	2	87
2019	931	0	5	0	3
2020	926	0	4	2	6

**Figure 4:**
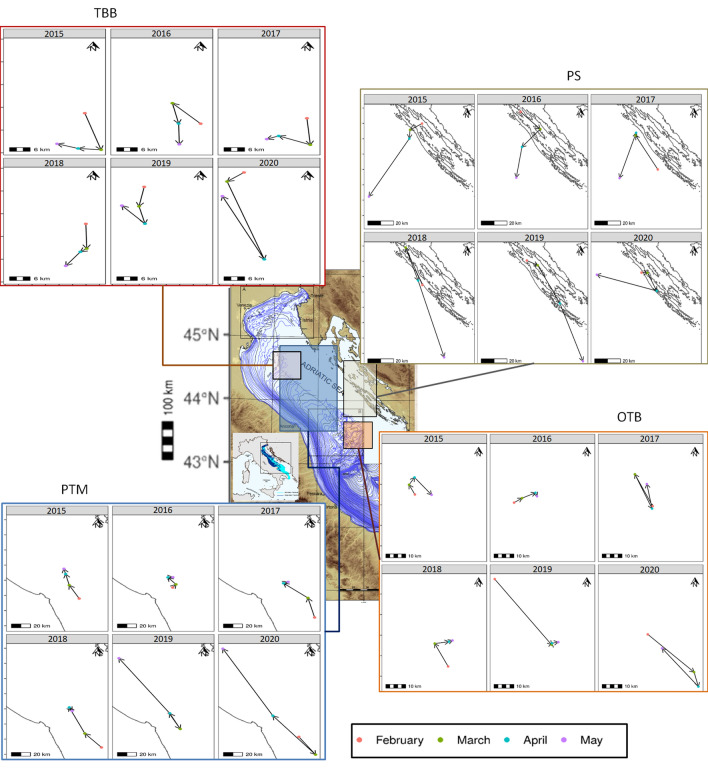
Distribution of the annual barycentre shifts over February and May between 2015 and 2020, reported per estimated fishing gear. The coloured bounding boxes depict the maximum extents of the barycentre movements. Acronyms refer to distinct fleets using purse seines (PS), pelagic pair trawls (PTM), beam trawls (TBB) or bottom otter trawls (OTB). The underlying bathymetry map belongs to a previous work^43^ of co-author F. Trincardi. Barycentre shift visualisation was realised with R v.4.1.2 (https://cran.r-project.org/bin/windows/base/) using the ggplot2 v.3.3.5, cowplot v.1.1.1, and raster v.3.5.2 packages.

We performed a spatio-temporal analysis to study the different fleets’ contributions to the overall fishing footprint, which demonstrated heterogeneous patterns. Indeed, PS fishery was little affected by the lockdown restrictions. The TBB fishery was strongly impacted by the EU lockdowns and made a slow recovery. The PTM fleet was moderately affected by the lockdowns and made a swift recovery in the whole Adriatic. The OTB fleet returned to pre-lockdown levels only at the end of 2020 and was strongly impacted by the lockdowns. Further details are given in Section 4.3.3-SI. The TBB, OTB, and PS barycentres had a focus area of $\sim$ 1$^{\circ }$ latitudinal range, whereas the latitudinal range of the PTM barycentre was $\sim$ 2$^{\circ }$ (Fig. 4). The 2020 lockdowns altered the TBB barycentre pattern and extended its range compared with the previous years. As regards the PS fleet, during the 2020 EU lockdowns it moved northward and returned to the south only at the end of the year, thus showing an inversion respect to 2017-2019 that was likely due to the pandemic restrictions. The barycentre of the PTM fleet moved in a more northward direction during the 2020 lockdowns than the previous years, with slightly longer monthly shifts reflecting a greater geographical range. Finally, the pattern of the OTB barycentre changed in March and April due to an increased activity range compared with the previous years and remained in this spatial range for the rest of 2020. Further details on barycenter patterns are given in Section 4.3.4-SI.

**Figure 5:**
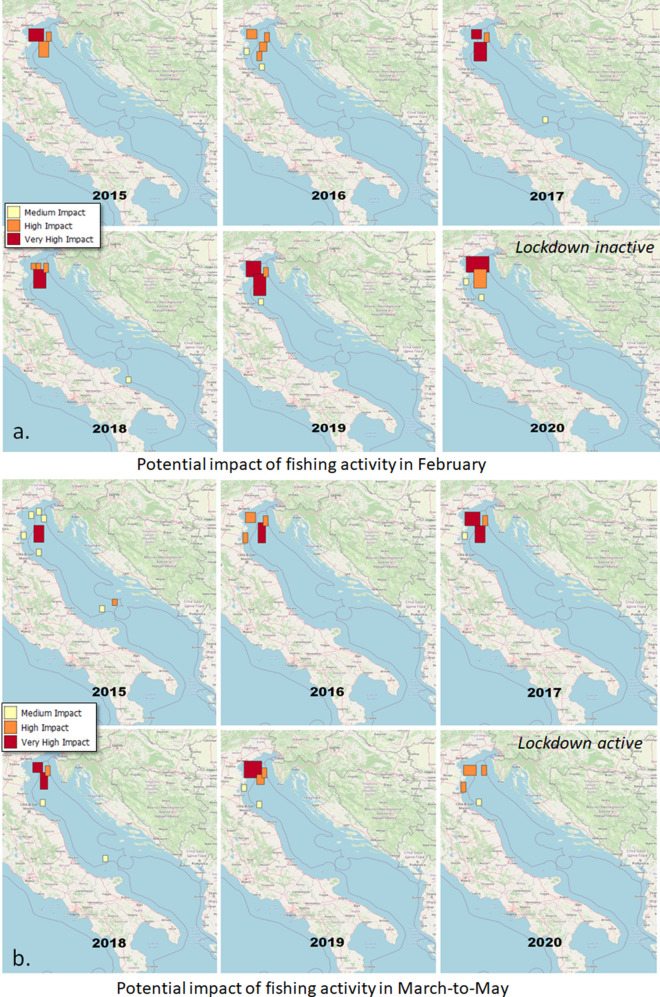
Spatial distribution of the total potential impact of fishing activities on endangered, threatened and protected (ETP) species in (**a**) February and (**b**) March-to-May (aggregated). Maps were created with QGIS v.3.20.2 (www.qgis.org) using the OSM-Standard map of the QuickMapService plugin as the background map.

In February and March–May 2020 the high-effort fishing areas and those inhabited by ETP species showed a strong overlap. An evident effect of the 2020 EU lockdowns, detected both in the GFW and the AIS datasets, was a scattering and overall reduction of the overlap during this period (Figs. 1b, 5). Analysis of the monthly trends demonstrated that the number of fishing hours at these locations during the lockdowns was generally lower than in the previous years, and in May 2020 did not reach the level of 2019. Although after the lockdowns the total fishing effort reverted to 2019 levels, the fishing hours at the locations at higher risk of impact remained below pre-lockdown levels throughout 2020, indicating a general attenuation of impact on ETP species in the Adriatic Sea during the pandemic. However, this was not true of all gear types. In particular, PS fishing hours increased at the locations at higher risk of impact up to February 2020 and remained constant, and well above 2019 values, both in March–May and in the second half of 2020. The PTM fleet strongly reduced its effort at the locations at higher risk of impact from February to March 2020 but then non-linearly increased this effort up to May 2020, which almost reached 2019 values and remained high also for the rest of the year. Further numerical details are given in Section 4.3.5-SI.

**Figure 6:**
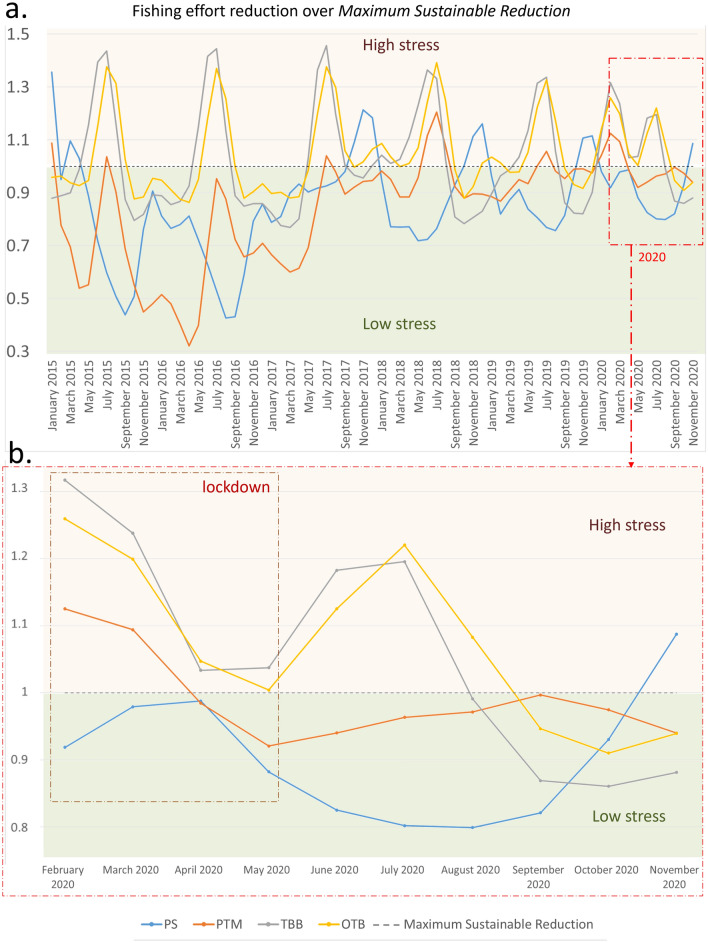
Fishing effort reduction divided by the maximum sustainable reduction reported for all fishing methods as estimated by AMSY: (**a**) values below the dashed line indicate low fishing hour limitation (low stress), those above the dashed line indicate strong limitation (high stress); (**b**) detailed representation of the February–November 2020 time series. Acronyms refer to the purse seine (PS), pelagic pair trawl (PTM), beam trawl (TBB) and bottom otter trawl (OTB) fleets.

**Figure 7:**
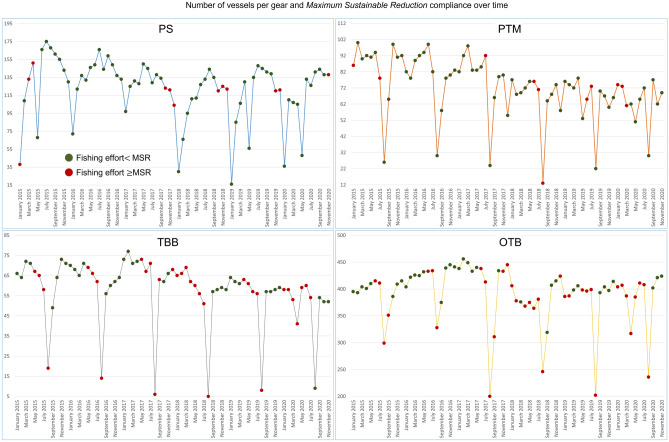
Number of fishing vessels per fleet over the months from January 2015 to November 2020, with indication of total fishing hours being greater or equal to (green dots) or under (red dots) the maximum sustainable reduction estimated for the fleet. Acronyms refer to the purse seine (PS), pelagic pair trawl (PTM), beam trawl (TBB) and bottom otter trawl (OTB) fleets.

Application of AMSY to the monthly fishing hours of the fleets (taken as an abundance index), produced several *r*-*kq* solutions (always above 5000) of the Schaefer dynamic for each fleet. This indicates that the fleet monthly effort time series fit well the Schaefer dynamics^10^, lending support to the hypothesis that fleet behaviour the can be assimilated to that of a fish stock. The fleets’ $Cmsr_t$ time series (Fig. 6a) highlight the strength of the monthly stress factors. For example, stock seasonality and fleet size reduction (e.g., due to vessel decommissioning in 2018) were those exerting the strongest influence. The patterns identified during the 2020 lockdowns (March–May) confirm that the pandemic acted as an additional stress factor whose consequences had never been observed before in those months of the year. A difference between the 2020 lockdown period and 2015-2016 is especially visible for the PTM and PS fleets. This difference is due to lower stress on these fleets in 2015-2016, principally because of lower restrictions, no market closure, and a generally better status of their target stocks with respect to more recent years^32^. One major finding was the synchronicity of PTM, OTB, and TBB fleet seasonality and the independent behaviour of the PS fleet. A detailed representation of the lockdown period and most of 2020 (Fig. 6b) confirms the indications of our spatio-temporal analysis: the PS fishery was not over-stressed by the lockdowns and was under-stressed during most of 2020; the PTM fleet was over-stressed in March 2020 but it rapidly reverted to an under-stressed situation in April, and remained so throughout 2020; the TBB and OTB fleets were the most over-stressed, since they remained over-stressed also after the lockdowns, and reached an under-stressed condition only at the end of 2020. The estimated growth rate *r* of each fleet confirms these observations (Table 3): the PS showed a 1.04 month$^{-1}$ growth rate, which indicates a very fast recovery from the temporary loss of fishing hours and $\sim$ 20 k maximum sustainable hours per month. The fast recovery of the PTM fishery was confirmed by a growth rate of 0.96 month$^{-1}$. Notably, this fishery should be allowed $\sim$ 4.5 k maximum sustainable hours per month. The slower recovery of the TBB and OTB fleets is reflected by their slower growth rates (respectively 0.57 and 0.56 month$^{-1}$), with the difference that the maximum sustainable hours of the former fleet were an order of magnitude less than the latter ($\sim$ 18 k vs. $\sim$ 117 k). For completeness of presentation, we also report, for each fleet, the overlap between the number of active fishing vessels per month, between 2015 and 2020, and their corresponding fishing effort relative to MSR (Fig. 7). This chart indicates which were the fleet sizes when the limitations were above or under the sustainable levels.

**Table 3:** Monthly growth rates and maximum sustainable hours per gear (and confidence intervals) estimated through AMSY.

	Growth rate ($month^{-1}$)	Confidence interval ($month^{-1}$)	Maximum sustainable fishing hours	Confidence interval (h)
PS	1.04	0.75–1.52	20,818	18,842–23,175
PTM	0.96	0.7–1.37	4526	4168–4977
TBB	0.57	0.37–0.95	18,150	14,602–22,901
OTB	0.56	0.37–0.88	117,033	94,725–148,086

Acronyms refer to distinct fleets using a purse seine (PS), a pelagic pair trawl (PTM), a beam trawl (TBB) or a bottom otter trawl (OTB).

## Discussion

In this paper, we have unfolded several aspects of the influence of the 2020 lockdowns on the Adriatic Sea fisheries, which were found to reflect the coarse-scale effects detected in all the Italian seas. Our findings indicate that the lockdowns slowed down a fast and generalised spread and increase of fishing activity that could severely affect the already heavily exploited Adriatic Sea stocks^32^. In May, at the end of the 2020 lockdowns, the overall fishing effort reverted to pre-lockdown levels, albeit not for all fleets. In fact, the purse seine fleet was not severely affected, apart from a change in the general barycentre pattern and the pelagic pair trawling fleet was moderately impacted and experienced slightly longer barycentre shifts than the previous years. The beam trawling and bottom otter trawling fleets were the most affected; indeed, the lockdowns strongly reduced their effort in a period that was already less productive and changed their fishing range extension and patterns. These fleets operate in specific areas and they did not adjust their strategies and patterns. Their effort recovered slowly and reverted to pre-lockdown levels only at the end of 2020. The 2020 lockdowns were generally beneficial for ETP species, because fishing activities at the locations where their density was higher decreased substantially in March and April but also throughout 2020. Only the purse seine fleet continued an intense activity at the locations at higher risk of impact. The overlap of pelagic pair trawlers with ETP species shrank briefly and returned to pre-lockdown levels in May. The risk of impact from beam trawlers and bottom otter trawlers showed a considerable reduction. This finding predicts a potential population size increment of ETP species, to be checked against the next abundance surveys when available.

The intense purse seiner activity during the 2020 lockdowns and its unchanged risk of impact on ETP species were unique among the fleets analysed. One possible explanation is the diversification of the markets and of the final consumers of the Croatian purse seine fleet, which supplies the canning and saltfish industry and the tuna farms^33^. Notably, the bluefin tuna farming industry has been flourishing in the Croatian islands since 2000^34^ and its production volume doubled between 2014 and 2018^35^. In this industry, part of the feed consists of local small pelagic fish^36^, whose demand was probably stable also during the lockdowns. As regards vessel presence in the Adriatic, the slight reduction of registered EU vessels in 2020 with respect to 2019 (− 0.2%, from 931 to 926 ships, Table 2) was not an effect of the pandemic. Indeed, the EU vessels recorded in the AIS dataset had already been declining for some years, after rising from 2015 to 2016. The rise was due to the reduction to 15 m of the length of the vessels required to mount an AIS transponder, which involved a peak in 2016. However, in more recent years several vessels have either moved outside of the Adriatic or have been decommissioned (with a peak in 2018).

Our data indicate that the 2020 lockdowns occurred during a seasonal fishing effort reduction involving all fleets except the purse seiners. The charts illustrating the relative effort reduction (Fig. 6a,b) indicate that common regulations have generally had a much lower impact on the fishing effort than fleet seasonality and mass decommissioning. The lockdown was a harsher stress factor than the harvesting control rules and made the different fleet responses emerge in a clearer way. One major finding of the study is that bottom otter trawling (the most intense fishing method) took twice the time to recover compared with pelagic pair trawling. This should be considered when introducing management policies, to avoid excessive economic loss, especially given the large catch volumes of bottom otter trawlers. Other stress factors that influenced fleet responses were fishing seasonality and the maximum sustainable effort reduction. These are major factors that should carefully be considered if excessive fleet stress is to be avoided and management policies are to grant economic welfare. They derive from the finding that the fishing effort time series fitted the Schaefer stock dynamic equation (i.e., they generated thousands of solutions). Thus, the fishing effort can be used as an abundance index, i.e. each fleet can reasonably be modelled as a stock subject to a complex combination of stress factors that reduced its abundance (i.e., its fishing hours). The emergence of traits like intrinsic growth rate (*r*) indicates inertia in recovering fishing hours from one month to the next, due to the fact that the effects of the stress factors spanned several months. These effects include (i) the progressive relaxation of restrictions, (ii) the long rebuilding time and seasonal availability of target stocks, and (iii) the entry-exit scheme of fishing vessels. A fleet’s intrinsic growth rate summarises its capacity and effectiveness to adapt to these effects, which also depend on the fleet size, whereas the *kq* trait summarises the capacity of the resources exploited by the fleet to sustain its fishing activity and estimates a maximum sustainable number of fishing hours for each fleet; this information has the potential to be directly used in management strategies. The *kq* trait principally correlates with target stock availability but also embeds the capacity of the supply chain to manage the catch. If the supply chain were unable to receive and sell the catch, the fishing activity would need to be reduced. This effect is typically negligible in the current greedy global economic system, but the pandemic has magnified its importance. It is also worth noting that a side effect of inadequate supply chain capacity is a higher level of food waste^37^. Thus, balancing supply and demand in the seafood chain by including market constrains into fishery management would also enhance environmental sustainability.

The pandemic has highlighted the very low resilience of the seafood system to market closure and its differentiated impact across countries and social levels^38^. The lockdowns have affected both the supply chain (via market closure) and vessel activity (via the sanitary restrictions and the forced stop). Since these aspects are correlated, they have generated a vicious circle that has acted as a complex additional stress factor on the Adriatic fleets. The *r* and *kq* traits calculated in the present study account for reconfiguration of the supply chain change and the fishery response to it in an implicit manner, as they summarise a fleet’s response to all the stress factors occurring in a specific area and fishing context. A comprehensive management strategy would need to measure the efficiency and response of a supply chain to the loss of fishing hours and resources in an explicit way and could integrate this information with our findings^39–42^.

In summary, the COVID-19 pandemic has offered the opportunity to observe and quantify some key intrinsic traits of Adriatic Sea fisheries. Our findings, have the potential to help decision-makers produce monthly harvesting control rules to (i) cut the number of fishing hours per fleet (which should be well below the maximum sustainable reduction); (ii) take into consideration fleet resilience by imposing stronger restrictions on fast-recovering fleets (pelagic pair trawlers and purse seiners) and less harsh restrictions on slow-recovering fleets (bottom otter and beam trawlers), and (iii) optimise harvesting control rules based on fishing seasonality, fleet size, and fishery volumes. These control rules should also take into account the spatial overlap between areas of intense fishing activity and the geographic distribution of ETP species if they are to enhance ecosystem sustainability. Harvesting control rules conceived in this way would go a considerable way towards fishery sustainability viewed in terms of ecosystem protection, control of the economic pressure, stock rebuilding, lower food waste and sustained resource availability.

The presented approach can be used for other marine areas and fleets beyond the Adriatic, e.g., to all Mediterranean Sea fleets, because the type of analysis presented is mostly independent of the specific application case. For example, the AMSY model can process new fleet data to (i) verify that these follow Schaefer dynamics, (ii) estimate *r* and *kq* traits and (iii) produce helpful information to management organisations. The proposed machine learning model for gear classification is flexible enough to be used with other AIS data and areas. The type of spatial analysis described can be directly re-used with other areas to extract spatio-temporal trends and patterns and evaluate the overlap between fishing activity and ETP species presence. It is also general enough to process the activity and gear classifications estimated by other algorithms, e.g., the GFW global scale data, and quickly produce estimations for new areas.

## Supplementary Information

Supplementary Information.

## Data Availability

Source code and aggregated vessel information are are openly accessible (Supplementary Information).
