# Supplementary material for: COVID-19 lockdowns reveal the resilience of Adriatic Sea fisheries to forced fishing effort reduction
Source: Sci Rep. 2022 Jan 20;12:1052. doi: 10.1038/s41598-022-05142-w (PMC8776884; doi:10.1038/s41598-022-05142-w)
Supplement: Supplementary file 1 — Supplementary Information. [file 41598_2022_5142_MOESM1_ESM.pdf]

COVID-19 lockdowns reveal the resilience of Adriatic Sea fisheries to forced fishing effort reduction - Supplementary Information

Gianpaolo Coro[1,*], Anna Nora Tassetti[2], Enrico Nicola Armelloni[2,3], Jacopo Pulcinella[2], Carmen Ferrà[2], Mario Sprovieri[4], Fabio Trincardi[5], and Giuseppe Scarcella[2]

[1]Institute of Information Science and Technologies (ISTI), National Research Council of Italy (CNR), Pisa, 56124, Italy
[2]Institute for Biological Resources and Marine Biotechnology (IRBIM), National Research Council of Italy (CNR), 60125, Ancona, 60125, Italy
[3]University of Bologna, Department of Biological, Geological and Environmental Sciences, Bologna, 40126, Italy
[4]Institute of Anthropic Impacts and Sustainability in the Marine Environment (IAS), National Research Council of Italy (CNR), Torretta Granitola, 91021, Italy
[5]Department of Earth System Science and Environmental Technologies, National Research Council of Italy (CNR), Rome, 00185, Italy
[*]gianpaolo.coro@cnr.it

ABSTRACT

This document complements the information reported in the main manuscript. It is organised as follows: Section 1 reports background information on fishing activity monitoring based on vessel tracking data. It also describes the fisheries of the Adriatic Sea and the currently active management strategies, with a mention of COVID-19 restrictions in the Adriatic Sea countries. Section 2 contains the descriptions of the primary data sources used for the experiments described in the main document. Section 3 reports the algorithmic details of our analyses. Section 4 provides numerical details to support the results reported in the main document. Section 5 reports information about the project in which our study was conducted. Finally, Sections S1-S4 report additional charts, per gear, of monthly fishing effort (S1), barycentre shifts (S2), annual (S3) and monthly (S4) effort in locations with medium and high concentrations of endangered, threatened and protected (ETP) species, and fleet-assessment analysis charts (S5).

Glossary

OTB = Bottom otter trawl
PTM = Pelagic pair trawl
TBB = Beam trawl
PS = Purse seine
MSY = Maximum sustainable yield
r = intrinsic rate of growth
kq = carrying capacity multiplied by catchability
ETP species = endangered, threatened and protected (ETP) species

Source Code

The source code and aggregated vessel information are available on Zenodo at the following link:
https://zenodo.org/record/4761890

1 Background information

1.1 Monitoring Fishing Activity through Vessel Tracking Data

Valuable information about fishing patterns and vessel group behaviour can be extracted from vessel position data and logbook analysis[1–6]. In most European countries, vessel data collection and collation has been systematised since 2006 through

Vessel Monitoring Systems (VMSs). A VMS is an electronic, computer or physical system that enables monitoring bodies to control fishing activity through the systematic collection of navigation data. However, VMSs do not provide information on the activities of vessels from other countries. For example, the Adriatic Sea and its resources are shared by several States, which cannot monitor each other's activities. Furthermore, the coarse temporal resolution of most VMS data (in Italy, about 2 hours[7]) is insufficient to study vessel activities of short duration and fast group dynamics[8–11]. In the past decade, VMS data have been collected by terrestrial networks and satellite systems across the world and have become available especially for scientific purposes, e.g., to identify fishing patterns[12], estimate fishing pressure[13,14], assess the impact of trawling on seafloor[15], and devise ecosystem approaches to resource planning[16–20]. In this context, vessel data (often anonymised or aggregated) are distributed with attached information on fishing activity estimated automatically[21,22]. Since May 2014, Automatic Identification Systems (AISs) have become compulsory on board EU fishing vessels at least 15 m in length overall (LOA). AIS big data flows are characterised by high generation velocity, large volume, high noise, missing data and considerable representational heterogeneity, which require suitable models and e-Infrastructures for storage, management and processing[23,24]. Early information extraction methods used speed and direction information to distinguish between fishing and non-fishing locations and filter out noisy data[21,25]. Subsequent advances allowed applying statistical analysis to the speed distribution of specific fishing techniques and gear types[14,26,27]. Such gear-specific models usually represent tracks as 1st-order Markov processes. Although they lack generalisation, they can effectively classify the activity of target fleets[11,28]. More recently, deep learning and data mining techniques have been providing more accurate cross-fleet fishing activity classification[22] and gear identification[29–31]; however, high noise level and data incompleteness make these methods better suited to large-scale analyses, to compensate for misclassification bias.

Analyses of vessel data have allowed fleet behaviour to emerge from fishing patterns, highlighting its resemblance to the behaviour of apex predators[32]. In fact, fleet activity can strongly reduce the abundance of a commercial species living in an area (i.e., a stock) in a short time and affect its productivity over a long time. The lack of biological regulating factors and the constant pursuit of reckless profit make fleets highly dangerous predators. Fishing effort limitations and prompt management measures are crucial to balance their greed and avoid stock depletion. Furthermore, fishers can effectively hinder compliance with unpopular regulations and exert a direct influence on fishing patterns[33–35]. Some ecosystem models implicitly include fisher responses to regulations in fleet dynamics modelling, assuming that their goal is to maximise profit while minimising effort[32]. These models account (either implicitly or explicitly) for all the driving forces acting in the ecosystem, including interactions with fish markets, resource availability and fisher responses[36,37]. The response of a fishery to all these forces can be summarised as its *resilience*, i.e., its capacity to overcome temporary difficulties and return to earlier profit levels. However, resilience has never been quantified.

1.2 Fishery Management in the Adriatic Sea

The countries that have coasts on the Adriatic Sea are Albania, Bosnia and Herzegovina, Croatia, Italy, Montenegro and Slovenia. The target stocks of the Adriatic Sea fleets (pelagic pair trawlers, purse seiners, bottom otter trawlers and beam/*Rapido*[38] trawlers) include the European hake (*Merluccius merluccius*), the common sole (*Solea solea*), the mantis shrimp (*Squilla mantis*), the red mullet (*Mullus barbatus*), the common cuttlefish (*Sepia officinalis*), the European anchovy (*Engraulis encrasicolus*), the European pilchard (*Sardina pilchardus*), and the deepwater rose shrimp (*Parapenaeus longirostris*). These species alone accounted for nearly 70% (98,600 tonnes over 143,900 tonnes) of the total catch in the basin in 2019[39]. Thus, they are the targets of beam (common sole, mantis shrimp, common cuttlefish), bottom (red mullet, deepwater rose shrimp, European hake), and pelagic-pair (European anchovy and pilchard) trawlers and purse seine vessels (European anchovy and pilchard). The Adriatic demersal stocks usually do not perform wide migrations and do not change location within the year. However, distinct distribution patterns can be observed between juvenile and adult populations[40]. Instead, as demonstrated by acoustic surveys, the distribution of small pelagic stocks can largely change over the years but always falls in the most fished areas[41]. Generally, Adriatic stocks are subject to fishing activity in all months of the year.

In the Adriatic, as in the rest of the Mediterranean Sea, the fishing effort is monitored and regulated, by fisheries management organisations, in terms of fishing hours rather than total catch. This approach complicates the work of scientific advisors, who are required to calculate fishing effort thresholds in a variety of fisheries that are characterised by very different target fish stocks and productivity levels[42]. The problem is compounded by the fact that in the Adriatic Sea most stocks are targeted by fleets from different countries, whose fishing effort also needs to be considered[43]. However, scientists can rely on an extended series of historical fishery data and are helped by the relative isolation of the Adriatic Sea from the rest of the Mediterranean[44,45].

The General Fisheries Commission for the Mediterranean (GFCM) – the main intergovernmental fisheries management organisation in the Adriatic – has been regulating fishery activities through multi-annual plans. For small pelagic fisheries, especially those targeting sardine and anchovy, the management plan[46] (and its subsequent updates) involves management measures and harvesting control rules. Its aims are to maximise the long-term yield of small pelagic fisheries while minimising the risk of stock collapse. The main strategies include (i) reducing the total allowable fishing effort, especially by limiting

fishing days for purse seiners and pelagic pair trawlers, and (ii) applying temporary spatial closures to protect nursery and spawning areas. Demersal stocks are managed by a separate and more recent multi-annual plan[47], whose overarching goal is to reach by 2026 the maximum sustainable yield (MSY), i.e., the maximum catch of a fish stock that can be taken indefinitely without depleting the population, for the target species. The plan is mainly aimed at high-effort fleets and involves two phases. The objective of the first phase is to progressively reach a fixed percentage effort reduction for each fleet by 2021 (e.g., -12% for otter trawlers and -16% for beam trawlers), with respect to the average 2015-2018 effort. The second phase (2022–2026) envisages setting yearly effort quotas for each fleet depending on the status of its target stocks. The plan will also regulate fleet size and set up restricted areas to maintain a sustainable biomass. Altogether, the GFCM plans ensure that the fishing effort is commensurate to stock abundance. Their success therefore requires accurate inputs from scientists. The COVID-19 pandemic has involved restrictions for all world fisheries to stem the spread of the infection. In Italy, restrictions were imposed in early March in the North-East, but by March 12 a national lockdown was in place for all commercial activities that involved retailing, catering and personal services[48]. On March 23, all working activities except essential services were suspended until May 18[49]. In the eastern Adriatic, non-essential services and commercial activities were shut down from March 16 in Slovenia[50] and from March 19 in Croatia[51]. As a result, the demand for seafood collapsed, prices plummeted, and fish markets closed, causing reduced fishing activities (see also Table 1 in the main document). Moreover, fishing activities were directly restricted by the practical difficulties of applying social distancing and safety protocols on board. Several operators suspended their activities altogether throughout the lockdown period.

2 Data sources

2.1 Data for coarse-scale pattern analysis

The Google's Global Fishing Watch[52] (GFW) is a Web portal managed by Google in partnership with Oceana and SkyTruth. The GFW produces a global view of commercial fishing activities by analysing VMS, AIS and Infrared Imaging Radiometer Suite (VIIRS) data. Its global scale data tables report vessel activity information aggregated at 0.01° and 0.1° resolutions that can be accessed for scientific purposes. The published datasets report fishing activity cells classified by machine learning models[22], which identify fishing activity locations mainly based on speed information. Although the GFW data are well suited to large-scale analyses and averages (e.g., at 0.5° resolution), their large amount and heterogeneity make them less accurate for smaller-scale analyses[53].

The GFW data of March-April 2020 and 2019 have consistently been used in ecological and economic analyses[54–56]. We therefore did not question their quality, also because we employed them for a coarse resolution pattern recognition that reduced data noise and uncertainty biases.

2.2 Data for Adriatic-scale pattern analysis

The Astra Paging data collector[57] was our primary source to conduct a detailed analysis of fishing activity in the Adriatic Sea. Astra Paging hosts one of the largest worldwide AIS databases, which goes back to 2009. In particular, we used the complete census of fishing vessel positions, sampled at 5-minute intervals, collected by terrestrial AISs in the Adriatic Sea. The relevant records were those falling within GFCM geographical sub-areas (GSAs) 17 and 18[58]. AIS data between 2015 and 2020 were used to normalise fleet size and reduce the bias due to the massive increase in AIS data recorded after May 2014, when the minimum length of AIS-equipped EU vessels was reduced to 15 m. AIS records report information every few seconds on vessel position, speed over the ground, course and rate of turn. They also include trip-related information (e.g., destination and arrival time) and identifiers such as call sign (IRCS), vessel type, name, Maritime Mobile Service Identity (MMSI) and International Maritime Organisation (IMO) number.

2.3 Data for AIS data quality evaluation

We used the data of the EU Fleet Register[59] (EUFR), a public database containing the data of the fishing vessels of all EU member States, to make a quantitative evaluation of AIS data quality. The EUFR contains vessel administrative information (name, registration port), technical specifications (lengths, fishing gear) and relevant events (construction, modifications, decommissioning). These data allowed estimating the size of EU Adriatic fleets over time and to compare it to the one estimated from the AIS dataset. This operation allowed validating AIS data completeness and enabled their reasonable use in our experiment.

3 Algorithms

3.1 AIS data quality control

We employed vessel registration port to estimate the approximate fleet size around a port. Such approximation can be tolerated when validating big data such as the AIS data we used[60]. The EUFR data were filtered by retrieving only vessels of at least 15

m LOA registered in the Italian, Slovenian and Croatian ports of GSAs 17 and 18. In the validation process, we linked AIS data to EUFR events by matching the identifiers stored in both datasets, i.e., IRCS, MMSI, vessel names and IMO number. This heuristic process sought an exact match of one identifier at a time (primary identifier), using another identifier as a secondary control. The match between two records was considered successful if the primary identifiers matched exactly and the minimum edit distance between the secondary identifiers was < 3 (high similarity). Unsuccessful matching for all combinations of primary and secondary identifiers prevented identification (unidentified vessel). This process enriched the AIS data with information on gear licence, flag country, registration site, LOA and relevant events. The final validation of AIS data completeness against EUFR data was obtained by introducing three metrics to estimate the annual overlap between the number of operational EU Adriatic vessels recorded in each dataset. To do this, for each year from 2015 to 2020 we calculated the number of EUFR vessels that were equipped with an AIS transponder (*expected* vessels) and the number of EU vessels that broadcast AIS data (*observed* vessels). Finally, we defined the following metrics:

$$Expected\, coverage_{year} = \frac{expected\, vessels}{Total\, EUFR\, vessels}$$

$$Observed\, coverage_{year} = \frac{observed\, vessels}{Total\, EUFR\, vessels}$$

$$Representativeness\, score_{year} = \frac{observed\, vessels}{expected\, vessels}$$

These scores measure the amount of complementary information found in the EUFR and AIS data, hence the extent to which the AIS data represent all the EU vessels registered in the Adriatic Sea.

3.2 Gear classification

The fishing gear types used by the AIS-equipped vessels were automatically inferred using a revised version of the workflow published by Galdelli et al.[61]. The source code is included in the Zenodo repository indicated at the beginning of this document. Our workflow estimates the gear used by a vessel in a fishing trip. It uses speed and position reference clusters, previously learned through cluster analysis from historical annotated data of the Adriatic Sea, for each gear. It identifies the gear that a vessel used in a trip among five types: bottom otter trawl (OTB), pelagic pair trawl (PTM), beam trawl (TBB), purse seine (PS) and other gear (OTHER). As a first step, it estimates which gear types are consistent with each trip based on the overlap between the speed and position distributions and the reference clusters. As a second step, the most probable gear type is assigned to the vessel's trip by a Random Forest classifier[62] based on (i) the frequency of potential gear types the vessel has used the same month and (ii) the number of trips it made that month. The analysis is based on monthly data because fishing strategies change depending on the season. As a final step, the workflow labels all trajectory points in a trip as fishing/non-fishing activity points and calculates the fishing time for each point. To do this, it sets a speed threshold for each trip to estimate when the gear was/was not deployed, i.e., when the vessel was/was not fishing, and the trajectory intervals associated with fishing. The threshold is calculated by detecting the point speeds that are entirely encompassed in the gear's speed cluster. The process also detects non-fishing trips and excludes them from further analysis. Finally, fishing time per point is calculated. For the towed gear types (OTB, PTM, TBB) it is computed as the time difference between two consecutive fishing points; for PS – where gear identification is less accurate due to its complex spatial patterns – it is approximated by dividing the total fishing time by the number of fishing points.

We applied this workflow to the 2015-2020 AIS dataset and assessed its classification performance against EUFR-Adriatic data. Since the classification confidence of our workflow was higher for longer trips with uniform sampling[61], we processed only trips with at least 12 transmissions and a percentage of data gaps not exceeding 90% of trip duration. The classification performance was estimated according to a confusion matrix that reported the overlap between estimated vessel gear and the corresponding gear reported in the vessel's EUFR licence. Considering the EUFR gear as the ground truth, we calculated the following evaluation parameters:

For each gear type g:

calculate *true positives* as the number of AIS-estimated g trips corresponding to EUFR g types;

calculate *false positives* as the number of AIS-estimated g trips not corresponding to EUFR g types;

calculate *true negatives* as the number of AIS-estimated non-g trips that correspond to EUFR non-g types;

calculate *false negatives* as the number of AIS-estimated non-g trips that correspond to EUFR g types.

Based on these parameters, classification performance was assessed according to the following metrics:

$$Precision = \frac{true\,positives}{true\,positives + false\,positives}$$

$$Sensitivity = \frac{true\,positives}{true\,positives + false\,negatives}$$

$$Specificity = \frac{true\,negatives}{true\,negative + false\,positives}$$

Overall agreement with respect to chance was measured using Cohen's Kappa[63].

3.3 Spatio-temporal analysis of fishing effort

We implemented an algorithm to classify the fishing effort cell-by-cell, at 0.5° and 0.1° resolutions. The process is based on the assumption – supported by empirical observation of the data at hand – that the distribution of fishing hours over the cells of a large area is approximately log-normal. Indeed, in this distribution, cells characterised by a higher number of hours tend to be farther from the geometric mean than those with fewer hours. Fishing activity per cell was therefore classified based on the confidence limits assuming a log-normal distribution of fishing hours, through the following rules:

For each cell in the area:

total fishing hours above the upper confidence limit → *high-effort cell*

total fishing hours between the lower and upper confidence limits → *medium-effort cell*

total fishing hours under the lower confidence limit → *low-effort cell*

This classification allowed studying the spatial distribution of high-effort locations, to discover high-intensity patterns in the Italian seas and to compare these patterns with the trends highlighted in the Adriatic Sea. The analysis was conducted on both GFW (at 0.5° resolution) and AIS data (at 0.1° resolution). In the GFW dataset, it highlighted the difference between the fishing effort identified in the Italian seas in March-April 2019 and in March-April 2020. In the AIS dataset, it yielded patterns of fishing activity change in the Adriatic Sea over the years and months, from 2015 to 2020. In particular, annual aggregations were used to highlight the overall change of fishing activity in 2020, due to the pandemic, compared with the previous years. This change was further explored using monthly aggregations, which were also used to estimate the recovery speed of the different fleets in relation to the stress factors and to understand the response of each fleet to the pandemic.

3.4 Barycentre calculation

Barycentre calculation of each gear over time was performed to explore the fishing activity patterns in the Adriatic Sea. The barycentre is the mean location of the observed fishing effort, thus it is not necessarily a high fishing effort location. We weighted each 0.1° cell by the number of fishing hours to calculate the coordinates of the barycentre:

$$Barycentre\,Latitude_t = \frac{\sum_{i=1}^{n} latitude_{i,t} \cdot fishing\,effort_{i,t}}{\sum_{i=1}^{n} fishing\,effort_{i,t}}$$

$$Barycentre\,Longitude_t = \frac{\sum_{i=1}^{n} longitude_{i,t} \cdot fishing\,effort_{i,t}}{\sum_{i=1}^{n} fishing\,effort_{i,t}}$$

where t is the unit of the time aggregation (month of the year) and n is the number of fishing cells at time t.

The sequence of fishing effort barycentres over months of a given fleet is a spatial time series that allows studying fishing vessel movements in the various seasons[6]. In our experiment, this sequence also allowed assessing how the pandemic altered fishing activities in 2020 compared with the previous years.

3.5 Potential impact on endangered, threatened and protected species

The presence of ETP species was established by extracting IUCN species observation records from the Ocean Biodiversity Information System[64] (OBIS), which has been demonstrated to be suitable for this type of large-scale biodiversity analyses[65]. The possible observation sampling bias was managed through a statistical analysis of the density of ETP species. In particular, the fishing cells were categorised as low-, medium- or high-impact assuming a log-normal distribution of the number of ETP species per cell, through the following rules:

For each cell in the area:

number of ETP species in the cell above the upper confidence limit → *potential high-impact cell*

number of ETP species in the cell between the lower and upper confidence limits → *potential medium-impact cell*

number of ETP species in the cell under the lower confidence limit → *potential low-impact cell*

We conducted this analysis in the Italian seas at 0.5° resolution using the GFW dataset, and in the Adriatic Sea at 0.1° resolution using AIS dataset. In the Adriatic Sea, we performed further spatial aggregation of cells by density-based clustering (with DBScan[66]). This step generated larger aggregates of impact areas, whose composition over time highlighted more clearly the pandemic-related change in the potential impact of fishing activities.

3.6 Fleet assessment model

An approximate relationship[67] between MSY, r, and k is $MSY \approx k \cdot r/4$. Thus, knowing r and k allows estimating MSY. The Schaefer model[68] correlates stock biomass dynamics (B_t) with catch (C_t) by the following analytical formula:

$$B_{t+1} = B_t + r \cdot (1 - B_t \cdot k) \cdot B_t - C_t$$

The idea behind data-limited stock assessment models is that, if the catch is known and r and k can be estimated with acceptable approximation, it is possible to calculate the biomass evolution in time. Catch Maximum Sustainable Yield[67] (CMSY), one of the most widely used data-limited methods, can accurately reproduce the biomass time series based on a Monte Carlo approach, which generates viable r-k pairs from the Schaefer formula. CMSY estimates prior ranges of r-k pairs from prior qualitative information on the general resilience of the species to fishing mortality. An alternative version of CMSY, the Abundance Maximum Sustainable Yield[69] (AMSY), uses a rewrite of the Schaefer formula with an abundance index A_t instead of biomass B_t:

$$Cq_t = A_t + r \cdot (1 - \frac{A_t}{kq}) \cdot A_t - A_{t+1}$$

A_t can provide a fishery indicator like the catch per unit of effort or another index of species abundance. AMSY reconstructs the catch time series C_t multiplied by *catchability* q, a coefficient that reflects fishery efficiency, i.e., how much biomass becomes catch. AMSY also estimates MSY, r and kq (the carrying capacity multiplied by catchability). If A_t is a monthly time series (i.e., t is a monthly index), then r is its monthly growth and kq is the stock abundance in the absence of fishing pressure. The main underlying hypotheses of AMSY are that (i) the abundance index A_t, the catch Cq_t, r and kq are correlated through Schaefer dynamics and (ii) q, r and kq do not change over time in the stock's area, although they may be different for the same species in different areas.

4 Additional numerical analysis of the results

4.1 Representation of AIS data in EUFR data

In the Adriatic Sea EUFR data identified an ∼11% reduction of registered vessels of more than 15 m LOA between 2015 and 2020 (Table 2 in the main document). The reduction was largest between 2017 and 2018 (from 1018 to 932 vessels) and was mainly due to the EU fleet capacity limitation plan[70], which set caps on kilowatts and gross tonnage for each EU member State and allowed new vessels to operate in a given area only after their equivalent capacity had been removed (e.g., by decommissioning). The measure exerted its maximum effect in 2018, when 87 vessels were decommissioned and 2 vessels deregistered from the Adriatic ports. Another contributing factor was the limited number of new vessels entering the EU Adriatic fleets (Table 2-*New* and *Entered* columns), since 20 new vessels (2 newly constructed and 18 registered to an EU port) were entered in 2017, but only 5 were entered in 2018. Notably, the contribution of new constructions to the Adriatic fleets

was always low, with only 3 vessels built in 6 years. A comparison of AIS and EUFR data disclosed that 1114 out of 1169 vessels (95.3%) included in the AIS dataset belonged to EU Member States and 55 (4.7%) to non-EU States (30 were Albanian). The AIS dataset contained a total number of 10 million vessel trajectory points (of which 98% belonged to European vessels) corresponding to 2 million estimated fishing hours. The change in the *expected coverage* over the years was low (1%, ranging between 78 and 79%, Table SI-1), indicating that the relative number of EUFR vessels carrying an AIS transponder was almost constant, also during the pandemic. In contrast, the number of vessels that broadcast AIS data was more variable, i.e., the *observed coverage* showed a greater variability (5%, from 74% in 2016 to 69% in 2020), with a slowly decreasing trend. The phenomenon also affected the annual *representativeness score*, which fell from 93% in 2016 to 89% in 2020, although there was no reduction in 2020 compared with 2019. Overall, these metrics indicate that the EUFR data are well represented by the AIS data and vice versa, reflecting a reasonably good quality and consistency of the AIS data used in our analyses.

4.2 Gear classification algorithm reliability

To summarise our gear classification process, we drew a representation of the total spatial extension and effort of each fleet in the Adriatic Sea. The effort of EU and non-EU vessels was aggregated in 0.1° cells and summed over 2015-2020 (Figure 2 in the main document). A year by year summary of the AIS vessels recorded on these maps is reported in Table SI-2, which also shows their distribution according to the gear categories. The vessels that were estimated to use at least two gear types in the same year are counted in the *mixed* category. Vessel and trip numbers decreased over time, especially in 2018 after the decommissioning of EU vessels. The estimated vessel distribution in the gear categories varied little over time. OTB was the richest category with an almost stable average number of vessels (∼389), followed by PS (∼97 average) and TBB (∼45 average); PTM was the category with the smallest number of vessels (∼26 average). Trips with OTHER gear types were the least numerous (∼8 average) and mostly used gillnets and longlines. These gear types showed a steep increase of 20 vessels in 2016 and an average increase of 5 vessels after 2017. To assess the reliability of this classification, we calculated a confusion matrix between AIS-estimated and EUFR-licensed gears (Table SI-3). Since beam trawling is generally practised by vessels with a licence for bottom otter trawling, and the EUFR does not report *Rapido* trawling licenses, the OTB category incorporated TBB gear. Classification precision ranged from 66% (PTM) to 99% (OTB). The lower precision for PTM was due to its large confusion with OTB, whereas for PS (84% precision) the misclassification was balanced between OTB and OTHER. Our classification algorithm showed the highest precision for OTB, which was also the category with the largest number of registered and estimated gear types. Most of the OTB misclassification fell within OTHER and was due to gillnets and longlines, which share similar speed and spatial patterns. The high sensitivity for PTM, PS and OTB involved a generally small number of false negatives, except for OTHER (53% sensitivity), where the confusion was greater but uniform. The lower performance for OTHER was principally due to its under-representation in the data. Finally, the generally high specificity across categories (96-98%) indicates a very high true negative detection, i.e. agreement on the fact that a trip did not use a given gear. Based on the confusion matrix, Cohen's Kappa was 0.82, indicating *excellent* agreement[71] between AIS-estimated and EUFR-licensed gear over the five gear categories, hence a reasonable reliability of our classification algorithm.

4.3 Spatio-temporal analysis of fishing effort

4.3.1 Coarse-scale pattern analysis on Italian seas

The log-normal analysis of the GFW data of the Italian seas, described in Section 3.3, highlighted March-April effort pattern differences in 2019 and 2020 at a resolution of 0.5° (Figure 1 in the main document). Effort reduction can be generally observed in all Italian seas, but some locations still report a high fishing effort with thousands of hours. A higher resolution analysis of the AIS data for the Adriatic further explored this pattern. First, we visually compared the distributions of summed estimated fishing hours in 0.1° cells during the lockdown and non-lockdown months (Figure 3-a-b in the main document). In particular, we compared the annual aggregated February data (Figure 3-a) to those of March-May (Figure 3-b) in 2015 and 2020, and classified the high-effort locations using the 2015 log-normal ranges to produce comparable distributions over the years. The maps thus obtained show that, up to February 2020, fishing activity was equal to or higher than the previous years with a localised increase in the south-western Adriatic. In contrast, the high-effort cells were much fewer in the 2020 map, reflecting the lockdown effect in the March-May comparisons. The high-effort cells are consistent with those detected by the analysis of GFW data, but the higher resolution of the analysis indicates that these cells are actually more scattered.

4.3.2 Annual and monthly pattern analysis

In a further analysis, we compared the summed distributions of annual and monthly fishing hours in the whole Adriatic and in the high-effort cells (Figure SI-1-a-c). The comparison highlighted an increasingly intense fishing activity up to February 2020 (Figure SI-1-a-All Fleets), the effort being comparable to the level recorded in 2019 in the high-effort locations (Figure SI-1-b-All Fleets). The lockdowns reduced fishing activity proportionally both in the high-effort locations and in the whole Adriatic (Figure SI-1-a-b). The total number of fishing hours was lower in 2020 than in 2019 (-4.7%, from 985,356 to 939,229 hours, Figure SI-1-c-All Fleets).

The monthly analysis identified a sharp reduction of fishing hours after the start of the March 2020 lockdowns (Figure SI-2-All Fleets) and a general recovery in May 2020, although not to the levels of 2019. The monthly patterns changed over the years due to regulations and decommissioning (especially in 2018, see Section 4.1). The scenario of February 2020 was very similar to the one of February 2019. Clearly, the lockdowns strongly reduced fishing hours from February to March 2020 (-37%, from 87,672 to 54,798 hours, Figure SI-2-All Fleets), but their recovery in May 2020 was fast (-22% compared with February, from 87,672 to 68,246 hours). The patterns in the high-effort locations reflect those of the whole Adriatic, with a stronger reduction between February and March 2020 (-35%, from 34,794 to 22,657 hours) and a smaller difference between February and May 2020 (-7%, from 34,794 to 32,432 hours). Thus, the fisheries recovered faster in the most intensely exploited areas than in the whole Adriatic Sea. In the second half of 2020 (Section S1), the total number of fishing hours returned to the average levels of 2019, indicating that the restrictions affected fishing activities only in the lockdown period.

4.3.3 Annual and monthly pattern analysis per fleet

To establish whether the patterns observed for all the fleets were shared by each fleet, we performed a spatio-temporal analysis for each fishing gear, which demonstrated a variety of patterns. The most important variations per fleet are summarised in Table SI-4. For example, the PS effort decreased considerably from 2017 to 2018 in the whole Adriatic (-21%, from 132,335 to 104,567 hours, Figure SI-1-c-PS), but rose steeply from February 2019 to February 2020 (+60%, from 2893 to 7269 hours, Figure SI-2-PS). Furthermore, the monthly trend in the high-effort areas indicated that the PS fleet fished in fewer areas, where the effort strongly increased, especially in February 2020 (+88% hours vs. February 2019, from 327 to 2,717 hours, Figure SI-2-PS). The total number of fishing hours was slightly lower in March-May 2020 than in March-May 2019 (-10%, from 25,814 to 23,143 hours, Figure SI-1-a-PS). The monthly trends changed slightly during the 2020 lockdowns, when fishing activity was similar to the one of 2019, e.g., in March 2020 it was -11% compared with March 2019 (from 11,430 to 10,224 hours, Figure SI-2-PS and Section S1). The other months showed seasonal variations and effort levels similar to those of the previous years (Figure SI-1-c-PS and Section S1). Altogether, this analysis indicates that the PS fishery was little affected by the lockdown restrictions. The TBB fleet showed a similar effort in February 2020 and 2019 (+5% in 2020, from 10,110 to 10,647 hours, Figure SI-1-a-TBB), with a greater difference in the high-effort areas (-34% in February 2020, from 6,238 to 4,099 hours, Figure SI-1-b-TBB). However, the lockdowns strongly reduced the overall effort in March-May 2020 (-44% vs. March-May 2019, from 27,528 to 15,304 hours, Figure SI-1-a-TBB). The monthly patterns indicate a sharp reduction of fishing hours after 2018 (-40%, Figure SI-2-TBB). During the 2020 lockdowns, the number of fishing hours was considerably lower than in 2019 (-30%), but in May the gap diminished (-15% vs. 700 May 2019, from 9,023 to 7,698 hours, Figure SI-2-TBB). The situation in the high-effort fishing locations reflected in proportion the one of the whole Adriatic Sea (Figure SI-1-b-TBB). The annual and monthly values indicated that the fishing effort in the entire 2020 was always lower than in 2019 (Figure SI-1-c-TBB and Section S1). Thus, the TBB fishery was strongly impacted by the lockdowns and made a slow recovery. The PTM fleet showed a decreasing effort in February 2020, both in the whole Adriatic (-8% vs. 2019, from 2836 to 2603 hours, Figure SI-1-a-PTM) and in the high-effort locations (-15%, from 1161 to 985 hours, Figure SI-1-b-PTM). In March-May 2020, the lockdowns reduced the fishing effort more in the high-effort locations (-56% hours vs. 2019, from 3881 to 1717 hours, Figure SI-1-b-PTM) than in the whole Adriatic Sea (-39% vs. 2019, from 7845 to 4775 hours, Figure SI-1-a-PTM). Before 2018, the monthly trends were consistently around 4000 hours, whereas after 2018 there was no clear pattern (Figure SI-2-PTM). During the 2020 lockdowns the fishing effort plummeted from February to March (-48%, from 2603 to 1342 hours, Figure SI-2-PTM) but it quickly recovered in May (-1% vs. May 2019, from 1758 to 1733 hours, Figure SI-2-PTM) and subsequently mirrored that of 2019 (Figure SI-1-c-PTM and Section S1). Between February and May 2020, also the monthly hours at the high-effort locations showed a 46% reduction (from 985 to 535 hours, Figure SI-2-PTM). Altogether, the PTM fleet was moderately affected by the lockdowns and made a swift recovery in the whole Adriatic. The OTB effort showed a rising trend in February 2020 (+12% vs. 2019, from 59,206 to 67,154 hours, Figure SI-1-a-OTB), a slight decrease in the high-effort locations (-4%, from 27,986 to 26,993 hours, Figure SI-1-b-OTB), and an altogether very intense activity. The 2020 lockdowns strongly reduced the overall fishing effort of OTB vessels both in the whole Adriatic Sea (-32% vs. 2019, from 203,129 to 139,113 hours, Figure SI-1-a-OTB) and in the high-effort locations (-37%, from 102,940 to 64,516 hours, Figure SI-1-b-OTB). Rising monthly trends were identified from February to May up to 2019, whereas the 2020 lockdowns sharply reduced fishing hours from February to March (-41%, from 67,154 to 39,396 hours, Figure SI-2-OTB), with a recovery in May 2020 that did not reach the level of 2019 (-17%, from 68,989 to 56,965 hours, Figure SI-2-OTB). The scenario in the high-effort fishing locations was similar to the one of the whole Adriatic, with a reduction between February and March 2020 (-39%, from 26,993 to 16,341 hours, Figure SI-2-OTB) and a linear increase until May 2020 (-21% vs. 2019, from 34,175 to 26,772 hours, Figure SI-2-OTB). The OTB fleet returned to pre-lockdown levels only at the end of 2020 (Section S1) and was therefore strongly impacted by the lockdowns.

4.3.4 Barycentre shifts during the 2020 lockdowns

We calculated the barycentres of monthly fleet activity between 2015 and 2020 (Figure 4 in the main document and Section S2) based on the AIS dataset. For each fleet, we traced a bounding box around the barycentre to indicate the area encompassing its mean location in the period analysed. The TBB, OTB, and PS barycentres had a focus area of $\sim 1°$ latitudinal range, whereas the latitudinal range of the PTM barycentre was $\sim 2°$. The barycentre shifts over time suggested the following considerations: the TBB fleet was active mainly in the north-western Adriatic and close to the Italian coasts (Figure 4-TBB), because this gear is commonly used by Italian fleets in shallow and sandy bottoms to catch common sole and some target shellfish species. In August, during the summer fishing ban, the barycentre moved southward. The 2020 lockdowns altered the TBB barycentre pattern and extended its range compared with the previous years. As regards the PS fleet, since these vessels are more abundant in Croatia, the barycentres are very close to the Croatian coast (Figure 4-PS). The barycentre usually moved southward, away from the coast, from February to May and then returned to the north at the end of the year (Section S2). However, during the 2020 lockdowns it moved northward and returned to the south only at the end of the year. The inversion compared with 2017-2019 was likely due to the pandemic restrictions. The barycentre of the PTM fleet was concentrated in the northern-mid Adriatic and shifted north from February to May (Figure 4-PTM). During the 2020 lockdowns it moved in a more northward direction, with slightly longer monthly shifts compared with the previous years, reflecting a greater geographical range. Moreover, it was more uniformly distributed over the latitudinal range compared with the previous years. Finally, the main location of the OTB barycentre was in the south-middle Adriatic (especially in August), with small shifts (Figure 4-OTB in the main document). During the 2020 lockdowns the pattern changed in March and April due to an increased activity range compared with the previous years and remained in this spatial range for the rest of 2020 (Section S2).

4.3.5 Impact on ETP Species

Several ETP species found in the OBIS database and the IUCN Red List live in the Adriatic Sea. They include the common smooth-hound (*Mustelus mustelus*), the loggerhead sea turtle (*Caretta caretta*), the European eel (*Anguilla anguilla*), and the spurdog (*Squalus acanthias*). In February and March-May the high-effort fishing areas and those inhabited by ETP species show a strong overlap (Figure 1-b and Figure 5-a-b in the main document). An evident effect of the 2020 lockdowns, detected both in the GFW and the AIS datasets, was a scattering and overall reduction of the overlap during this period. Specifically, in February 2020 in the Adriatic Sea the fishing hours in the locations at higher risk of impact were much fewer than in February 2019 (-35%, from 16,629 to 10,793 hours, Figure 5-a and Figure SI-3-a-All Fleets), but higher than in February 2018 (+24%, from 8,176 to 10,793 hours, Figure SI-3-a-All Fleets), whereas in March-May 2020 they were much fewer than in 2019 (-59%, from 38,577 to 15,987 hours, Figure SI-3-a-All Fleets). Analysis of the monthly trends demonstrated that the number of fishing hours at these locations during the lockdowns was generally lower than in the previous years, and in May 2020 did not reach the level of 2019 (-35% vs. May 2019, from 13,302 to 8,620 hours, Figure SI-3-b-All Fleets). Although after the lockdowns the total fishing effort reverted to 2019 levels (Section 4.3.2), the fishing hours at the locations at higher risk of impact remained below pre-lockdown levels throughout 2020 (Sections S3 and S4), indicating a general attenuation of impact on ETP species in the Adriatic Sea during the pandemic.

However, this was not true of all gear types. As summarised in Table SI-5, PS fishing hours increased at the locations at higher risk of impact up to February 2020 (Figure SI-3-a-PS) and remained constant, and well above 2019 values, both in March-May (around 240 hours, Figure SI-3-b-PS) and in the second half of 2020 (Section S4). In February 2020 the TBB fleet showed a reduction of fishing hours at the locations at higher risk of impact compared with February 2019 (-19%, from 6,262 to 5,046 hours, Figure SI-3-a-TBB) as well as a strong reduction from February to April 2020 (-77%, from 6,262 to 5,046 hours, Figure SI-3-b-TBB); the fishing effort did not revert to 2019 levels either in May 2020 (-21% vs. May 2019, from 5,223 to 4,132 hours, Figure SI-3-b-TBB), or in the second semester (Section S4). In February 2020 the PTM fleet had already reduced its effort at the locations at higher risk of impact (-51% vs. February 2019, from 314 to 154 hours; and -20% vs. February 2018, from 192 to 154 hours, Figure SI-3-a-PTM). However, a strong reduction from February to March 2020 (-47%, from 154 to 81, Figure SI-3-b-PTM) was followed by a non-linear increase up to May 2020 that almost reached 2019 values (-13% vs. May 2019, from 771 to 673 hours, Figure SI-3-b-PTM), and remained high also for the rest of the year (Section S4). Finally, the fishing effort of the OTB fleet was lower in February 2020 than in February 2019 (-46%, from 9,923 to 5,354 hours, Figure SI-3-a-OTB), although it was slightly higher than in 2018 (+12%, from 4,722 to 5,354 hours, Figure SI-3-b-OTB). A strong reduction from February to March 2020 (-69%, from 5,354 to 1,669 hours, Figure SI-3-b-OTB) was followed by a slow increase up to May 2020 (-50% vs. May 2019, from 7,183 to 3,574 hours), but the fishing effort of this fleet remained well below 2019 levels also in the rest of the year (Section S4).

5 Project Information

The present study is part of the Snapshot-CNR project (of which M. Sprovieri and F. Trincardi are initiators and project leaders), whose aim is to provide a quantitative assessment of the effects of the reduced anthropogenic pressure on marine systems during

the lockdowns that responded to the COVID-19 pandemic[72]. The 2020 restrictions generated unprecedented, and partially unexpected, human and marine ecosystem dynamics at various levels besides those related to fisheries. By analysing these dynamics in the Italian marine ecosystems, specific cause-effect relationships can be identified and extended to other world ecosystems. The aim of the project is to measure these relationships and the multiple factors involved – including pollution, the economy, fisheries and ecosystem services – to design novel strategies for a more sustainable future.

References

1. Witt, M. J. & Godley, B. J. A step towards seascape scale conservation: using vessel monitoring systems (vms) to map fishing activity. *PloS one* **2**, e1111 (2007).
2. Gerritsen, H. & Lordan, C. Integrating vessel monitoring systems (VMS) data with daily catch data from logbooks to explore the spatial distribution of catch and effort at high resolution. *ICES J. Mar. Sci.* **68**, 245–252, DOI: 10.1093/icesjms/fsq137 (2010). https://academic.oup.com/icesjms/article-pdf/68/1/245/29138833/fsq137.pdf.
3. Lee, J., South, A. B. & Jennings, S. Developing reliable, repeatable, and accessible methods to provide high-resolution estimates of fishing-effort distributions from vessel monitoring system (VMS) data. *ICES J. Mar. Sci.* **67**, 1260–1271, DOI: 10.1093/icesjms/fsq010 (2010). https://academic.oup.com/icesjms/article-pdf/67/6/1260/29136804/fsq010.pdf.
4. Lambert, G. I. *et al.* Implications of using alternative methods of vessel monitoring system (VMS) data analysis to describe fishing activities and impacts. *ICES J. Mar. Sci.* **69**, 682–693, DOI: 10.1093/icesjms/fss018 (2012). https://academic.oup.com/icesjms/article-pdf/69/4/682/29143402/fss018.pdf.
5. Russo, T., D'Andrea, L., Parisi, A. & Cataudella, S. Vmsbase: an r-package for vms and logbook data management and analysis in fisheries ecology. *PLoS One* **9**, e100195 (2014).
6. Coro, G., Large, S., Magliozzi, C. & Pagano, P. Analysing and forecasting fisheries time series: purse seine in indian ocean as a case study. *ICES J. Mar. Sci.* **73**, 2552–2571 (2016a).
7. EC. COUNCIL REGULATION (EC) No 1224/2009 of 20 November 2009 (2009). http://eur-lex.europa.eu/LexUriServ/LexUriServ.do?uri=OJ:L:2009:343:0001:0050:EN:PDF.
8. Bastardie, F., Nielsen, J. R., Ulrich, C., Egekvist, J. & Degel, H. Detailed mapping of fishing effort and landings by coupling fishing logbooks with satellite-recorded vessel geo-location. *Fish. Res.* **106**, 41–53, DOI: https://doi.org/10.1016/j.fishres.2010.06.016 (2010).
9. O'Farrell, S. *et al.* Improving detection of short-duration fishing behaviour in vessel tracks by feature engineering of training data. *ICES J. Mar. Sci.* **74**, 1428–1436, DOI: 10.1093/icesjms/fsw244 (2017). https://academic.oup.com/icesjms/article-pdf/74/5/1428/31243625/fsw244.pdf.
10. Postlethwaite, C. M. & Dennis, T. E. Effects of temporal resolution on an inferential model of animal movement. *PLoS One* **8**, e57640 (2013).
11. Vermard, Y., Rivot, E., Mahévas, S., Marchal, P. & Gascuel, D. Identifying fishing trip behaviour and estimating fishing effort from vms data using bayesian hidden markov models. *Ecol. Model.* **221**, 1757–1769 (2010).
12. McCauley, D. J. *et al.* Ending hide and seek at sea. *Science* **351**, 1148–1150, DOI: 10.1126/science.aad5686 (2016). https://science.sciencemag.org/content/351/6278/1148.full.pdf.
13. Russo, T. *et al.* Assessing the fishing footprint using data integrated from different tracking devices: Issues and opportunities. *Ecol. Indic.* **69**, 818–827, DOI: https://doi.org/10.1016/j.ecolind.2016.04.043 (2016).
14. Ferrà, C. *et al.* Mapping change in bottom trawling activity in the mediterranean sea through ais data. *Mar. Policy* **94**, 275–281 (2018).
15. Puig, P. *et al.* Ploughing the deep sea floor. *Nature* **489**, 286–289 (2012).
16. Bergh, P. E. & Davies, S. Fishery monitoring, control and surveillance. *FAO Fish. Tech. Pap.* 175–204 (2002).
17. Gianelli, I., Horta, S., Martinez, G., de la Rosa, A. & Defeo, O. Operationalizing an ecosystem approach to small-scale fisheries in developing countries: The case of uruguay. *Mar. Policy* **95**, 180–188 (2018).
18. Lockerbie, E. M., Lynam, C. P., Shannon, L. J. & Jarre, A. Applying a decision tree framework in support of an ecosystem approach to fisheries: Indiseas indicators in the north sea. *ICES J. Mar. Sci.* **75**, 1009–1020 (2018).
19. Muawanah, U. *et al.* Review of national laws and regulation in indonesia in relation to an ecosystem approach to fisheries management. *Mar. Policy* **91**, 150–160 (2018).

20. Koen-Alonso, M., Pepin, P., Fogarty, M. J., Kenny, A. & Kenchington, E. The northwest atlantic fisheries organization roadmap for the development and implementation of an ecosystem approach to fisheries: structure, state of development, and challenges. *Mar. Policy* **100**, 342–352 (2019).

21. Coro, G., Fortunati, L. & Pagano, P. Deriving fishing monthly effort and caught species from vessel trajectories. In *2013 MTS/IEEE OCEANS-Bergen*, 1–5 (IEEE, 2013).

22. de Souza, E. N., Boerder, K., Matwin, S. & Worm, B. Improving fishing pattern detection from satellite ais using data mining and machine learning. *PloS one* **11**, e0158248 (2016).

23. Pallotta, G., Vespe, M. & Bryan, K. Vessel pattern knowledge discovery from ais data: A framework for anomaly detection and route prediction. *Entropy* **15**, 2218–2245 (2013).

24. Coro, G. Open science and artificial intelligence supporting blue growth. *Environ. Eng. & Manag. J. (EEMJ)* **19** (2020).

25. Campanis, G. Advancements in vms data analyses. *NAFO Annu. Rep. for 2008* (2008). Accessible online at https://www.nafo.int/Portals/0/PDFs/ar/ar08.pdf?ver=2016-02-10-101458-760.

26. Natale, F., Gibin, M., Alessandrini, A., Vespe, M. & Paulrud, A. Mapping fishing effort through ais data. *PloS one* **10**, e0130746 (2015).

27. Vespe, M. *et al.* Mapping eu fishing activities using ship tracking data. *J. Maps* **12**, 520–525, DOI: 10.1080/17445647.2016.1195299 (2016). https://doi.org/10.1080/17445647.2016.1195299.

28. Joo, R., Bertrand, S., Tam, J. & Fablet, R. Hidden markov models: the best models for forager movements? *PLoS One* **8**, e71246 (2013).

29. Kroodsma, D. *et al.* Tracking the global footprint of fisheries. *Science* **359**, 904–908, DOI: 10.1126/science.aao5646 (2018). https://science.sciencemag.org/content/359/6378/904.full.pdf.

30. Kroodsma, D., Miller, N. A., Hochberg, T., Park, J. & Clavelle, T. Global atlas of ais-based fishing activity - challenges and opportunities (2019). http://www.fao.org/3/ca7012en/CA7012EN.pdf.

31. Kim, K.-i. & Lee, K. M. Convolutional neural network-based gear type identification from automatic identification system trajectory data. *Appl. Sci.* **10**, DOI: 10.3390/app10114010 (2020).

32. Trites, A. W., Christensen, V., Pauly, D. & Camphuysen, C. J. *Effects of fisheries on ecosystems: just another top predator?*, 11–27. Conservation Biology (Cambridge University Press, 2006).

33. Fulton, E. A., Smith, A. D., Smith, D. C. & Johnson, P. An integrated approach is needed for ecosystem based fisheries management: insights from ecosystem-level management strategy evaluation. *PloS one* **9**, e84242 (2014).

34. Boonstra, W. J. & Hentati-Sundberg, J. Classifying fishers' behaviour. an invitation to fishing styles. *Fish Fish.* **17**, 78–100, DOI: https://doi.org/10.1111/faf.12092 (2016). https://onlinelibrary.wiley.com/doi/pdf/10.1111/faf.12092.

35. O'Farrell, S., Chollett, I., Sanchirico, J. N. & Perruso, L. Classifying fishing behavioral diversity using high-frequency movement data. *Proc. Natl. Acad. Sci.* **116**, 16811–16816 (2019).

36. Gari, S. R., Newton, A. & Icely, J. D. A review of the application and evolution of the dpsir framework with an emphasis on coastal social-ecological systems. *Ocean. & Coast. Manag.* **103**, 63–77 (2015).

37. Song, A. M., Johnsen, J. P. & Morrison, T. H. Reconstructing governability: How fisheries are made governable. *Fish Fish.* **19**, 377–389 (2018).

38. Pranovi, F., Raicevich, S., Franceschini, G., Farrace, M. G. & Giovanardi, O. Rapido trawling in the northern Adriatic Sea: effects on benthic communities in an experimental area. *ICES J. Mar. Sci.* **57**, 517–524, DOI: 10.1006/jmsc.2000.0708 (2000). https://academic.oup.com/icesjms/article-pdf/57/3/517/1780115/57-3-517.pdf.

39. FAO-GFCM. Fishery and Aquaculture Statistics. GFCM capture production 1970-2019 (FishstatJ) (2021). FAO Fisheries Division online software https://www.fao.org/fishery/statistics/software/fishstatj/en.

40. Colloca, F. *et al.* The seascape of demersal fish nursery areas in the north mediterranean sea, a first step towards the implementation of spatial planning for trawl fisheries. *PloS one* **10**, e0119590 (2015).

41. Leonori, I., De Felice, A., Campanella, F., Biagiotti, I. & Canduci, G. Assessment of small pelagic fish biomass in the western adriatic sea by means of acoustic methodology. *Mar. research at CNR Vol. DTA/06 Fish. Sea Resour. Rome* 2019–2029 (2011).

42. Fonda, S., Franco, P., Ghirardelli, E. & Malej, A. Outline of oceanography and the plankton of the adriatic sea (1992). https://arts.units.it/handle/11368/1747095.

43. Bastardie, F. *et al.* Spatial planning for fisheries in the northern adriatic: working toward viable and sustainable fishing. *Ecosphere* **8**, e01696 (2017).

44. Fortibuoni, T. *et al.* Fish and fishery historical data since the 19th century in the adriatic sea, mediterranean. *Sci. data* **4**, 1–13 (2017).

45. Armelloni, E. N. *et al.* Ais data, a mine of information on trawling fleet mobility in the mediterranean sea. *Mar. Policy* **129**, 104571 (2021).

46. FAO. GFCM Recommendation 37/2013/1 (2013). http://www.fao.org/3/ax394e/ax394e.pdf.

47. FAO. GFCM Recommendation GFCM/43/2019/5 (2019). http://www.fao.org/gfcm/decisions/en/.

48. Italian Council of Ministers. G.U. n.64 11-3-2020 (2020). https://www.gazzettaufficiale.it/eli/gu/2020/03/11/64/sg/pdf.

49. Italian Council of Ministers. GU n.76 22-3-2020 (2020). https://www.gazzettaufficiale.it/eli/gu/2020/03/22/76/sg/pdf.

50. European Council. COVID-19 coronavirus pandemic: the EU's response (2020). https://www.policija.si/eng/newsroom/news-archive/103470-crossing-the-state-border-during-the-coronavirus-epidemic.

51. Slovenian Government. Crossing the state border during the coronavirus epidemic (2020). https://www.policija.si/eng/newsroom/news-archive/103470-crossing-the-state-border-during-the-coronavirus-epidemic.

52. Merten, W. *et al.* Global fishing watch: Bringing transparency to global commercial fisheries. *arXiv preprint arXiv:1609.08756* 1–4 (2016).

53. Shepperson, J. L. *et al.* A comparison of vms and ais data: The effect of data coverage and vessel position recording frequency on estimates of fishing footprints. *ICES J. Mar. Sci.* **75**, 988–998 (2018).

54. Clavelle, T. Global fisheries during COVID-19 (2020). https://globalfishingwatch.org/data-blog/global-fisheries-during-covid-19/.

55. Coll, M., Ortega-Cerdà, M. & Mascarell-Rocher, Y. Ecological and economic effects of covid-19 in marine fisheries from the northwestern mediterranean sea. *Biol. Conserv.* **255**, 108997 (2021).

56. Love, D. C. *et al.* Emerging covid-19 impacts, responses, and lessons for building resilience in the seafood system. *Glob. Food Secur.* 100494 (2021).

57. Astra Paging Ltd. Astra Paging Data Service (2020). http://www.astrapaging.com/data-services.

58. GFCM. Geographical subareas of the General Fisheries Commission for the Mediterranean (2020). http://www.fao.org/gfcm/data/maps/gsas/en/.

59. Data Europa. EU Fleet Register (2020). https://data.europa.eu/euodp/it/data/dataset/the-community-fishing-fleet-register.

60. Saha, B. & Srivastava, D. Data quality: The other face of big data. In *2014 IEEE 30th international conference on data engineering*, 1294–1297 (IEEE, 2014).

61. Galdelli, A. *et al.* A cloud computing architecture to map trawling activities using positioning data. In *International Design Engineering Technical Conferences and Computers and Information in Engineering Conference*, vol. 59292, V009T12A035 (American Society of Mechanical Engineers, 2019).

62. Ho, T. K. Random decision forests. In *Proceedings of 3rd international conference on document analysis and recognition*, vol. 1, 278–282 (IEEE, 1995).

63. Cohen, J. *et al.* A coefficient of agreement for nominal scales. *Educ. psychological measurement* **20**, 37–46 (1960).

64. Grassle, J. F. The ocean biogeographic information system (obis): an on-line, worldwide atlas for accessing, modeling and mapping marine biological data in a multidimensional geographic context. *Oceanography* **13**, 5–7 (2000).

65. Coro, G. *et al.* Estimating absence locations of marine species from data of scientific surveys in obis. *Ecol. Model.* **323**, 61 – 76, DOI: https://doi.org/10.1016/j.ecolmodel.2015.12.008 (2016b).

66. Ester, M., Kriegel, H.-P., Sander, J., Xu, X. *et al.* A density-based algorithm for discovering clusters in large spatial databases with noise. In *Kdd*, vol. 96, 226–231 (1996).

67. Froese, R., Demirel, N., Coro, G., Kleisner, K. M. & Winker, H. Estimating fisheries reference points from catch and resilience. *Fish Fish.* **18**, 506–526 (2017).

68. Schaefer, M. Fisheries dynamics and the concept of maximum equilibrium catch. In *Proceedings of the Gulf and Caribbean Fisheries Institute*, vol. 6, 53–64 (Gulf and Caribbean Fisheries Institute, 1954).

69. Froese, R. *et al.* Estimating stock status from relative abundance and resilience. *ICES J. Mar. Sci.* **77**, 527–538 (2020).

70. European Commission. The 2019 Annual Economic Report on the EU Fishing Fleet (STECF 19-06) (2019). https://stecf.jrc.ec.europa.eu/reports/economic/-/asset_publisher/d7Ie/document/id/2571760.

71. Fleiss, J. L. Measuring nominal scale agreement among many raters. *Psychol. bulletin* **76**, 378 (1971).

72. Snapshot-CNR. The Snapshot-CNR project (2020). http://snapshot.cnr.it.

Table SI-1. Summary of the metrics used to assess the representativeness of EU Fleet Register (EUFR) vessels by the AIS dataset in the Adriatic Sea: Total number of vessels registered in the EUFR; Number of EUFR vessels that broadcast at least one AIS message (Expected vessels); Number of EUFR vessels included in the AIS dataset (AIS-observed vessels); Expected vessels divided by the total number of EUFR vessels (Expected coverage); AIS-observed vessels divided by the total number of EUFR vessels (Observed coverage); and AIS-observed vessels divided by the number of Expected vessels (Representativeness score).

Year	Total EUFR vessels	Expected vessels	AIS-observed vessels	Expected coverage (%)	Observed coverage (%)	Representativeness score (%)
2015	1,047	825	744	79	71	90
2016	1,031	816	761	79	74	93
2017	1,018	803	737	79	72	92
2018	932	725	648	78	70	89
2019	931	725	643	78	69	89
2020	926	724	642	78	69	89

Table SI-2. Summary of EU and non-EU vessels recorded in the Adriatic Sea AIS dataset: Total number of vessels; Total number of trips; Number of vessels estimated to use only a pelagic pair trawl (PTM), a purse seine (PS), a bottom otter trawl (OTB), a beam trawl (TBB), other non-classified gear types (OTHER) or two or more gears (MIXED).

Year	Total Vessels	Total Trips	PTM	PS	OTB	TBB	OTHER	MIXED
**2015**	866	89,778	28	117	374	44	6	297
**2016**	878	91,075	25	107	398	45	20	283
**2017**	873	84,568	33	86	399	49	4	302
**2018**	833	71,242	23	90	393	47	3	277
**2019**	827	73,805	21	86	379	48	7	286
**2020**	848	73,750	23	95	393	39	7	291

Table SI-3. Confusion matrix of our gear classification workflow executed against EUFR data for the Adriatic Sea. Each row indicates the number of vessels estimated for one gear over the EUFR-registered gears. Precision, sensitivity and specificity are reported for each gear. Acronyms refer to distinct fleets using a pelagic pair trawl (PTM), a purse seine (PS), a bottom otter trawl and a beam trawl (OTB) and other non-classified gears (OTHER).

Prediction	EUFR data				
	PTM	PS	OTB	OTHER	Total
PTM	3,066	57	1,767	16	4,906
OTHER	0	133	205	867	1,205
PS	0	6,604	669	558	7,831
OTB	14	70	29,967	199	30,250
Total	3,080	6,864	32,608	1,640	
Precision	62%	84%	99%	72%	
Sensitivity	99%	96%	92%	53%	
Specificity	96%	97%	98%	99%	

Table SI-4. Summary of relative effort change per fleet in the whole Adriatic Sea and in the high-effort fishing locations, with an indication of the overall impact strength of the 2020 lockdowns on the fisheries.

	PS	PTM	TBB	OTB
**In the whole Adriatic Sea**				
March-May 2020 vs. March-May 2019	-10%	-39%	-44%	-32%
February 2020 vs. February 2019	+60%	-8%	+5%	+12%
2020 vs. 2019	-1%	-6%	-19%	-3%
From February 2020 to March 2020	+29%	-48%	-64%	-41%
May 2020 vs. May 2019	-23%	-1%	-15%	-17%
**In the high-effort fishing locations**				
March-May 2020 vs. March-May 2019	+79%	-56%	-55%	-37%
February 2020 vs. February 2019	+88%	-15%	-34%	-4%
From February 2020 to March 2020	+34%	-46%	-47%	-39%
May 2020 vs. May 2019	+27%	-36%	-31%	-22%
Overall negative impact strength of 2020 lockdowns	**Low**	**Moderate**	**High**	**High**

Table SI-5. Summary of relative impact change on ETP species per fleet in the whole Adriatic Sea, with an indication of the overall impact of the fisheries on ETP species during the 2020 lockdowns.

	**PS**	**PTM**	**TBB**	**OTB**
March-May 2020 vs. March-May 2019	+56%	-49%	-55%	-65%
February 2020 vs. February 2019	+45%	-51%	-19%	-46%
From February 2020 to April 2020	+16%	+40%	-77%	-66%
May 2020 vs. May 2019	+48%	-13%	-21%	-50%
Overall impact on ETP species during the 2020 lockdowns	**High**	**Moderate**	**Low**	**Low**

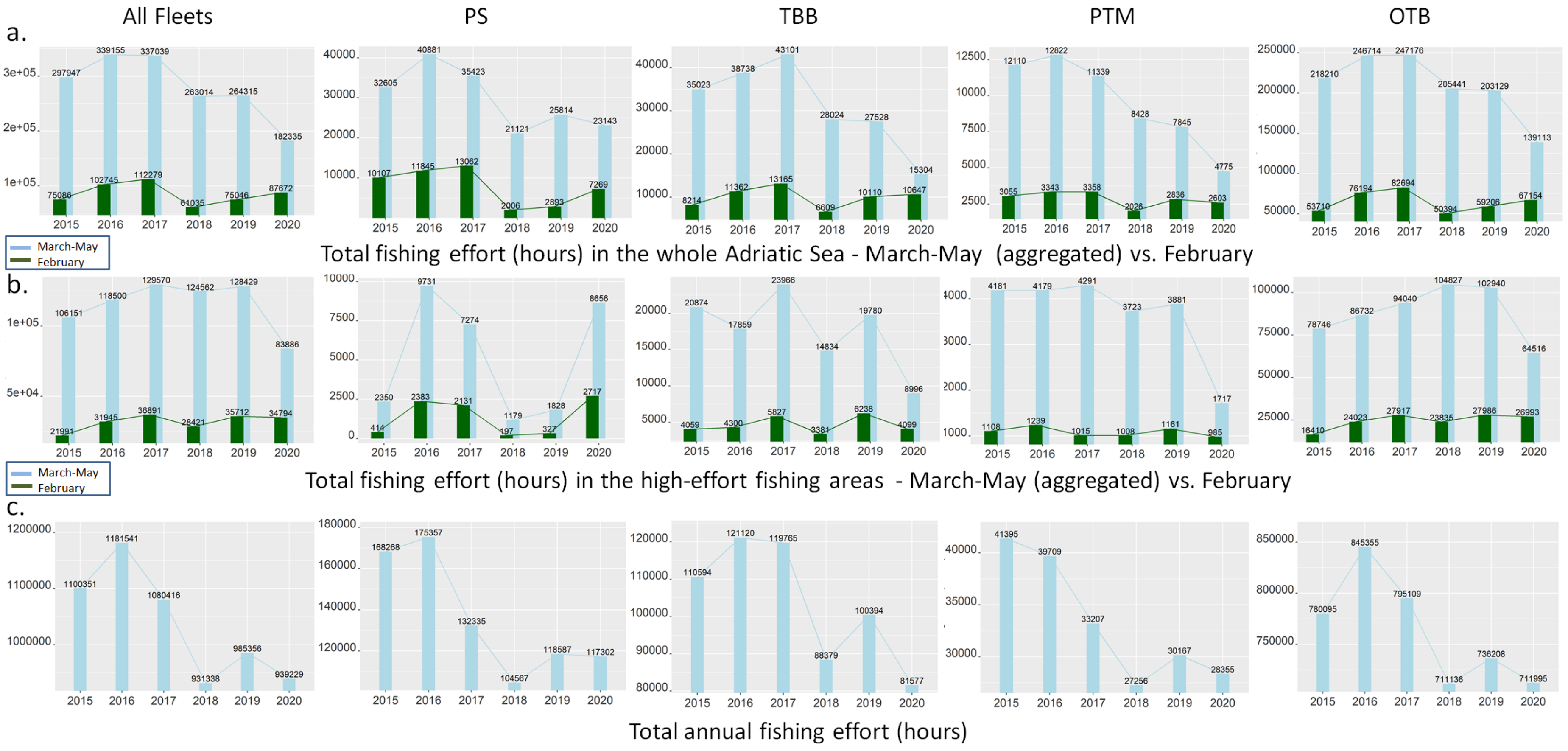

Figure SI-1. Variation of total annual fishing hours in the whole Adriatic Sea (a) in February (green) and March-May (light blue), (b) in the highest-effort fishing locations, and (c) over all months per year. Acronyms indicate distinct fleets using purse seines (PS), pelagic pair trawls (PTM), beam trawls (TBB) or bottom otter trawls (OTB) and their aggregation (All Fleets).

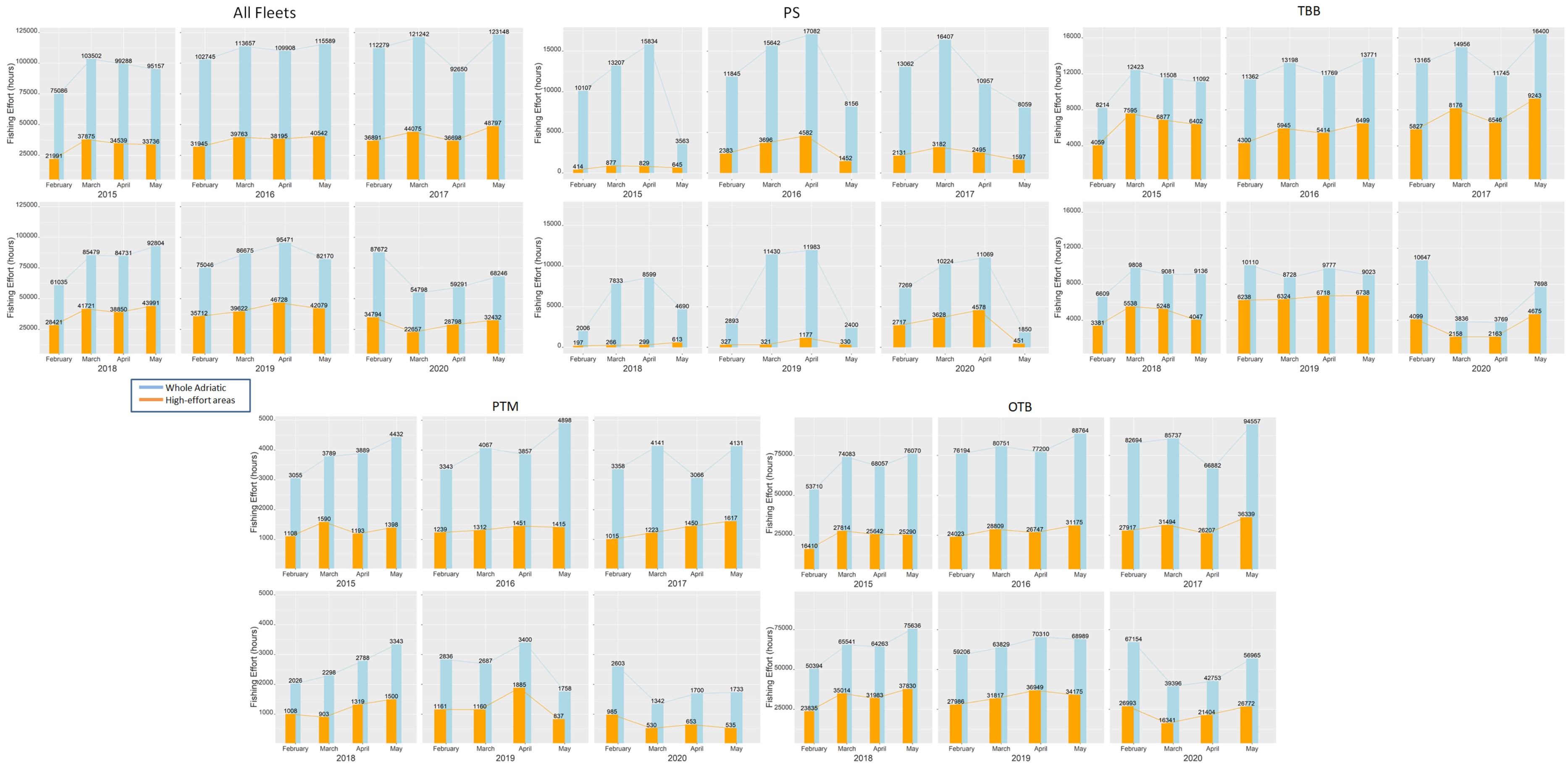

Total monthly fishing effort (hours) in the whole Adriatic Sea vs. high-effort areas – from February to May

Figure SI-2. Variation of monthly fishing hours in the whole Adriatic Sea (light blue) and in the highest-effort fishing locations (orange) per estimated gear type. Acronyms indicate fleets using purse seines (PS), pelagic pair trawls (PTM), beam trawls (TBB) or bottom otter trawls (OTB) and their aggregation (All Fleets).

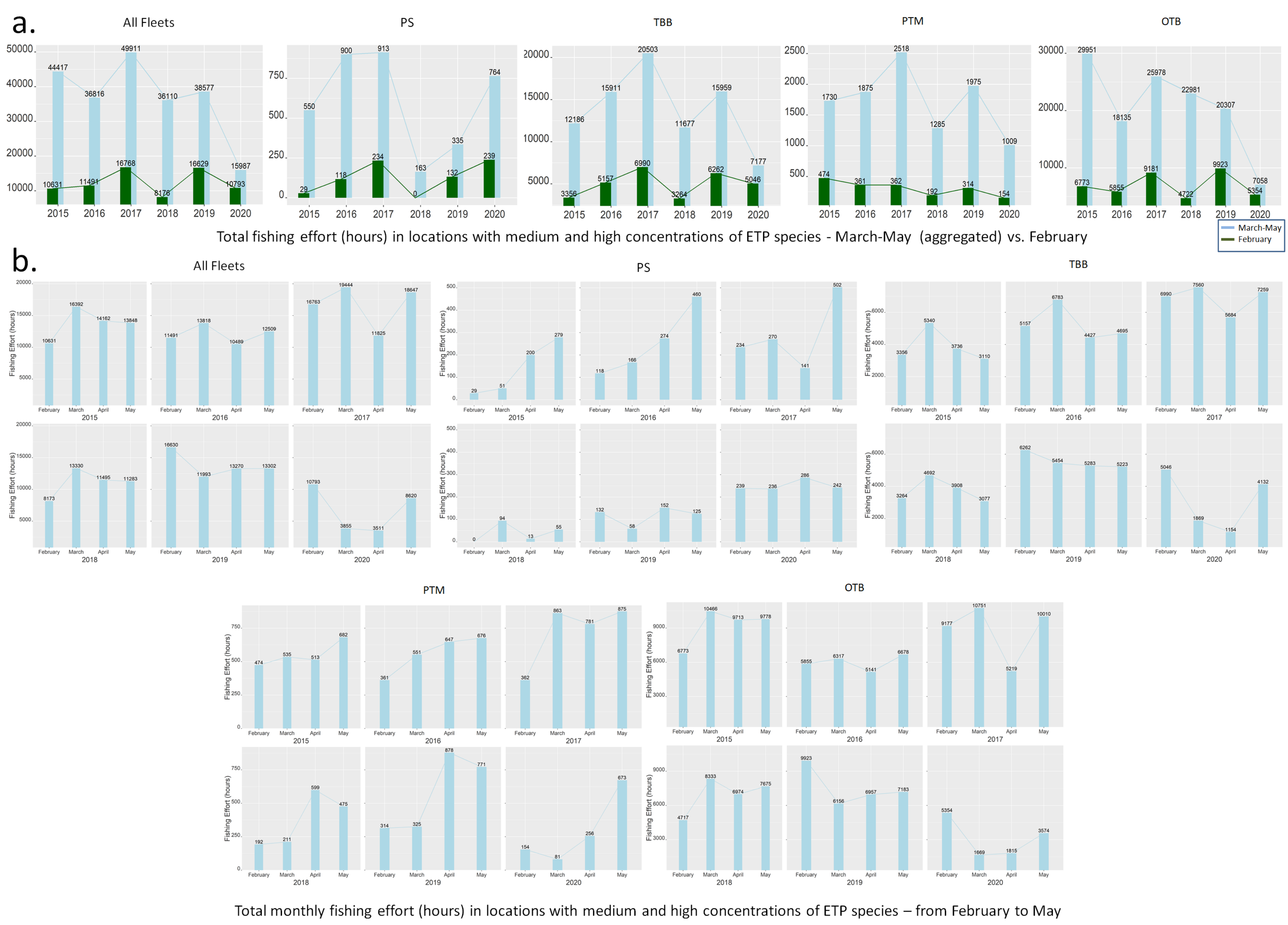

Figure SI-3. Variation of (a) annual and (b) monthly fishing hours at the Adriatic Sea locations with a medium and high number of endangered, threatened and protected (ETP) species in February-March. Acronyms refer to distinct fleets using purse seines (PS), pelagic pair trawls (PTM), beam trawls (TBB) or bottom otter trawls (OTB) and their aggregation (All Fleets).

S1 - Total monthly fishing effort (hours) in the whole Adriatic over all months

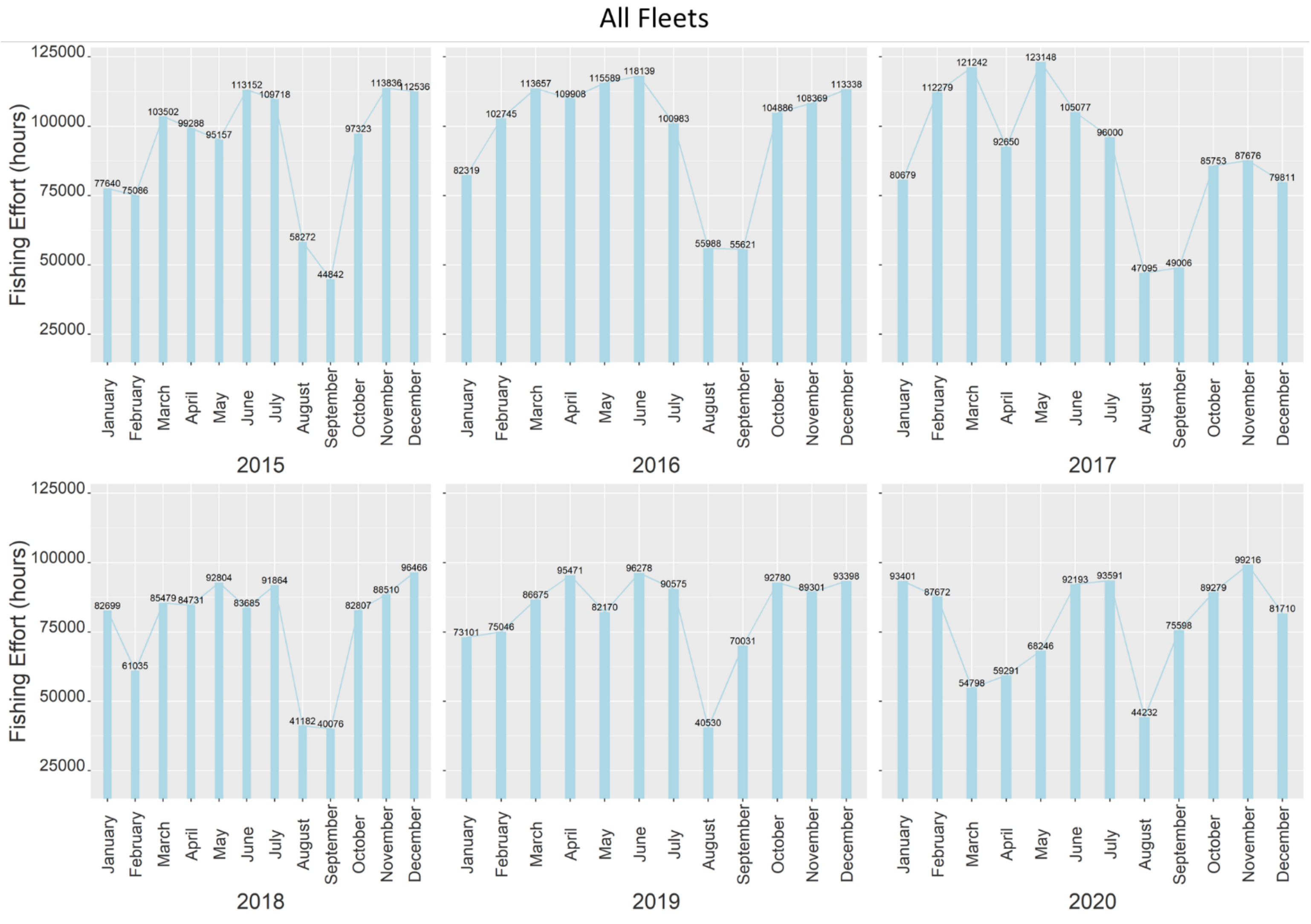

PS

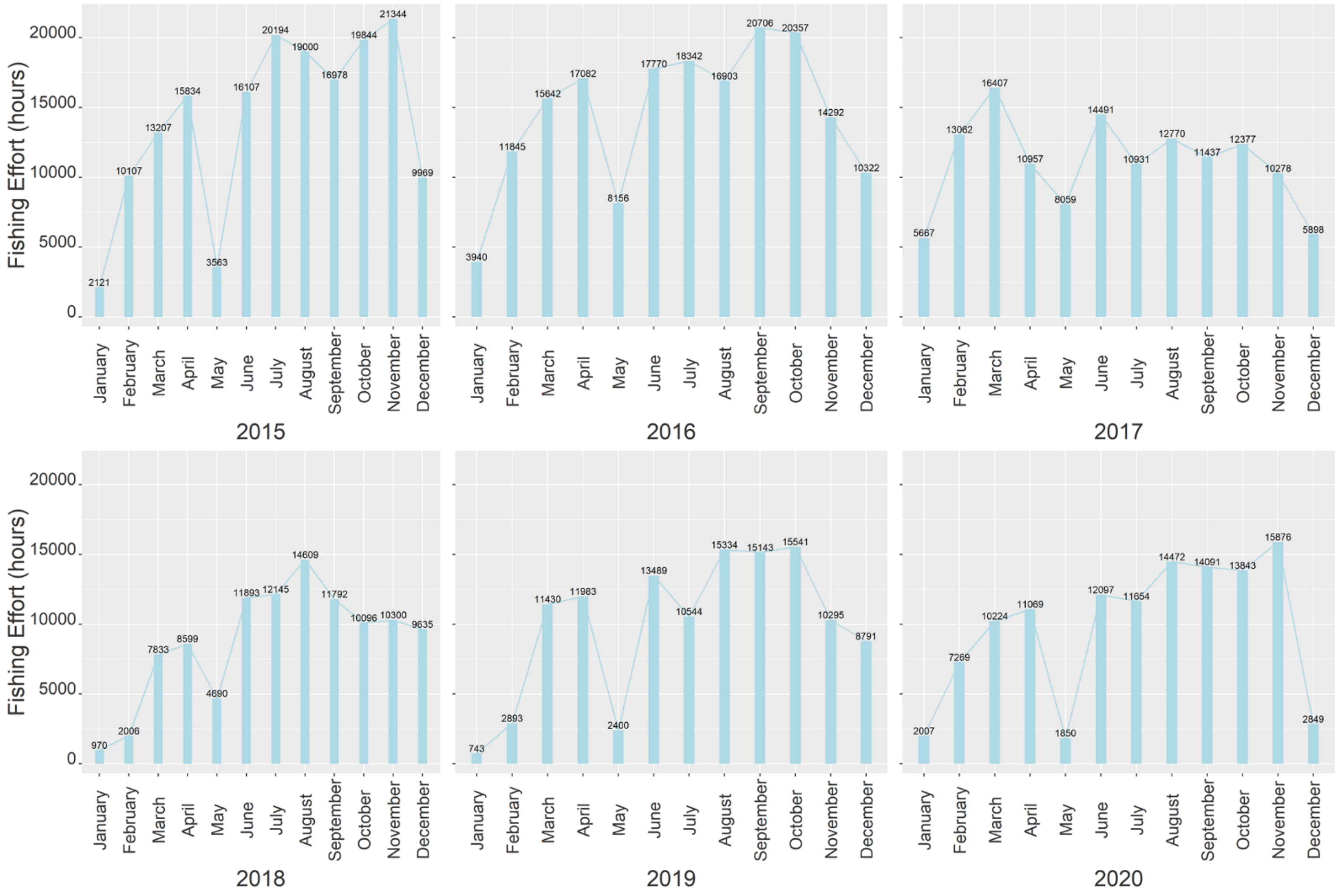
Fishing Effort (hours)
20000
15000
10000
5000
0
2121
10107
13207
15834
3563
16107
20194
19000
16978
19844
21344
9969
January
February
March
April
May
June
July
August
September
October
November
December
2015
3940
11845
15642
17082
8156
17770
18342
16903
20706
20357
14292
10322
2016
5667
13062
16407
10957
8059
14491
10931
12770
11437
12377
10278
5898
2017
970
2006
7833
8599
4690
11893
12145
14609
11792
10096
10300
9635
2018
743
2893
11430
11983
2400
13489
10544
15334
15143
15541
10295
8791
2019
2007
7269
10224
11069
1850
12097
11654
14472
14091
13843
15876
2849
2020

TBB

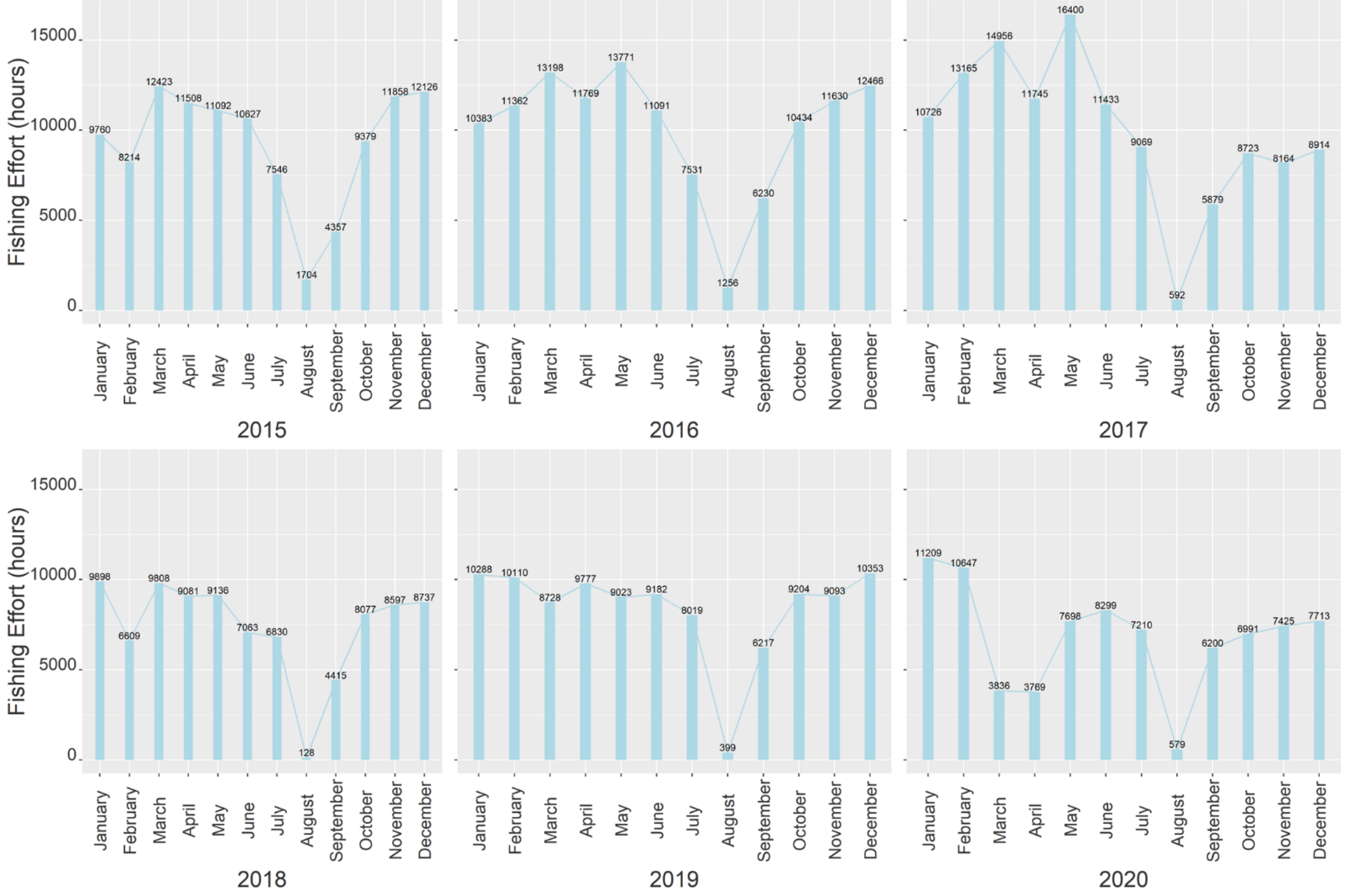

Fishing Effort (hours)
15000
10000
5000
0
January
February
March
April
May
June
July
August
September
October
November
December
2015
2016
2017
2018
2019
2020
9760
8214
12423
11508
11092
10627
7546
1704
4357
9379
11858
12126
10383
11362
13198
11769
13771
11091
7531
1256
6230
10434
11630
12466
10726
13165
14956
11745
16400
11433
9069
592
5879
8723
8164
8914
9898
6609
9808
9081
9136
7063
6830
128
4415
8077
8597
8737
10288
10110
8728
9777
9023
9182
8019
399
6217
9204
9093
10353
11209
10647
3836
3769
7698
8299
7210
579
6200
6991
7425
7713

PTM

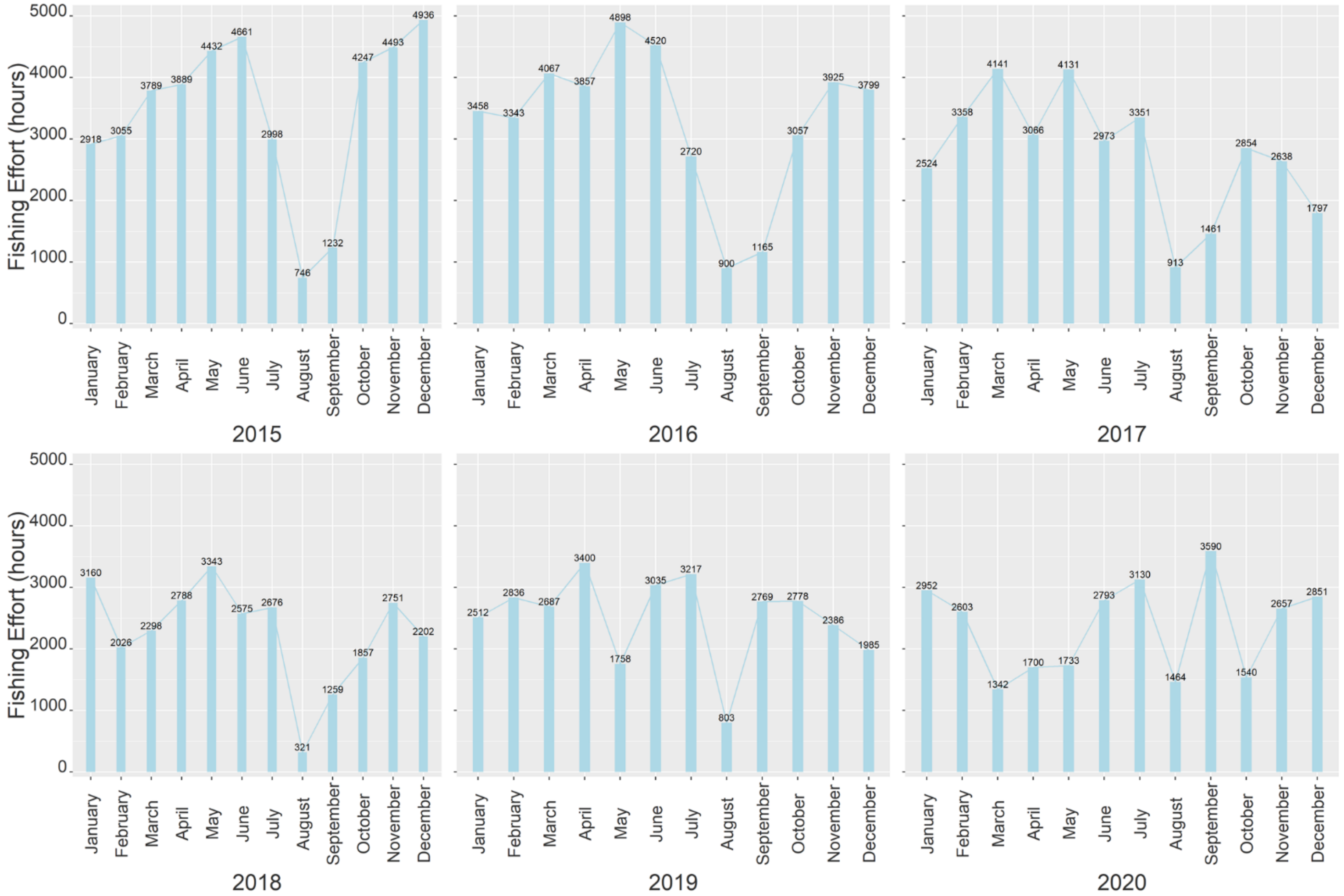
Fishing Effort (hours)
0
1000
2000
3000
4000
5000
January
February
March
April
May
June
July
August
September
October
November
December
2015
2918 3055 3789 3889 4432 4661 2998 746 1232 4247 4493 4936
2016
3458 3343 4067 3857 4898 4520 2720 900 1165 3057 3925 3799
2017
2524 3358 4141 3066 4131 2973 3351 913 1461 2854 2638 1797
2018
3160 2026 2298 2788 3343 2575 2676 321 1259 1857 2751 2202
2019
2512 2836 2687 3400 1758 3035 3217 803 2769 2778 2386 1985
2020
2952 2603 1342 1700 1733 2793 3130 1464 3590 1540 2657 2851

OTB

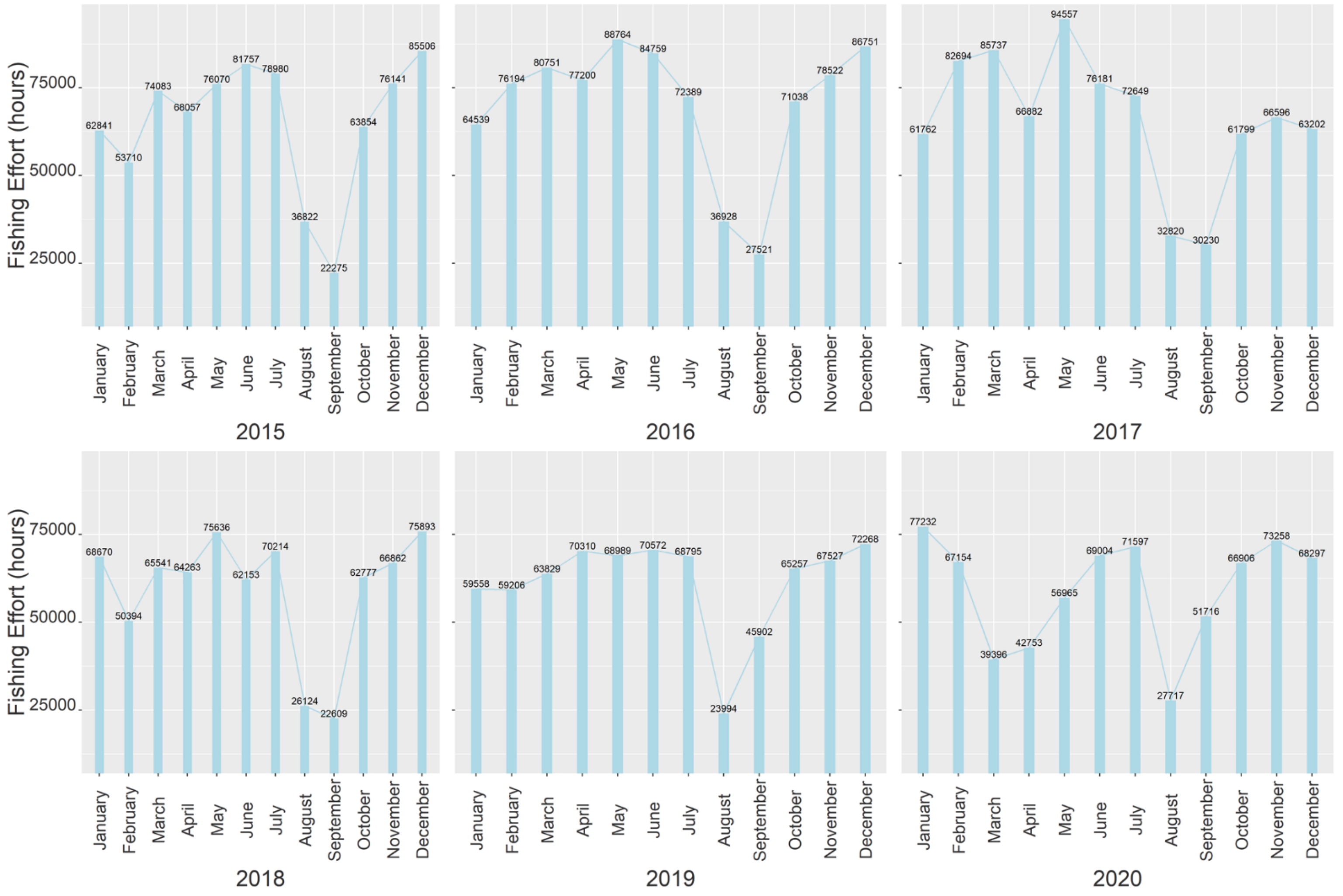
Fishing Effort (hours)
75000
50000
25000
January
February
March
April
May
June
July
August
September
October
November
December
2015
62841
53710
74083
68057
76070
81757
78980
36822
22275
63854
76141
85506
2016
64539
76194
80751
77200
88764
84759
72389
36928
27521
71038
78522
86751
2017
61762
82694
85737
66882
94557
76181
72649
32820
30230
61799
66596
63202
2018
68670
50394
65541
64263
75636
62153
70214
26124
22609
62777
66862
75893
2019
59558
59206
63829
70310
68989
70572
68795
23994
45902
65257
67527
72268
2020
77232
67154
39396
42753
56965
69004
71597
27717
51716
66906
73258
68297

S2 - Monthly barycentre shifts across the years

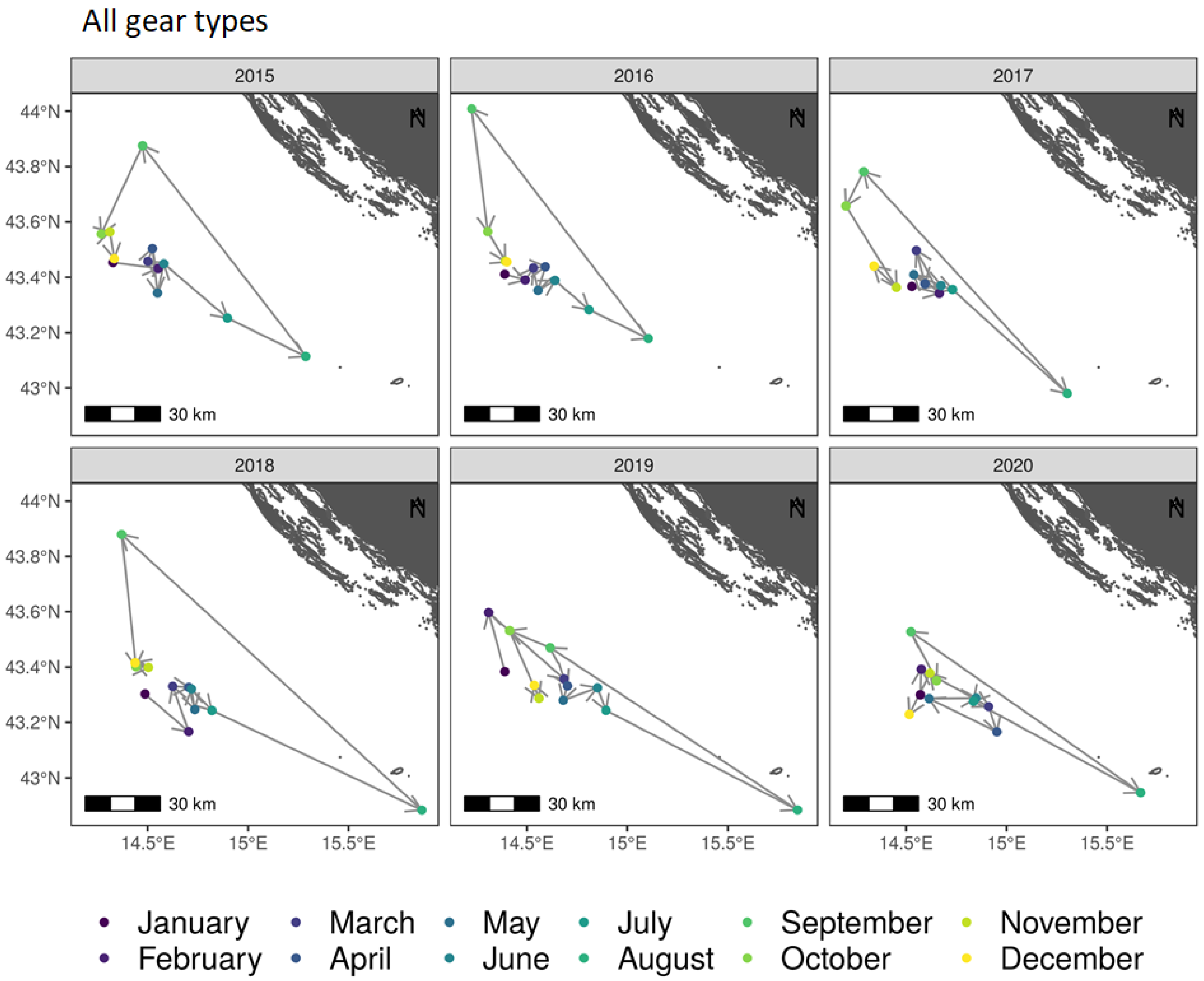

TBB

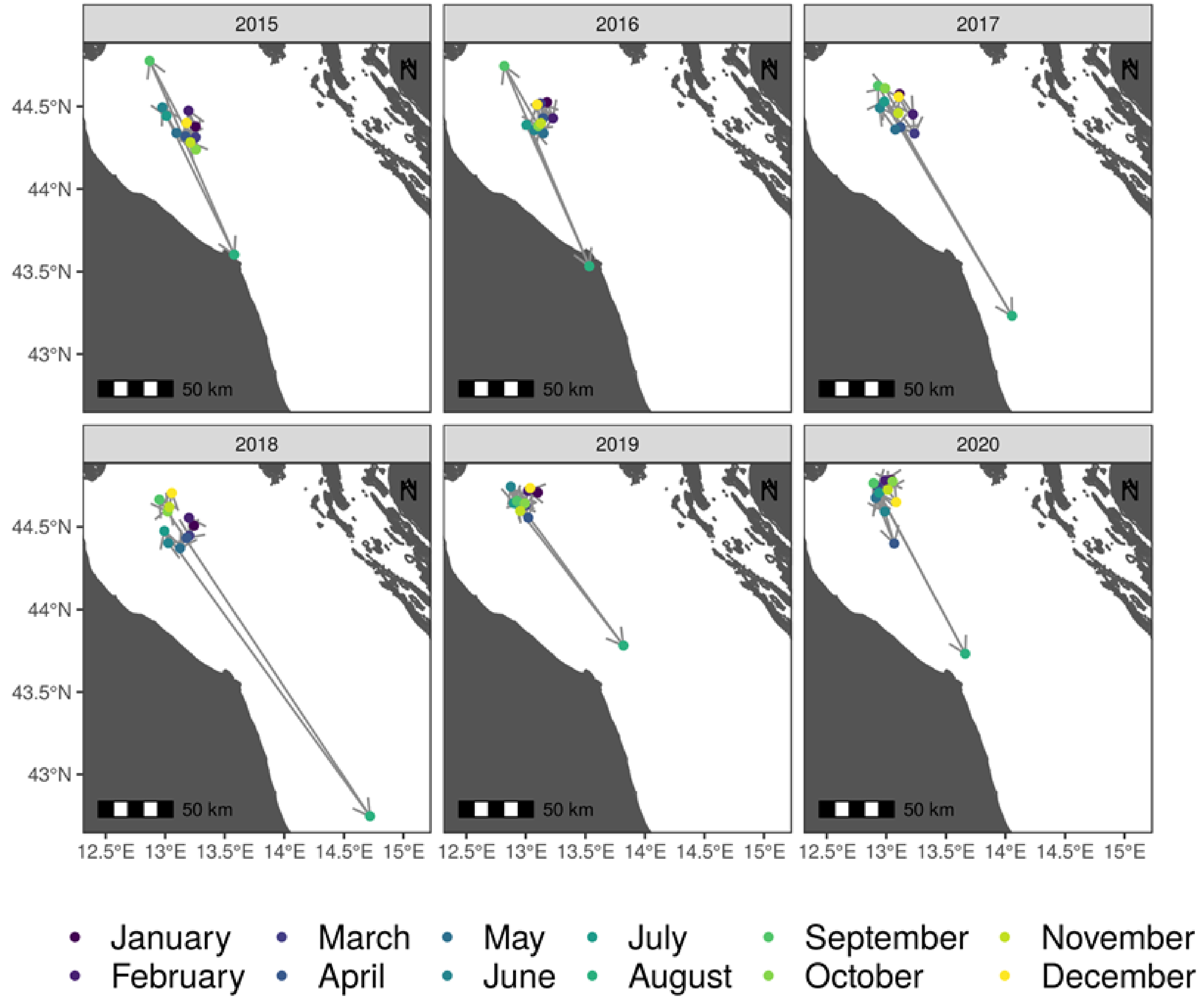

2015
2016
2017
2018
2019
2020
44.5°N
44°N
43.5°N
43°N
12.5°E
13°E
13.5°E
14°E
14.5°E
15°E
50 km
N
January
February
March
April
May
June
July
August
September
October
November
December

PS

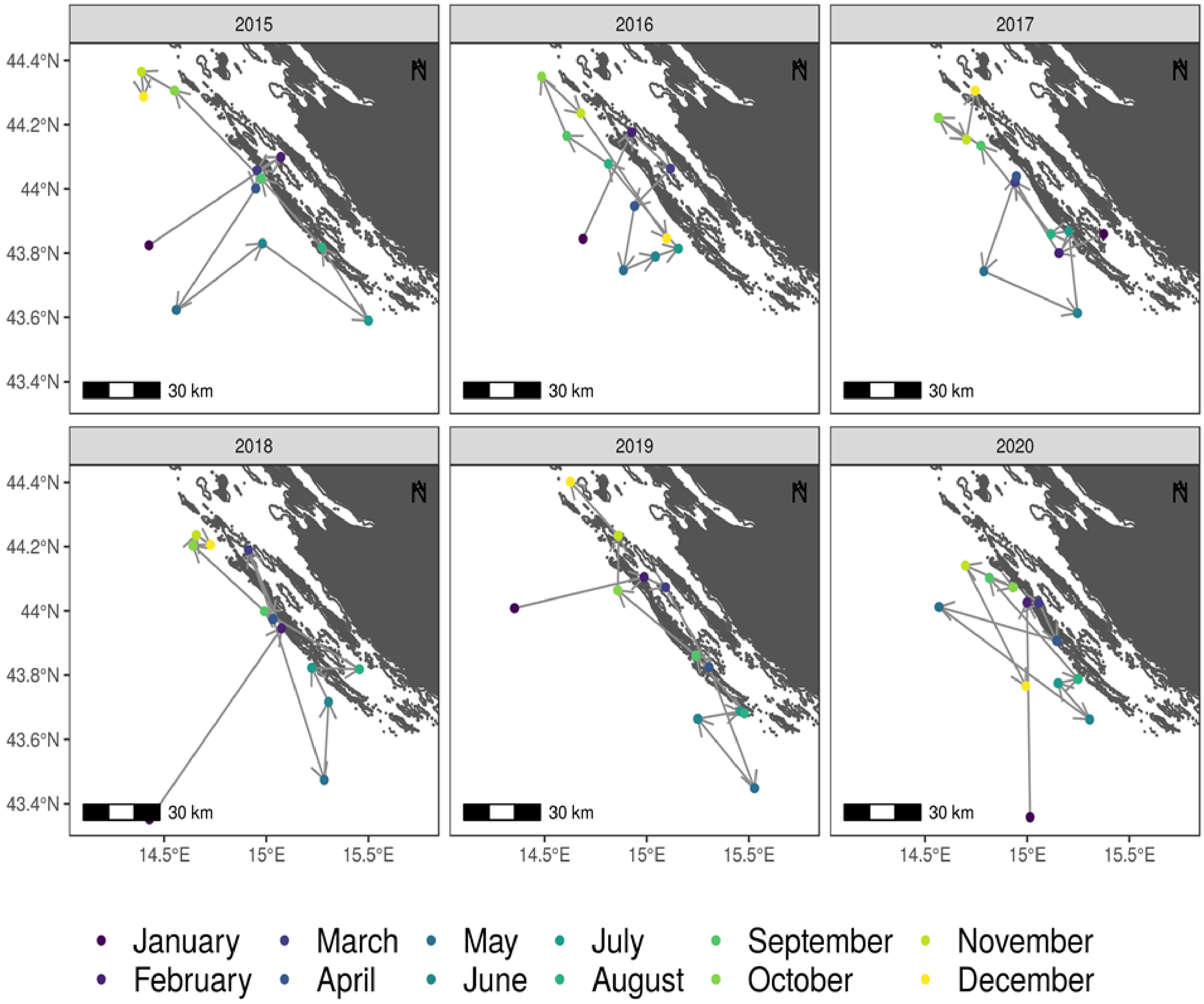
2015
2016
2017
2018
2019
2020
44.4°N
44.2°N
44°N
43.8°N
43.6°N
43.4°N
14.5°E
15°E
15.5°E
30 km
January
February
March
April
May
June
July
August
September
October
November
December

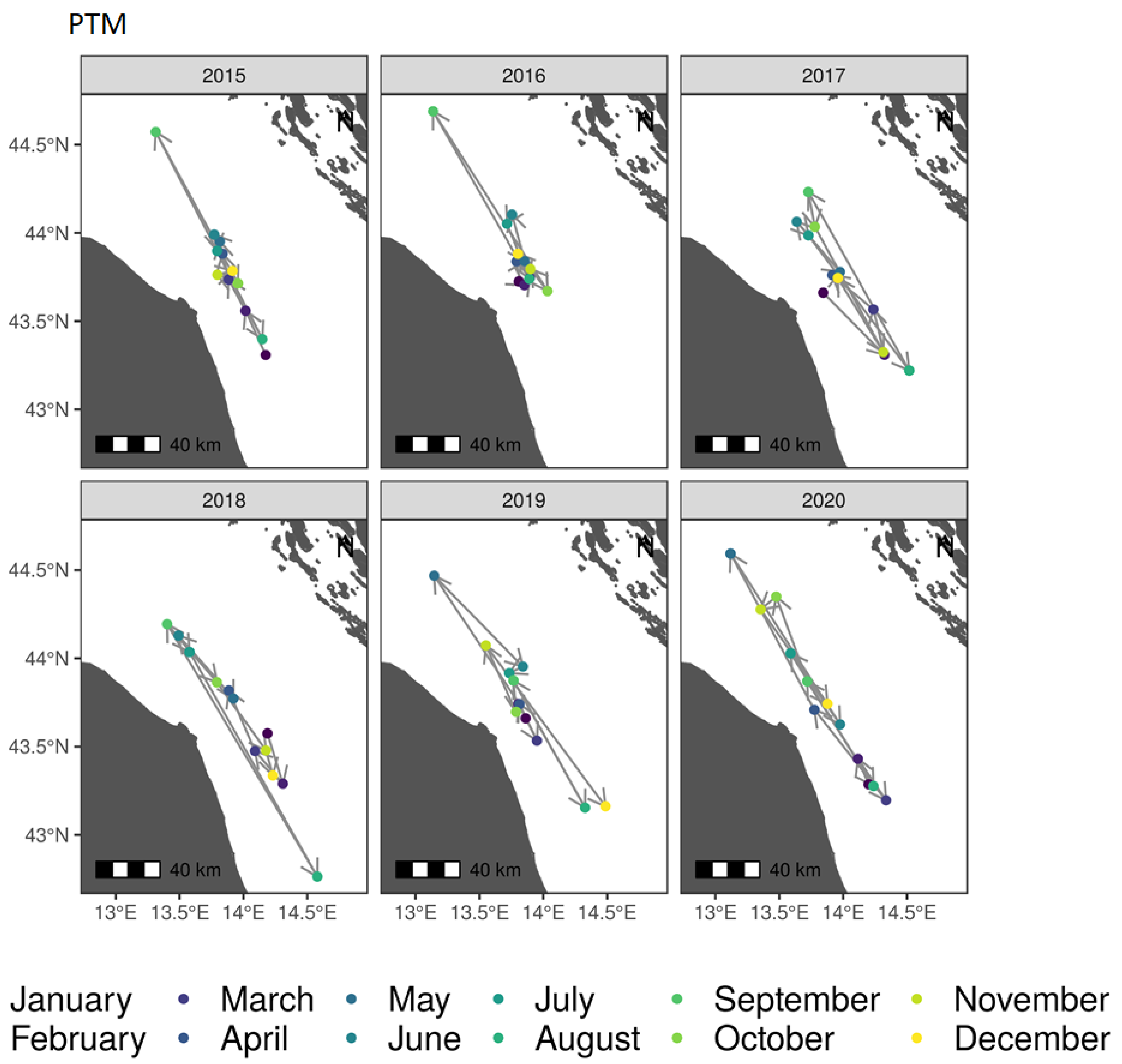
PTM
2015
2016
2017
2018
2019
2020
44.5°N
44°N
43.5°N
43°N
13°E
13.5°E
14°E
14.5°E
40 km
January
February
March
April
May
June
July
August
September
October
November
December

OTB

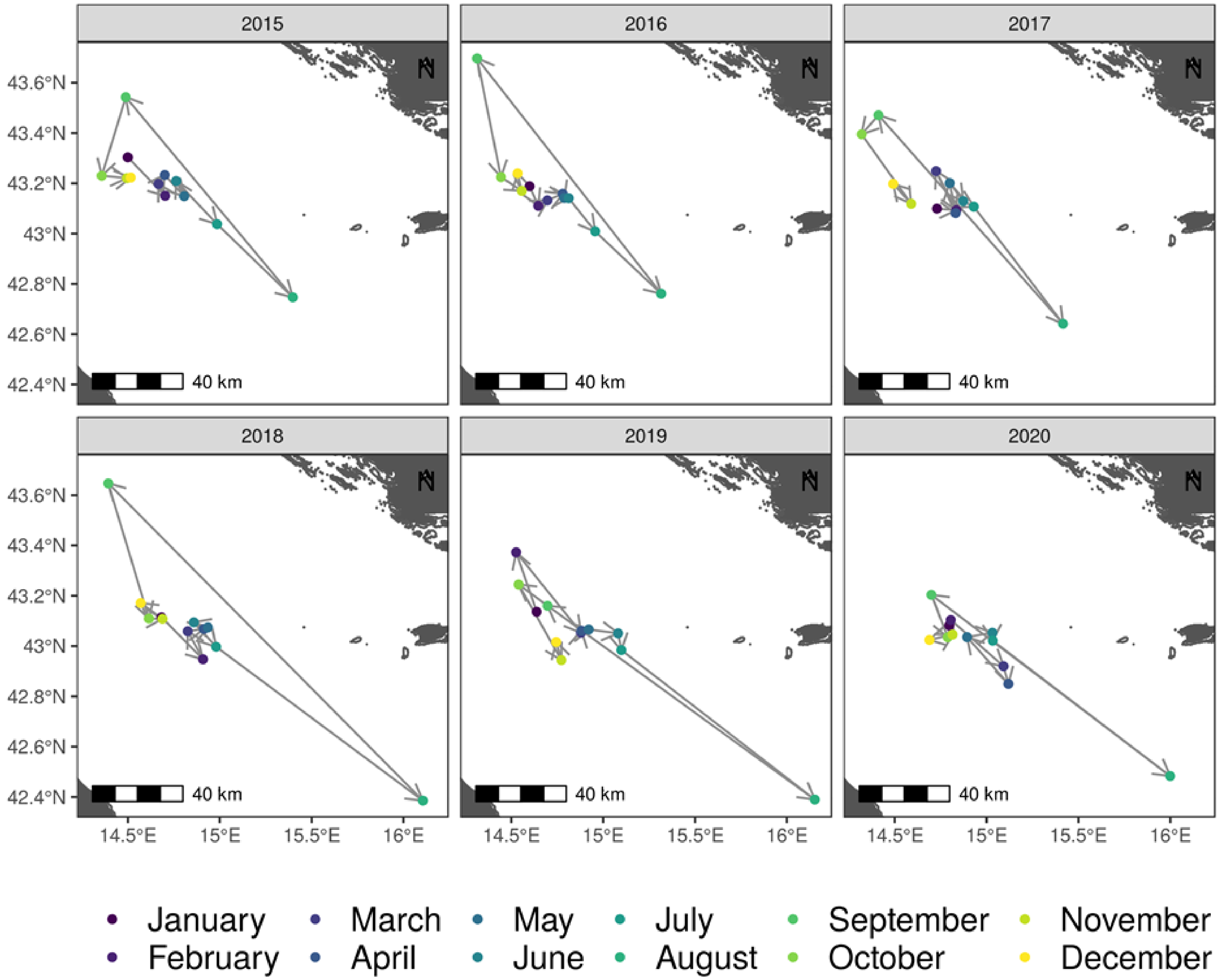
2015
2016
2017
2018
2019
2020
43.6°N
43.4°N
43.2°N
43°N
42.8°N
42.6°N
42.4°N
14.5°E
15°E
15.5°E
16°E
40 km
January
February
March
April
May
June
July
August
September
October
November
December

S3 - Total annual fishing effort (hours) in locations with medium and high concentrations of endangered, threatened and protected (ETP) species

All gears types

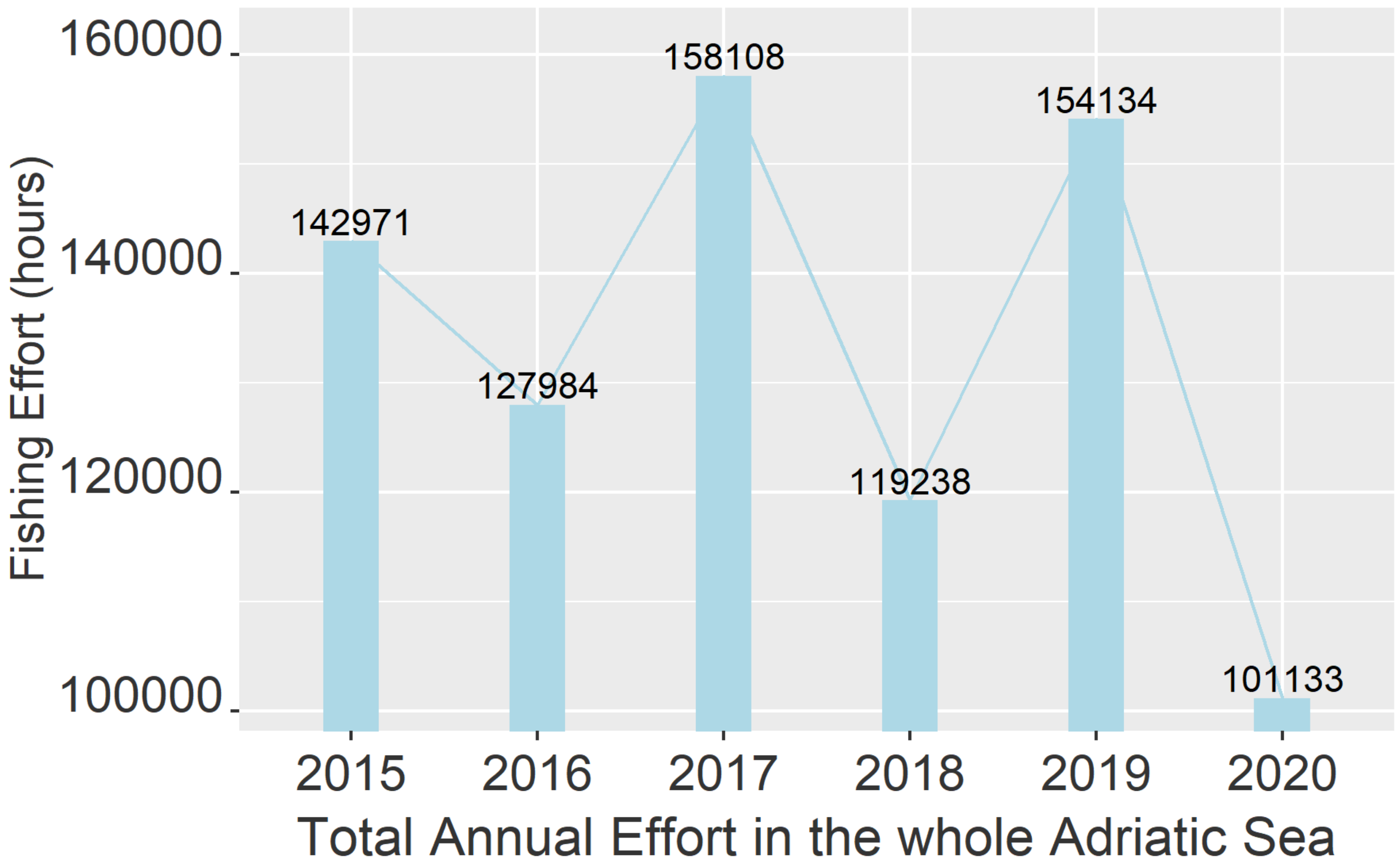

TBB

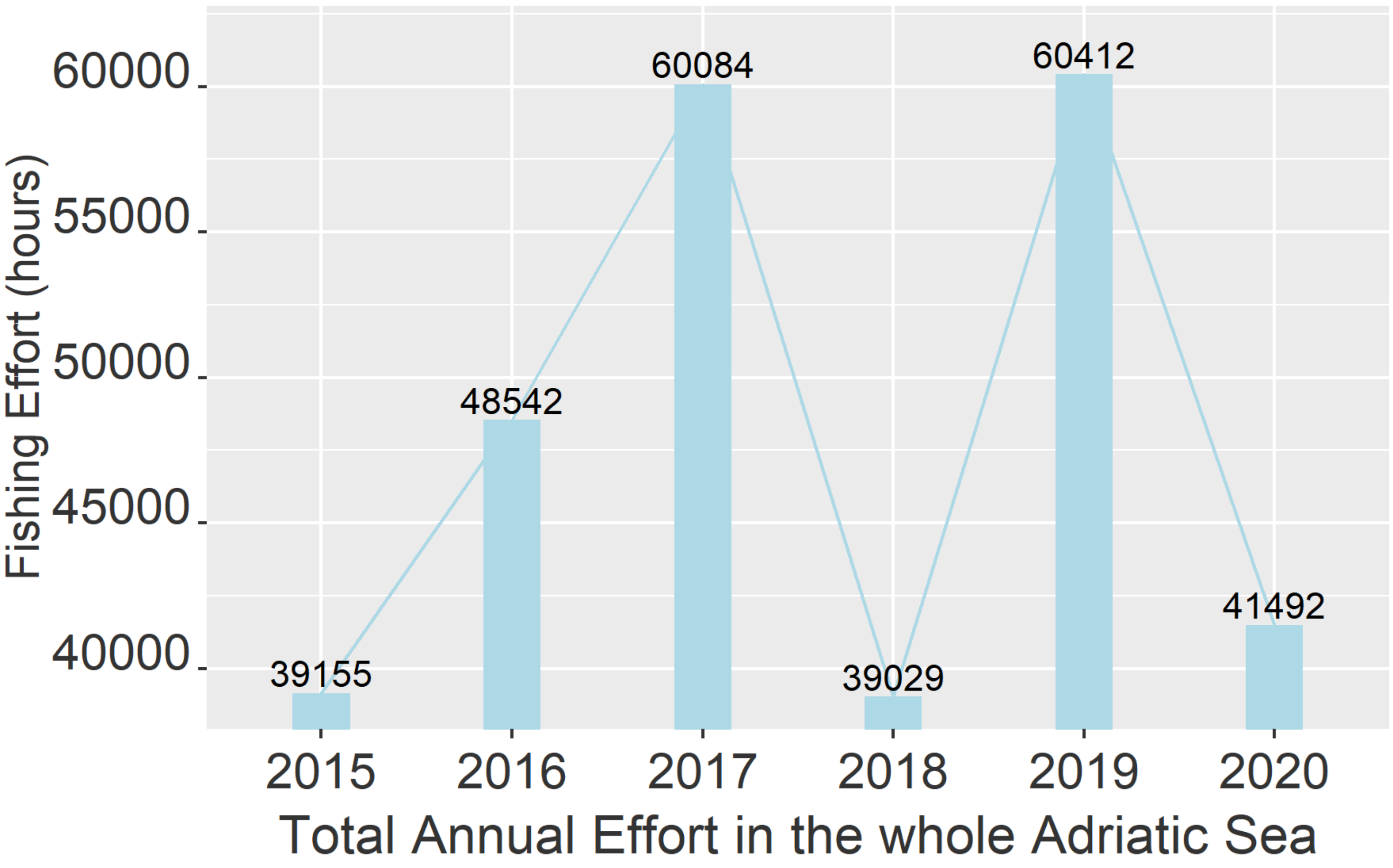

Fishing Effort (hours)
60000
55000
50000
45000
40000
39155
48542
60084
39029
60412
41492
2015
2016
2017
2018
2019
2020
Total Annual Effort in the whole Adriatic Sea

PS

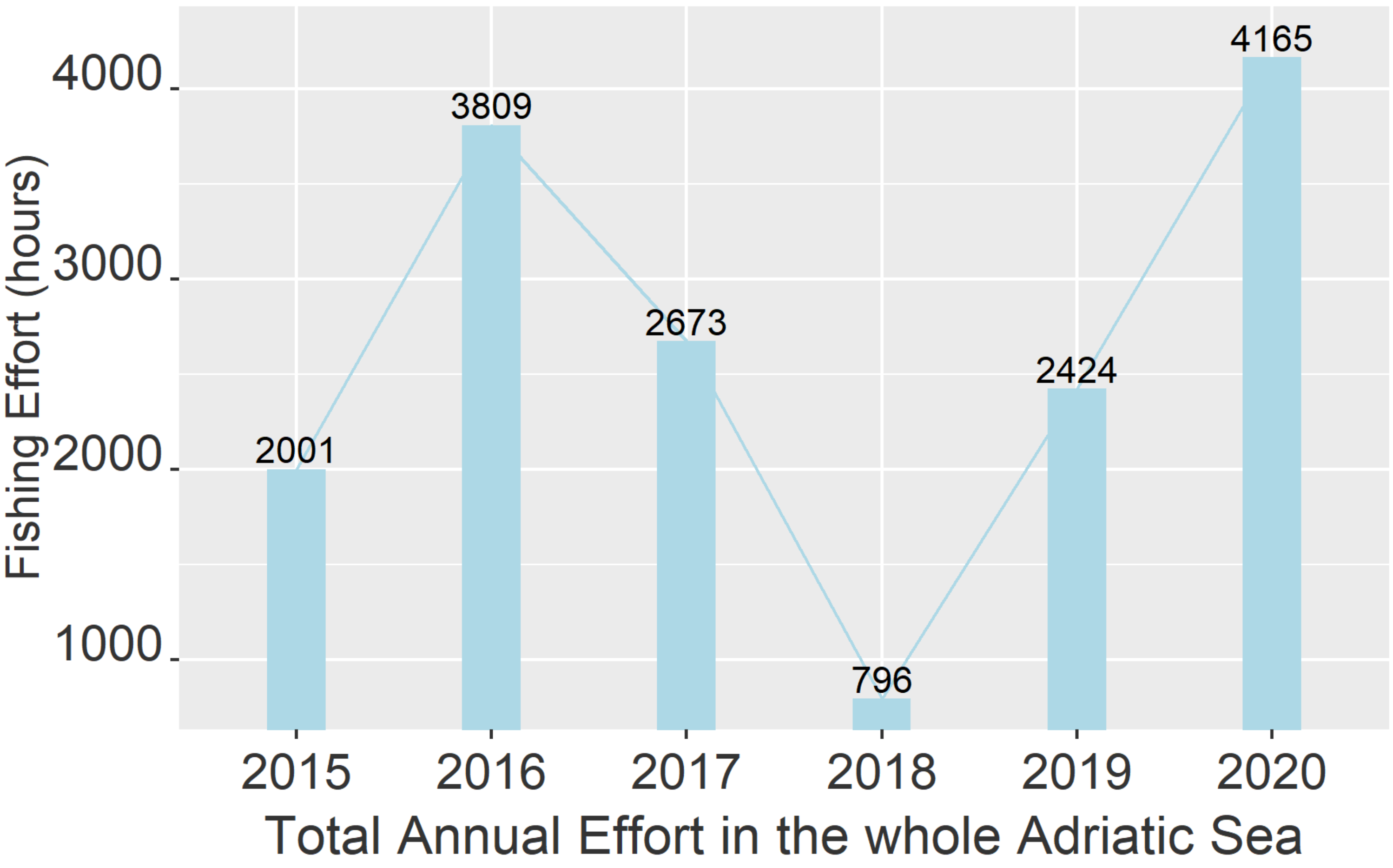
Fishing Effort (hours)
4000
3000
2000
1000
2001
3809
2673
796
2424
4165
2015
2016
2017
2018
2019
2020
Total Annual Effort in the whole Adriatic Sea

PTM

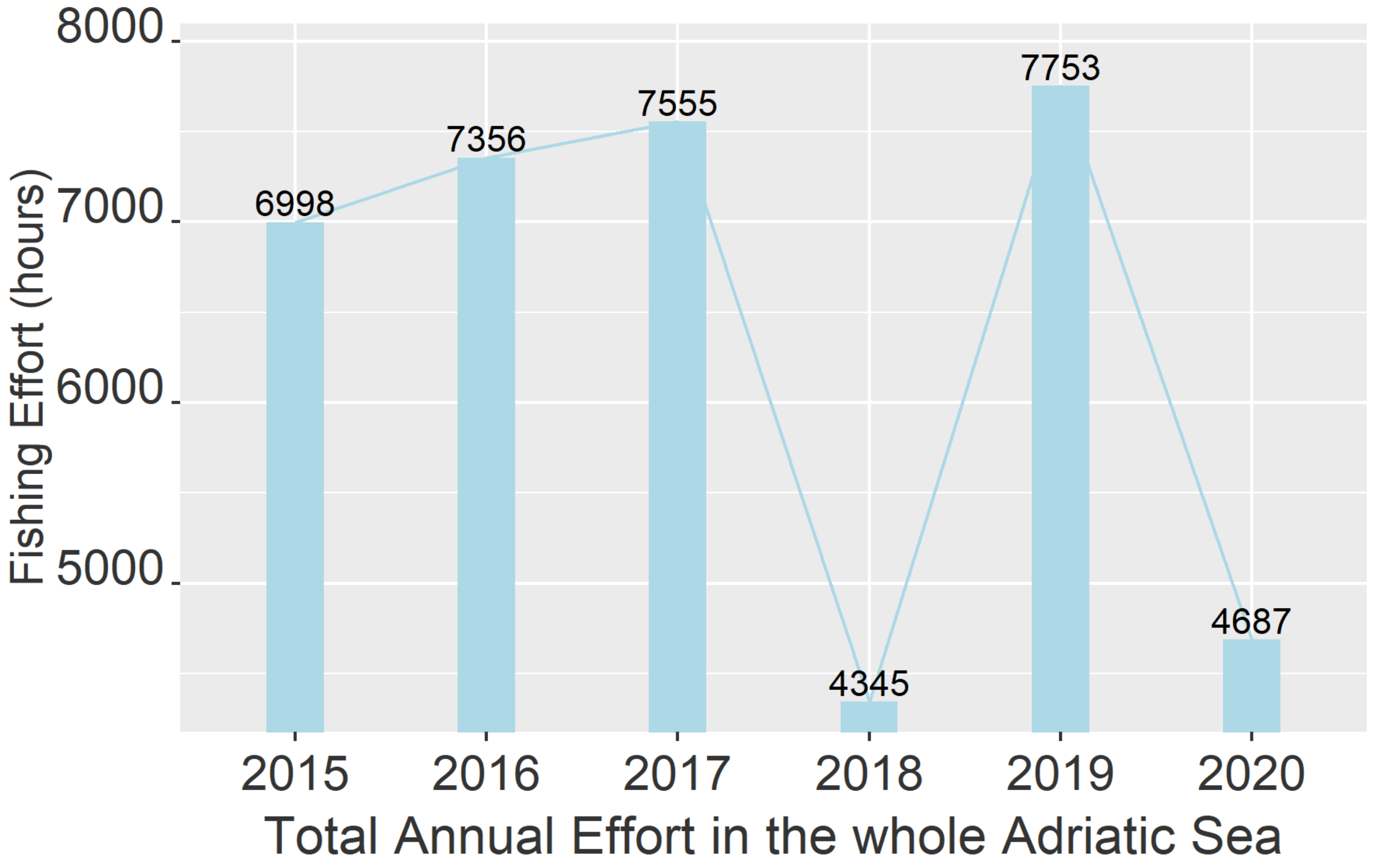

8000
7000
6000
5000
Fishing Effort (hours)
6998
7356
7555
4345
7753
4687
2015
2016
2017
2018
2019
2020
Total Annual Effort in the whole Adriatic Sea

OTB

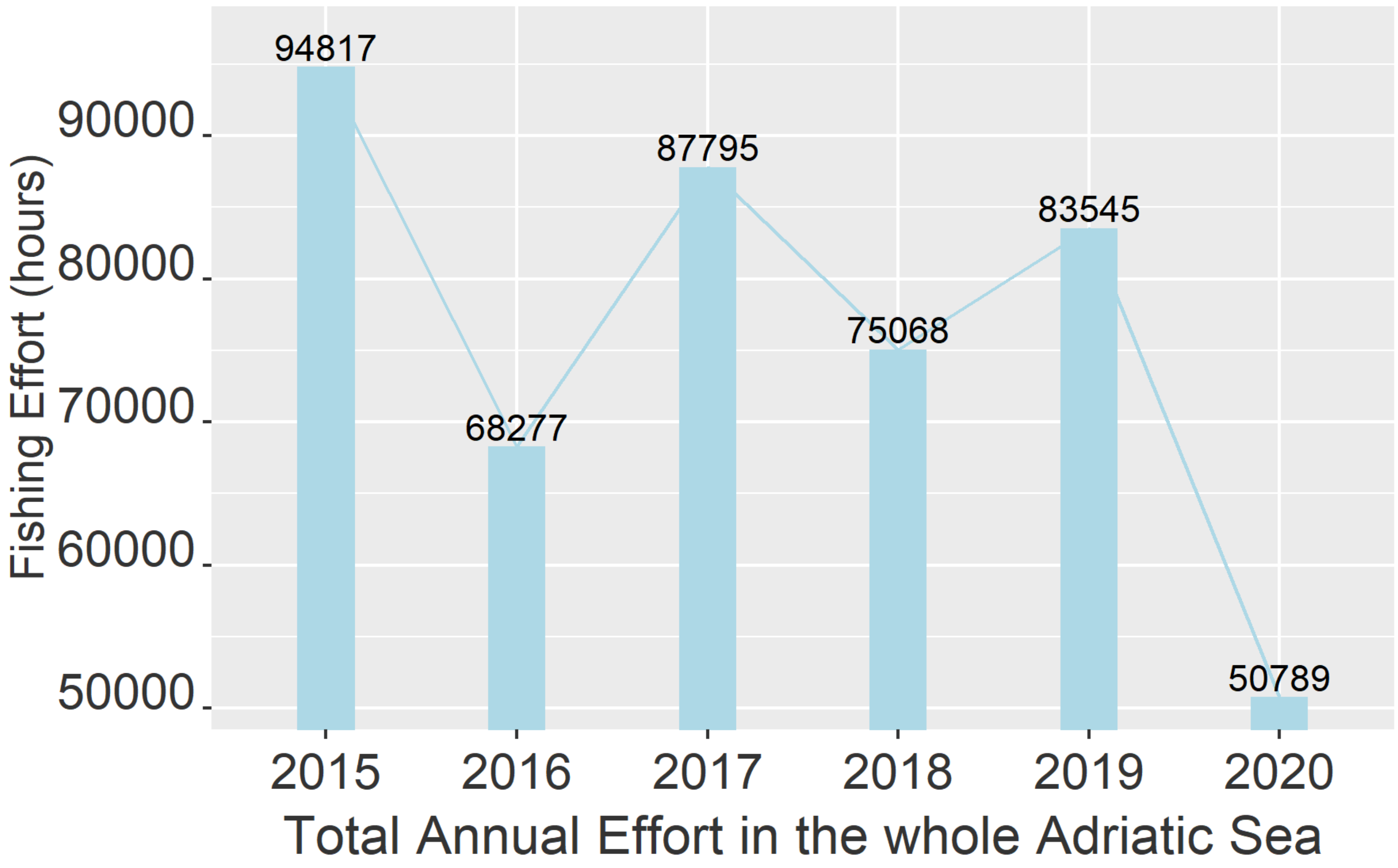

94817
68277
87795
75068
83545
50789
90000
80000
70000
60000
50000
Fishing Effort (hours)
2015
2016
2017
2018
2019
2020
Total Annual Effort in the whole Adriatic Sea

S4 - Total monthly fishing effort (hours) in locations with medium and high concentrations of endangered, threatened and protected (ETP) species

All gears types

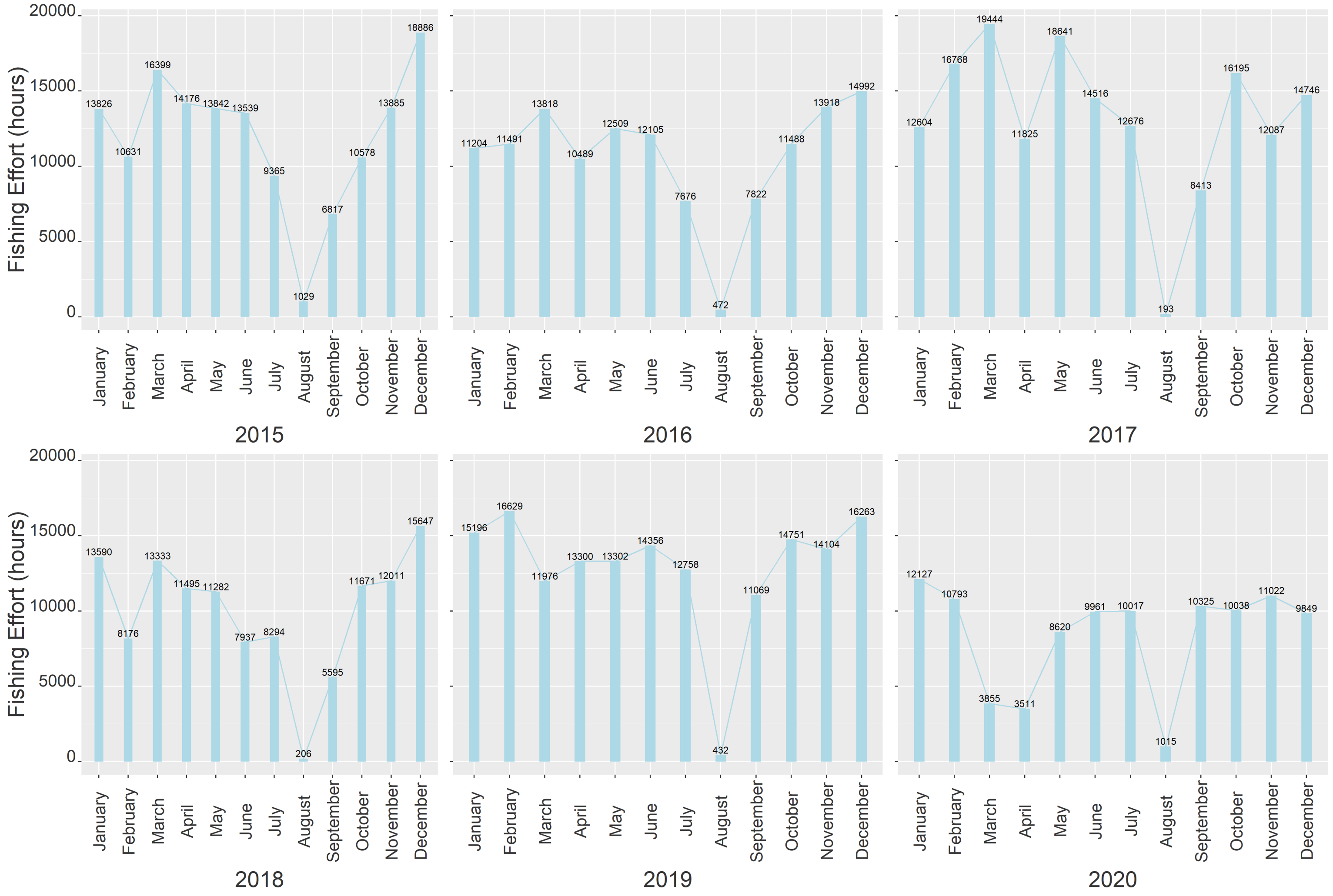

TBB

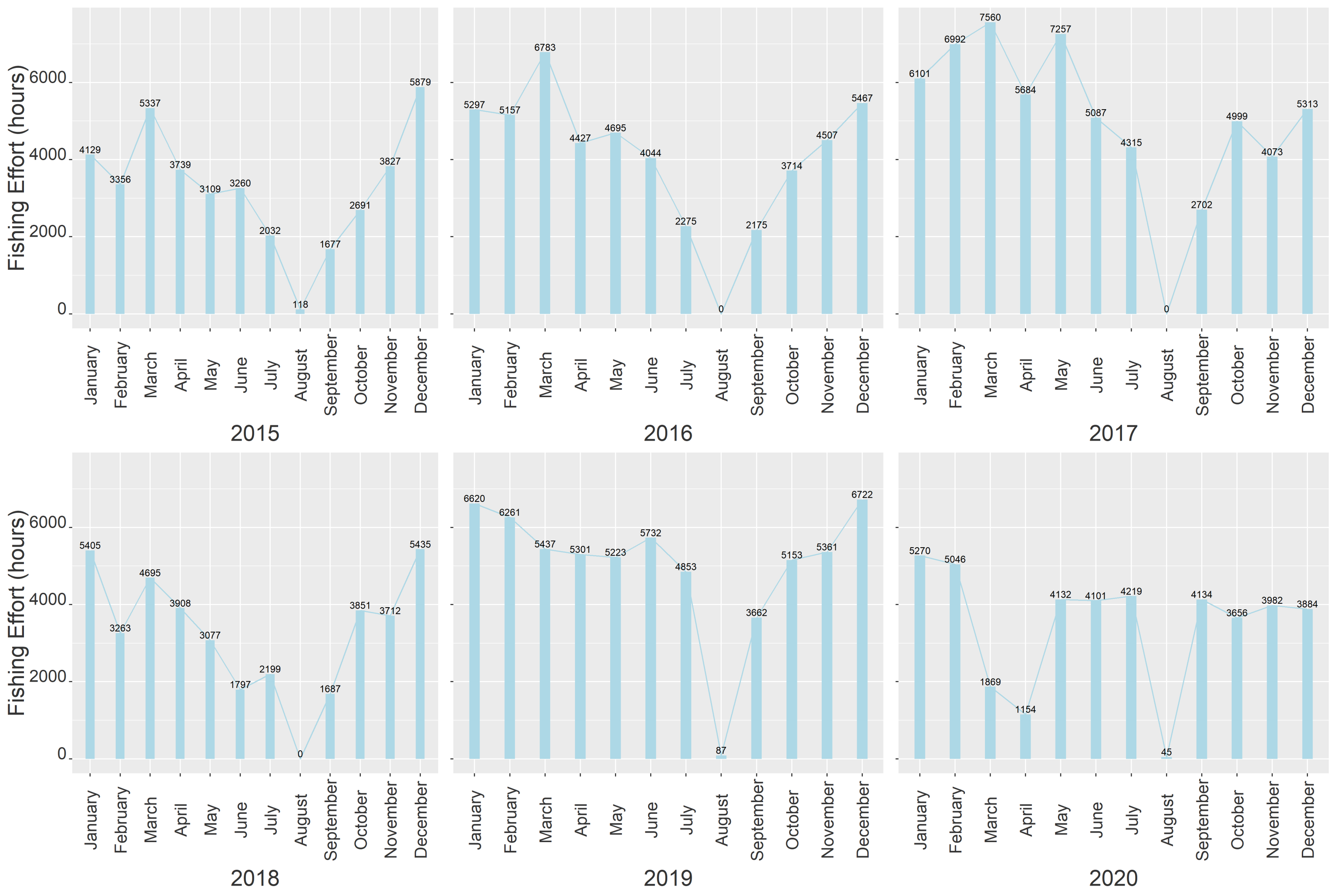

Fishing Effort (hours)
0
2000
4000
6000
January
February
March
April
May
June
July
August
September
October
November
December
2015
4129 3356 5337 3739 3109 3260 2032 118 1677 2691 3827 5879
2016
5297 5157 6783 4427 4695 4044 2275 0 2175 3714 4507 5467
2017
6101 6992 7560 5684 7257 5087 4315 0 2702 4999 4073 5313
2018
5405 3263 4695 3908 3077 1797 2199 0 1687 3851 3712 5435
2019
6620 6261 5437 5301 5223 5732 4853 87 3662 5153 5361 6722
2020
5270 5046 1869 1154 4132 4101 4219 45 4134 3656 3982 3884

PS

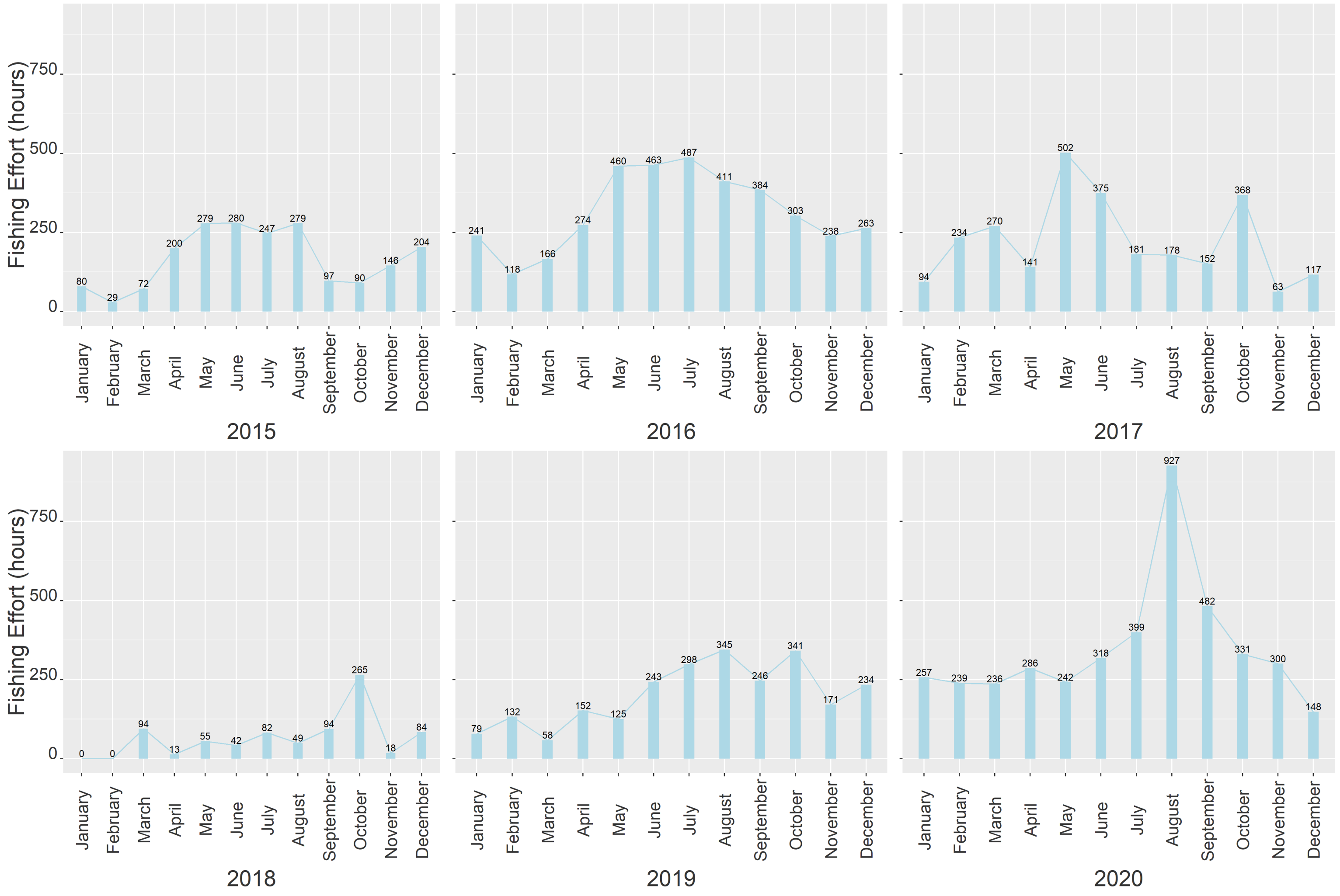

Fishing Effort (hours)
750
500
250
0
January
February
March
April
May
June
July
August
September
October
November
December
2015
80
29
72
200
279
280
247
279
97
90
146
204
2016
241
118
166
274
460
463
487
411
384
303
238
263
2017
94
234
270
141
502
375
181
178
152
368
63
117
2018
0
0
94
13
55
42
82
49
94
265
18
84
2019
79
132
58
152
125
243
298
345
246
341
171
234
2020
257
239
236
286
242
318
399
927
482
331
300
148

PTM

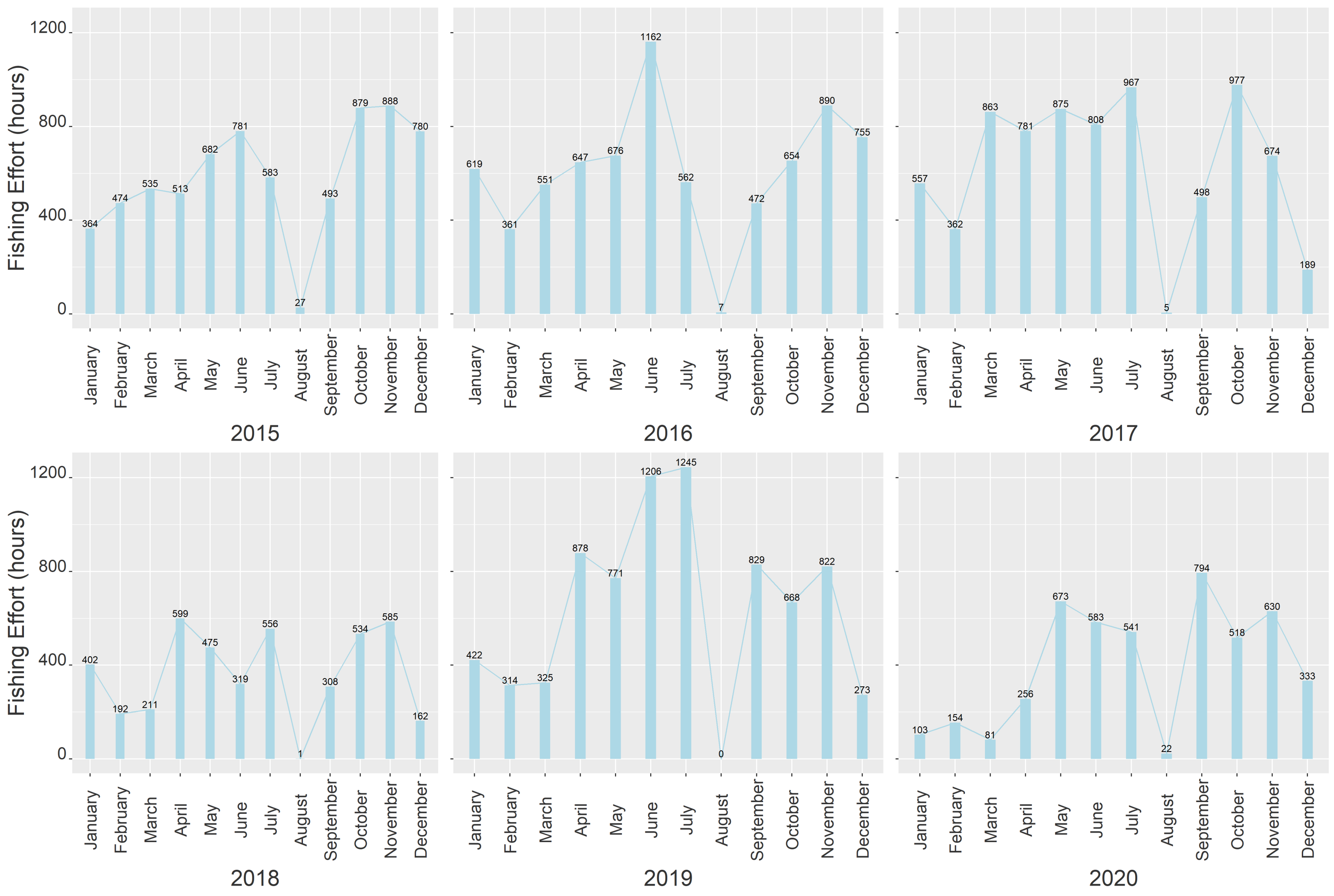

Fishing Effort (hours)
2015
364 474 535 513 682 781 583 27 493 879 888 780
2016
619 361 551 647 676 1162 562 7 472 654 890 755
2017
557 362 863 781 875 808 967 5 498 977 674 189
2018
402 192 211 599 475 319 556 1 308 534 585 162
2019
422 314 325 878 771 1206 1245 0 829 668 822 273
2020
103 154 81 256 673 583 541 22 794 518 630 333
January February March April May June July August September October November December
0 400 800 1200

OTB

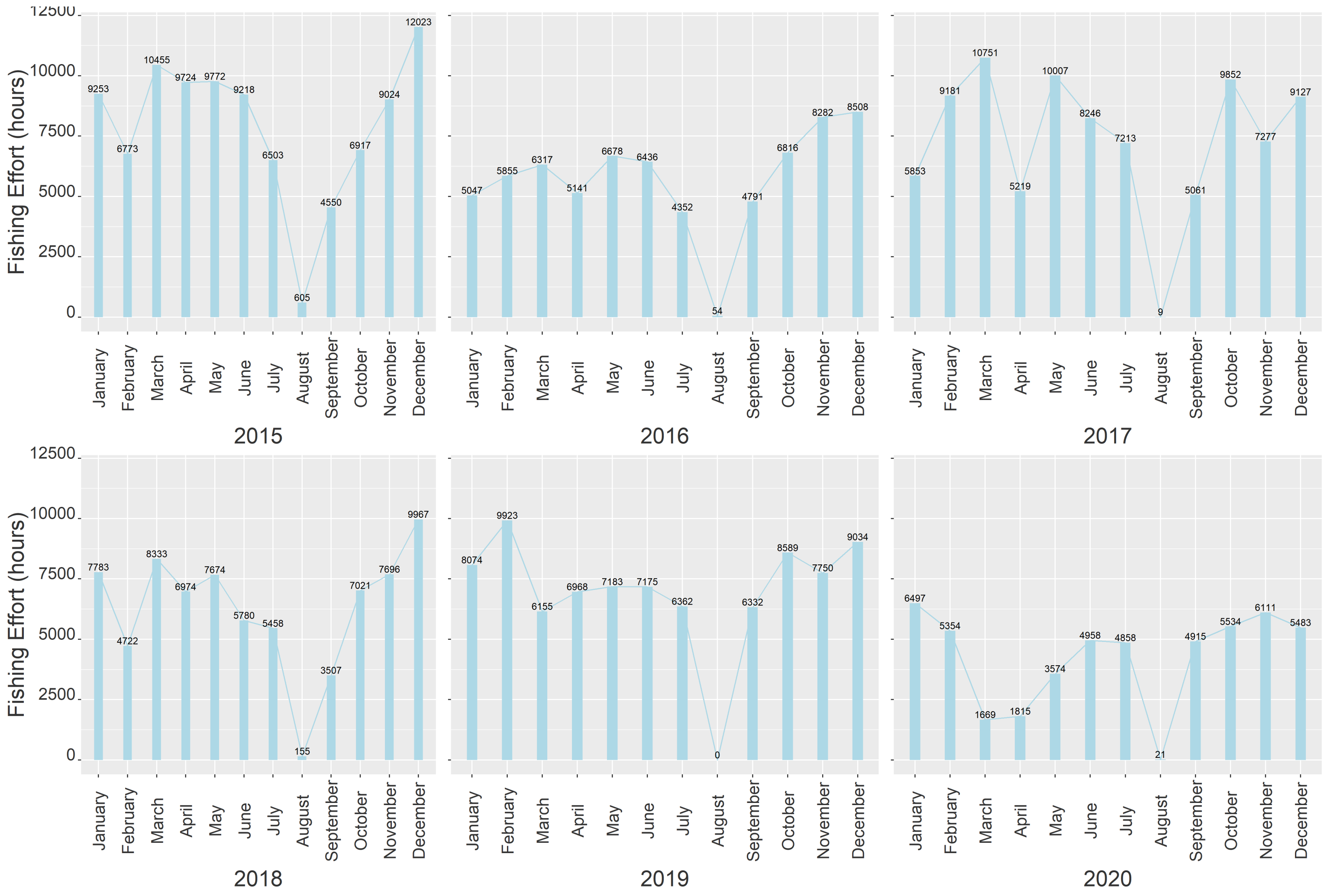

Fishing Effort (hours)
2015
9253 6773 10455 9724 9772 9218 6503 605 4550 6917 9024 12023
2016
5047 5855 6317 5141 6678 6436 4352 54 4791 6816 8282 8508
2017
5853 9181 10751 5219 10007 8246 7213 9 5061 9852 7277 9127
2018
7783 4722 8333 6974 7674 5780 5458 155 3507 7021 7696 9967
2019
8074 9923 6155 6968 7183 7175 6362 0 6332 8589 7750 9034
2020
6497 5354 1669 1815 3574 4958 4858 21 4915 5534 6111 5483
January February March April May June July August September October November December

S5 - Fleet assessment analysis – AMSY analysis charts

TBB

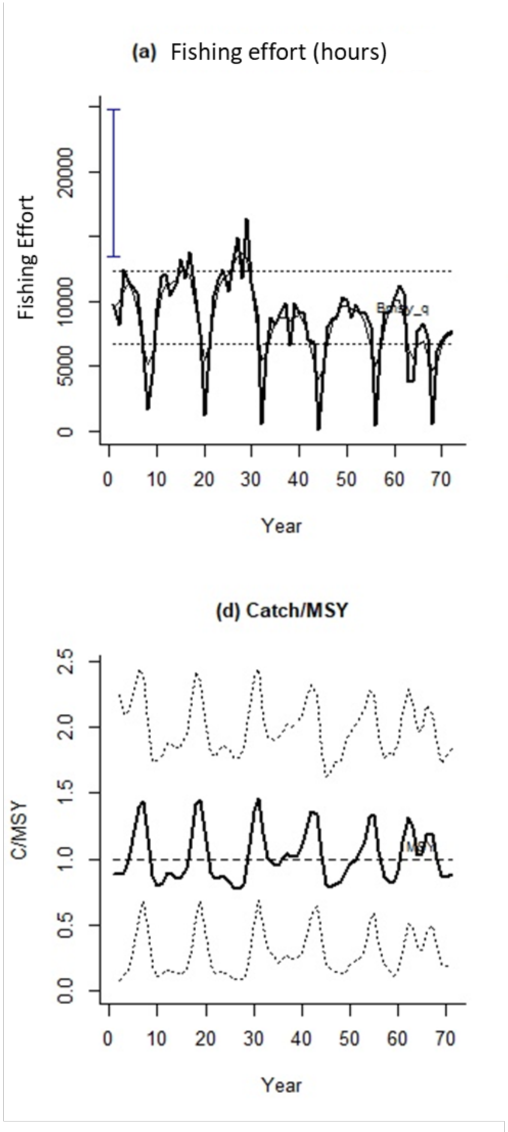

PS

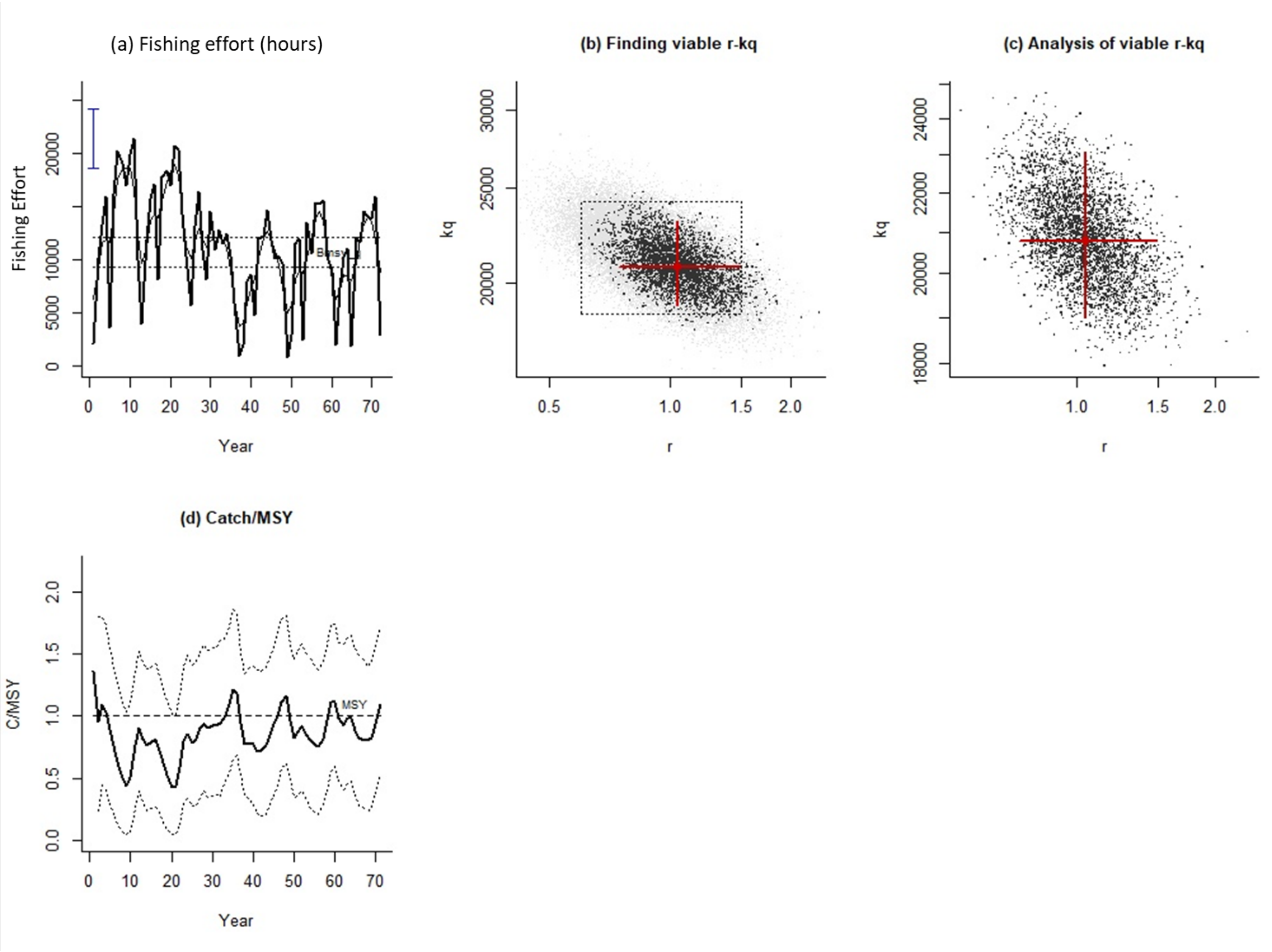

(a) Fishing effort (hours)
Fishing Effort
Bmsy
Year
(b) Finding viable r-kq
kq
r
(c) Analysis of viable r-kq
kq
r
(d) Catch/MSY
C/MSY
MSY
Year

PTM

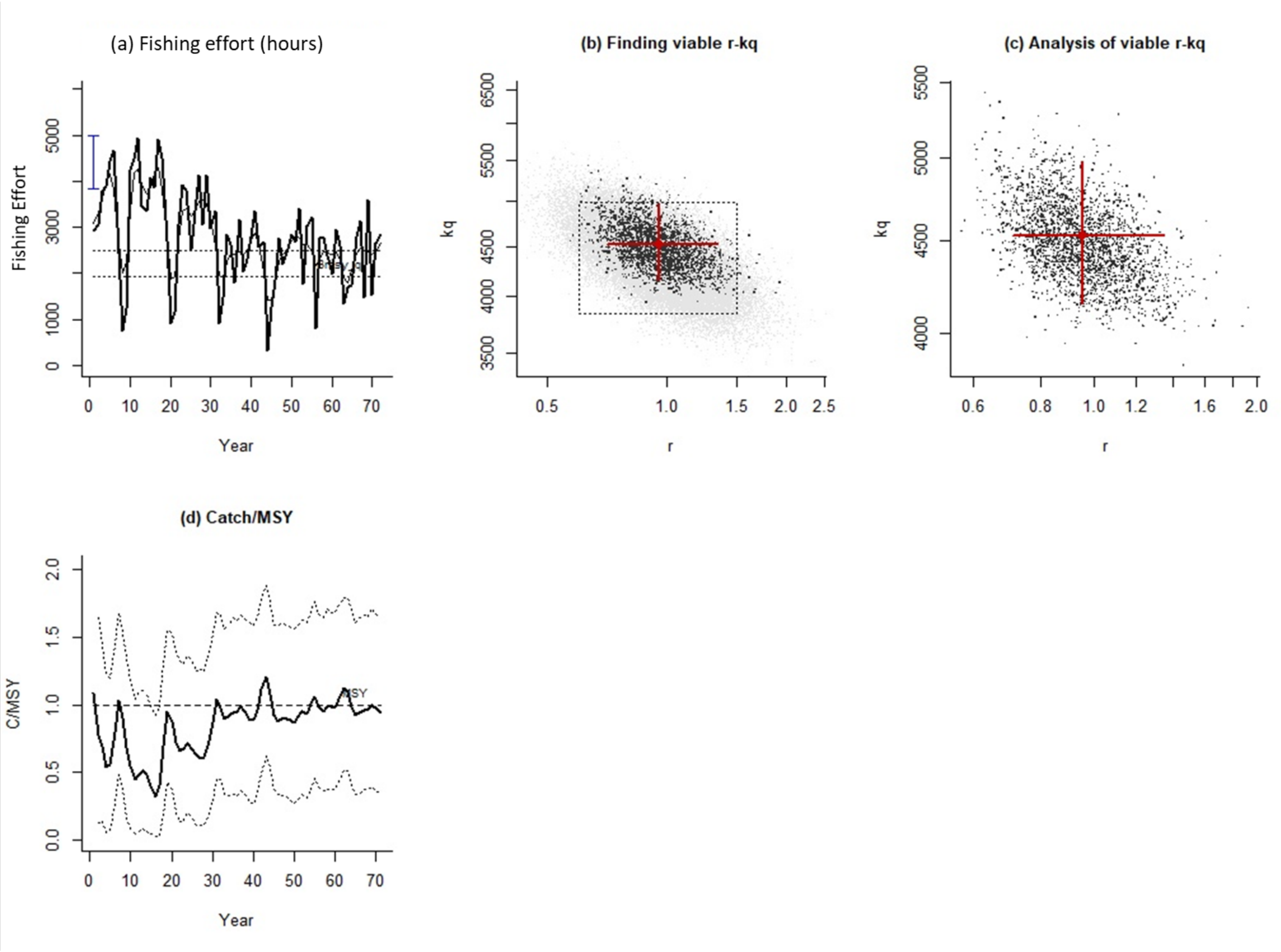

(a) Fishing effort (hours)
Fishing Effort
Year
(b) Finding viable r-kq
kq
r
(c) Analysis of viable r-kq
kq
r
(d) Catch/MSY
C/MSY
MSY
Year

OTB

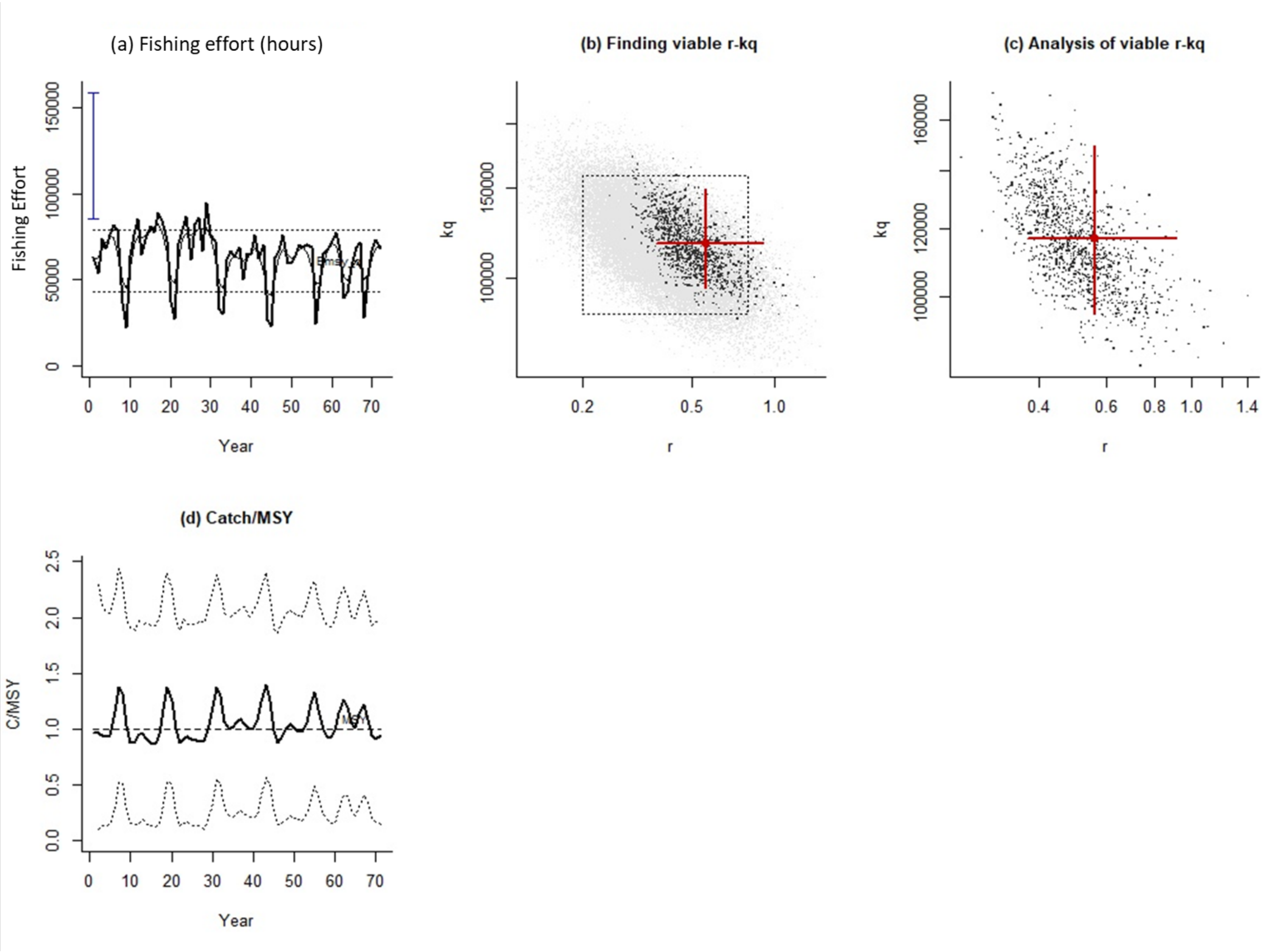
(a) Fishing effort (hours)
Fishing Effort
Year
(b) Finding viable r-kq
kq
r
(c) Analysis of viable r-kq
kq
r
(d) Catch/MSY
C/MSY
Year
